# Genomic features of uncultured methylotrophs in activated-sludge microbiomes grown under different enrichment procedures

**DOI:** 10.1038/srep26650

**Published:** 2016-05-25

**Authors:** Kazuki Fujinawa, Yusuke Asai, Morio Miyahara, Atsushi Kouzuma, Takashi Abe, Kazuya Watanabe

**Affiliations:** 1 School of Life Sciences, Tokyo University of Pharmacy and Life Sciences, Horinouchi, Hachioji, Tokyo, Japan; 2 Graduate School of Science and Technology, Niigata University, Niigata, Japan

## Abstract

Methylotrophs are organisms that are able to grow on C1 compounds as carbon and energy sources. They play important roles in the global carbon cycle and contribute largely to industrial wastewater treatment. To identify and characterize methylotrophs that are involved in methanol degradation in wastewater-treatment plants, methanol-fed activated-sludge (MAS) microbiomes were subjected to phylogenetic and metagenomic analyses, and genomic features of dominant methylotrophs in MAS were compared with those preferentially grown in laboratory enrichment cultures (LECs). These analyses consistently indicate that *Hyphomicrobium* plays important roles in MAS, while *Methylophilus* occurred predominantly in LECs. Comparative analyses of bin genomes reconstructed for the *Hyphomicrobium* and *Methylophilus* methylotrophs suggest that they have different C1-assimilation pathways. In addition, function-module analyses suggest that their cell-surface structures are different. Comparison of the MAS bin genome with genomes of closely related *Hyphomicrobium* isolates suggests that genes unnecessary in MAS (for instance, genes for anaerobic respiration) have been lost from the genome of the dominant methylotroph. We suggest that genomic features and coded functions in the MAS bin genome provide us with insights into how this methylotroph adapts to activated-sludge ecosystems.

Methylotrophs are organisms that can utilize C1 compounds, such as methanol and methylamine, as the sole carbon and energy sources^1^. They occur in a variety of natural habitats, including soil, freshwater and marine environments^2^. In addition, they are found in man-made microbial ecosystems, such as wastewater-treatment facilities^3^. Since methanol is widely used as a material in various industrial applications, such as the production of formaldehyde and esters^4^ and generated as a byproduct in various industrial processes, such as pulp mills and coal gasification plants^5^, methylotrophs are considered important in industrial wastewater-treatment facilities.

Aerobic methylotrophs have been isolated from a variety of environments^6^, and they are subjected to genetic and physiological studies to understand how microbes metabolize C1 compounds^7^. These studies have identified several different types of methanol dehydrogenases, such as MxaFI and XoxF, to be exploited by methylotrophs^7^. In addition, studies have also identified several different C1 assimilation pathways that methylotrophs use for their growth^7^. On the other hand, molecular ecological analyses have been performed to characterize natural methylotrophs without cultivation, and these studies have suggested that yet-uncultured methylotrophs are abundantly present in natural ecosystems^8^. For instance, PCR detection of *mxaF* genes has revealed that novel groups of methylotrophs are present in bog peat cores^9^. Recently, metagenomics have been applied to uncover genomic features of yet-uncultured methylotrophs in freshwater microcosms^10^.

Microbes with a particular function have conventionally been isolated from microbiomes after enrichment in liquid media containing particular substrates^11^. Batch and continuous cultures have been used for this purpose, and physiological and genetic traits of bacteria obtained after different enrichment/isolation procedures have been compared^12^,^13^. These studies have shown that isolates obtained after batch enrichment cultures exhibit peculiar features (e.g., rapid growth in laboratory media) and do not represent microbes that are abundantly present in natural microbiomes. To date, however, most methylotrophs have been isolated after enrichment in batch cultures containing C1 compounds, e.g., methanol, as the sole carbon and energy source^6^,^11^.

Given that our knowledge on methylotrophy relies mostly on physiological and genetic studies of isolated methylotrophs, it is reasonable to speculate that the natural diversity of methylotrophy has not yet been fully understood. The present study was therefore undertaken to better understand methylotrophs that play important roles for methanol degradation in activated-sludge wastewater-treatment plants. In order to characterize dominant methylotrophs in activated sludge, metagenomics were used to analyze methanol-fed activated-sludge microbiomes, and genomic features of dominant methylotrophs were compared with those preferentially grown in batch and continuous cultures.

## Results and Discussion

### Enrichment of methylotrophs

A laboratory activated-sludge reactor was inoculated with activated sludge obtained from a sewage-treatment plant (sewage activated sludge, SAS) and continuously supplied with a medium containing methanol (500 mg L^−1^) as the sole carbon and energy source for obtaining methanol-acclimatized activated sludge (MAS). In parallel, a fermenter was inoculated with SAS and continuously suppled with the methanol medium for obtaining a methanol-acclimatized continuous-culture enrichment (MCC). These two systems were the same in methanol loading, while biomass concentrations and growth rates (doubling times) were largely different in terms of the presence of a sludge-retaining mechanism in the activated-sludge reactor. Methanol was not detected in effluents from the MAS reactor and MCC fermenter (below 1 mg L^−1^), while oxygen concentrations were 2 mg L^−1^ or higher. In addition, the methanol medium was also inoculated with SAS for growing methylotrophs in batch cultures. A resultant microbial culture was repeatedly (5 times) transferred to the fresh methanol medium for obtaining a methanol-acclimatized batch-culture enrichment (MBC). In these batch enrichments, methanol was completely degraded within 24 hours (oxygen was not measured). Operational characteristics of these four microbiomes are summarized in Table 1. Notably, MAS, MCC and MBC were grown in the same medium but under different generation times. In addition, similar to SAS, microbes in MAS formed flocs to persist in the activated-sludge unit, while such microbial flocs were not observed in MCC and MBC. It was therefore expected that methylotrophs were enriched in MAS under physical conditions (e.g., growth rate and substrate/oxygen availability) that were similar to sewage-treatment plants.

### PCR-based phylogenetic analyses

**Figure 1:**
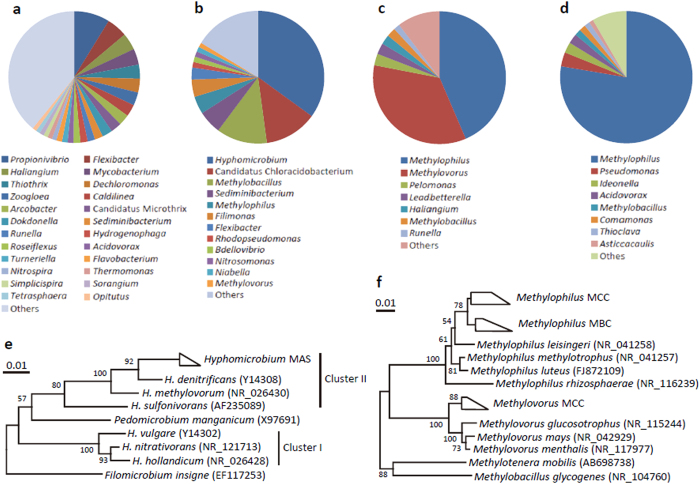
Phylogenetic characterization of bacteria present in the microbiomes based on 16S rRNA gene sequences. Relative abundances of major bacterial genera in the SAS (**a**), MAS (**b**), MBC (**c**) and MCC (**d**) microbiomes are shown in circle charts. Neighbor-joining trees show phylogenetic relationships among species in the genera *Hyphomicrobium* (**e**), *Methylophilus* and *Methylovorus* (**f**). Bootstrap values (100 trials, only >50 are shown) are indicated at branching points. Bars indicate 1% sequence divergence. Accession numbers are shown in parentheses.

Bacterial 16S rRNA-gene fragments were PCR-amplified from metagenomes extracted from the above-described four microbiomes, and amplicons were pyro-sequenced for phylogenetic characterization of bacteria occurring there (Fig. 1). It is shown that methylotroph-related taxa^2^ were highly enriched in MCC (80% of the total) and MBC (82%). In particular, bacteria affiliated with the genus *Methylophilus* made up over 75% of the total bacteria in MBC (panel d in Fig. 1). In MCC, each of two bacterial groups affiliated with the genera *Methylophilus* and *Methylovorus* made up over 30% of the total bacteria (panels c and f in Fig. 1). In contrast, in MAS, bacteria affiliated with the genus *Hyphomicrobium* was abundantly detected and made up approximately 30% of the total bacteria (panels b and e in Fig. 1). *Hyphomicrobium* is known to include methylotrophs^2^ and has been detected in sludge and biofilms in water-treatment facilities^14^,^15^.

In a previous study, clone-library analyses and whole-cell fluorescence hybridization were carried out to analyze *Hyphomicrobium* populations in activated sludge receiving industrial wastewater containing methanol^14^, and it has been suggested that cluster II *Hyphomicrobium* is more abundant than cluster I in activated sludge^14^. Cluster II *Hyphomicrobium* detected in that study was closely related to *H. denitrificans*^16^ isolated from mud^17^. The present study also detected members of the cluster-II *Hyphomicrobium* (those relevant to wastewater treatment) as the major population in the methanol-fed activated sludge (Fig. 1e), and these are closely related to *H. denitrificans*^17^. We were therefore interested in characterizing this *Hyphomicrobium* to gain insights into how it dominated in the MAS microbiome. It is also considered interesting to comparatively analyze this *Hyphomicrobium* with the putative methylotrophs overgrowing in MCC and MBC, for which comparative metagenomics were considered to be the primary step.

### Bin genomes reconstructed from metagenomes

**Figure 2:**
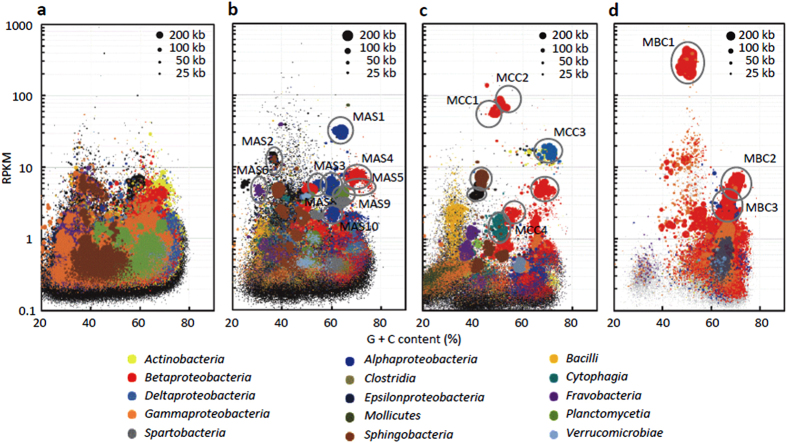
Distribution of contigs in bubble charts for the SAS (**a**), MAS (**b**), MBC (**c**) and MCC (**d**) microbiomes. Contigs are phylogenetically classified using colors, and contig lengths correspond to bubble sizes. Contigs selected for reconstructing bin genomes are circled.

In order to gain genomic insights into organisms abundantly present in the methanol-fed microbiomes, metagenomes extracted from these microbiomes were shotgun-sequenced, and reads were assembled to construct contigs. Data for the metagenome sequencing (Table 2) indicate that the number and total length of contigs are positively correlated with the evenness of a microbiome. Assembled contigs were mapped into bubble charts (Fig. 2) that were drawn based on G + C contents and RPKM (reads per kilobase per million mapped reads) values^18^. Comparisons of the bubble charts indicate that high-RPKM contigs appeared in the MCC and MBC metagenomes. Notably, a group of contigs with RPKM values of over 100 was present in the MBC chart; these contigs were considered to represent the dominant bacterium affiliated with *Methylophilus* (see Fig. 1d).

For bin-genome reconstruction, contigs were selected using the bubble charts (Fig. 2, indicated with circles), and bins were refined by subjecting selected contigs to tetranucleotide-frequency analyses^19^ and core-gene analyses (using 105 universal single-copy genes)^20^. Accordingly, no bins contain multiple copies of same core genes. This contig-selection strategy for binning has been used in recent metagenomics studies^21^. Bin genomes reconstructed from the MAS, MCC and MBC metagenomes are evaluated based on the completeness as estimated from the presence and absence of core genes^20^ and relative abundance as calculated based on a number of reads used for a bin genome per the total number of reads. Phylogenetic coherence among contigs categorized in a bin genome was evaluated by analyzing the universal single-copy genes. We finally constructed 17 bin genomes whose completeness values are over 70% (Supplementary Table S1). Among them, 6 bin genomes were found to code for methanol dehydrogenases (supplementary Fig. S1) and C1-metabolizing pathways (Table 3), suggesting that these represent methylotrophs. The citrate cycle and cytochrome oxidases are coded in the 6 bin genomes, suggesting that all these represent aerobic methylotrophs. Since the aim of the present study was to compare at the genome level the dominant methylotroph in activated sludge and those enriched in the laboratory cultures, we conclude that the manual binning was successful in the present study for reconstructing the high-quality bin genomes for these organisms. For reconstructing bin genomes of relatively minor species, however, manual binning should be cautiously used in terms of reproducibility and accuracy.

**Figure 3:**
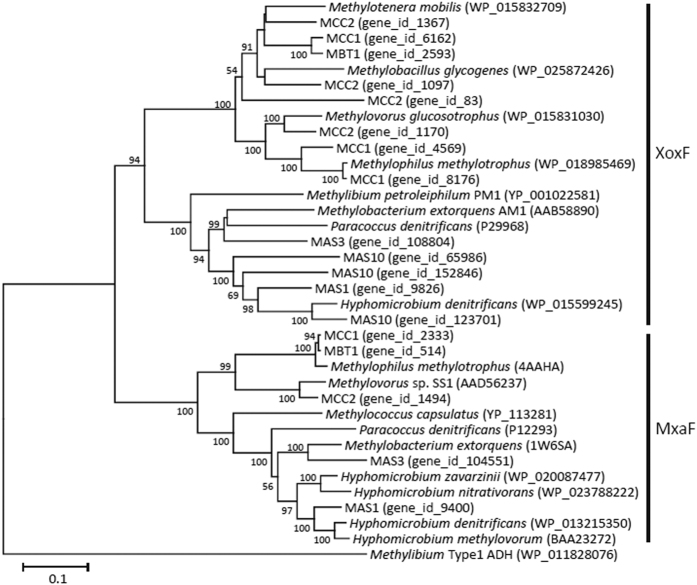
Neighbor-joining tree based on amino-acid sequences of Xox and Mxa methanol dehydrogenases showing phylogenetic relationships among methylotrophs. Bootstrap values (100 trials, only >50 are shown) are indicated at branching points. The bar indicates 10% sequence divergence. Accession numbers are shown in parentheses.

Phylogenetic relationships among the bin genomes representing methylotrophs were analyzed using sequences encoding MxaF, the catalytic subunit of MxaFI-type methanol dehydrogenase, since previous studies have successfully used MxaF for the phylogenetic identification of methylotrophs^22^. Genes encoding 16S rRNA were not found in the bin genomes, since it is difficult to assemble contigs containing highly conserved repeated sequences, such as 16S rRNA genes^21^. Figure 3 shows phylogenetic relationships among the bin genomes and representative methylotroph isolates based on amino-acid sequences of MxaF. This figure also includes XoxF sequences that have recently been discovered as alternative methanol dehydrogenases widely present in the natural environment^23^. Based on the comparative analyses of MxaF sequences, MCC1 and MBC1 are affiliated with the genus *Methylophilus*, MCC2 is closely related to *Methlylovorus*, while MAS1 is confirmed to be a member of the genus *Hyphomicrobium*. These results are supported by the *gyrB*-based phylogenetic analysis (Supplementary Fig. S2) which has been demonstrated useful for fine phylogenetic comparisons among bacterial strains^24^. We also performed the digital DNA-DNA hybridization (DDH) analysis^25^ to examine genome relatedness between the bin genomes and their relatives (Supplementary Table S2). This analysis suggests the possibility that MAS1 represents novel species in the genus *Hyphomicrobium*. Based on the phylogenetic features and relative abundances, it is concluded that bin genomes MAS1, MCC1, MCC2, and MBC1 represent *Hyphomicrobium* in MAS, *Methylophilus* in MCC, *Methylovorus* in MCC, and *Methylophilus* in MBC, respectively, detected by the PCR-based phylogenetic analyses as the major populations (Fig. 1).

The results of the PCR-based phylogenetic analysis and the metagenomic analysis are consistent, and we are therefore able to identify that the *Hyphomicrobium* bacterium represented by bin-genome MAS1 is the dominant methylotroph in the MAS microbiome. Interestingly, however, this bacterium was not abundantly detected in the MCB and MBB microbiomes, and alternative methylotrophs affiliated with the genus *Methylophilus* occurred abundantly. This result demonstrates that methylotrophs isolated after laboratory enrichment cultures do not represent those abundantly present in activated sludge. Since doubling times for microbial growth (Table 1) largely differ between the activated-sludge and laboratory-enrichment systems, we assume that the *Methylophilus* methylotrophs are selected in terms of their abilities for efficient methanol utilization and/or rapid growth in the respective culture systems. However, other features must be necessary for bacteria to become abundant in activated-sludge systems, in which microbes are sustained longer times by forming flocs.

### Genomic features of the dominant methylotroph

We were interested in characterizing genomic features of MAS1 to gain insights into how it abundantly occurs in methanol-fed activated sludge. To this end, we comparatively analyzed bin genomes MAS1, MCC1 and MBC1 for identifying the presence and completeness of functional modules (metabolic pathways) defined in the Kyoto Encyclopedia of Genes and Genomes (KEGG) database^26^ using the MAPLE tool^27^ (Supplementary Table S3). For comparison, we also analyzed functional modules present in genomes of isolated methylotrophs phylogenetically related to the bin-genome organisms (Supplementary Table S3). We found that the *Hyphomicrobium* genomes (including MAS1) share most functional modules, some of which are not coded in the genomes of the *Methylophilus* relatives (including MCC1 and MBC 1); these include modules for crassulacean acid metabolism, assimilatory sulfate reduction, ethylmalonyl pathway, beta oxidation, phosphatidylcholine biosynthesis, and isoprenoid biosynthesis (Supplementary Table S3). On the other hand, some modules are found only in the *Methylophilus*-related genomes, including those for formaldehyde assimilation, pentose-phosphate pathway, lipopolysaccharide metabolism, and sugar metabolism. These results suggest that *Hyphomicrobium* and *Methylophilus* have different C1-assimilation pathways^2^.

We summarize C1-assimilation pathways to comparatively show the presence of relevant genes in the bin genomes MAS1, MCC1 and MBC1 (Supplementary Fig. S3). This figure shows that MAS1 utilizes the serine pathway in combination with the ethylmalonyl-CoA (EMC) pathway, whereas the other organisms use the ribulose monophosphate (RuBP) cycle (Table 3). A difference in these pathways is that carbon is solely derived from methanol via formaldehyde in the RuBP cycle, while carbon from CO_2_ is also incorporated along with that from formaldehyde in the serine pathway. It is likely that the serine pathway is favorable for bacteria thriving in organic carbon-limited ecosystems^6^.

Substantial differences are also found in their lipopolysaccharide-biosynthesis pathways (Supplementary Table S3 and Fig. S4). Genome sequences reveal that *Hyphomicrobium* strains, including MAS1, *H. denitrificans* ATCC51888^28^ and *H. denitrificans* 1NES1^29^, do not have the pathway for biosynthesis of ADP-L-glycero-beta-D-manno-heptose, a precursor of the core-oligosaccharide domain in lipopolysaccharides of gram-negative bacetria^30^, while these organisms are able to synthesize lauroyl-KDO2-lipid constituting the lipid-A domain^31^. This genomic characteristic suggests that the *Hyphomicrobium* bacteria lack core oligosaccharides and linked O-antigens. Since O-antigens are known to be composed of hydrophilic oligosaccharides^30^, we deduce that the differences in lipopolysaccharide-biosynthesis pathways in *Hyphomicrobium* and *Methylophylus* result in their different flocculation properties. Previous studies have shown that *Hyphomicrobium* bacteria are characteristic in their abilities to attach to solid surfaces^32^. In addition, *Hyphomicrobium* cells are known to attach to each other to form rosette-like aggregates^32^. Another study has demonstrated that cell-surface hydrophobicity is important for their attachment and aggregation^33^. It is therefore likely that MAS1 uses hydrophobic cell surfaces for floc formation and persistence in activated sludge. The different appearances of the microbiomes (flocculated vs. planktonic, Table 1) may have been attributed to the differences in cell-surface properties of the major bacteria.

**Figure 4:**
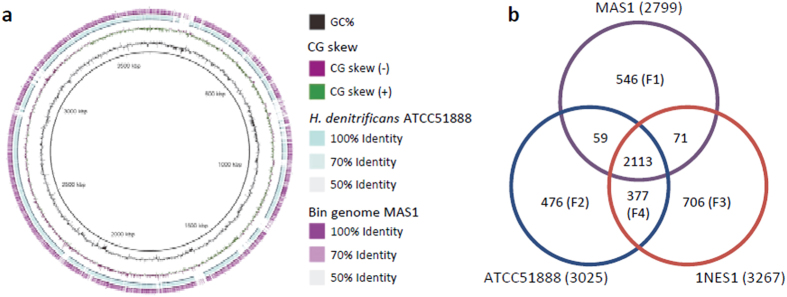
Comparative genomics of bin genome MAS1 and *H. denitrificans* strains ATCC51888 and 1NES1. (**a**) Overall comparison of genomes of MAS1 and ATCC51888 using BRIG. (**b**) A Venn diagram showing peculiar and shared CDSs coded in these genomes. Numbers of CDSs subjected to the analysis are indicated in parentheses after the organism. Refer to supplementary data uploaded in the website for CDS contents.

In order to further characterize bin genome MAS1, genomic features of MAS1 are compared with those of its closest relatives (*H. denitrificans* strains ATCC51888^28^ and 1NES1^29^). We found that the genome size of MAS1 is relatively small (90% or less) compared to those of the two *H. denitrificans* isolates (Table 4). In addition, a number of CDSs in MAS1 is also lower than those of the other strains (Table 4). We next compared coded functions in MAS1 and those in the *H. denitrificans* genomes by the bidirectional best-hit (BBH)^34^ analysis (Fig. 4). This analysis was expected to provide us with information as to peculiar and missing functions in MAS1 in comparison to the other *H. denitrificans* genomes. Prior to this analysis, we evaluated overall genomic similarity between MAS1 and ATCC51888 using BLAST Ring Image Generator (BRIG)^35^ to address if the BBH analysis could provide with meaningful outcomes (Fig. 4a). It was found that these genomes are substantially similar to each other, suggesting that the BBH analysis is useful.

The three genomes were subjected to the BBH analysis, and shared and peculiar CDSs were identified (Fig. 4b). Duplicate genes were not included in this analysis. Peculiar CDSs coded only in the MAS1 bin genome (region F1) are listed in Supplementary Table S4, while CDSs categorized in regions F2, F3 and F4 are listed in Supplementary Tables S5, S6 and S7, respectively. Although many listed CDSs are hypothetical, the lists also provide us with valuable information regarding peculiar and missing functions in MAS1. We found that many CDSs found only in MAS1 (region F1) are related to polysaccharide modification and transporters, while missing functions (CDSs in region F4 that includes those present in the two strains but absent from MAS1) include nitrate reductase, nitric-oxide reductase, *cbb*_3_-type cytochrome *c* oxidase, K^+^-transporting ATPase, and sarcosine oxidase. It is likely that unnecessary (e.g., nitrate reductase) and duplicate (e.g., *cbb*_3_-type oxidase) functions have been lost from the MAS1 genome, while this organism has acquired functions (e.g., polysaccharide modification) that are important for the survival in activated sludge. For instance, MAS1 encodes *aa*_3_-type cytochrome *c* oxidase, while *cbb*_3_-type is lost. This evolutionary consequence is reasonable for organisms thriving in oxygen-rich activated sludge, since *aa*_3_-type oxidase (high proton-pump activity, but low affinity for oxygen) is more favorable in activated sludge than *cbb*_3_-type oxidase (high affinity for oxygen, but low proton-pump activity)^36^.

## Conclusions

One of fundamental questions in microbial ecology is how particular species are selected from diverse microbial species and predominate in an ecosystem. In the present study, metagenomics are used in combination with molecular phylogeny to identify the uncultured dominant methylotroph in activated sludge, and comparative genomics are used to discuss how it predominates there. We suggest that the dominant methylotroph is a member of the genus *Hyphomicrobium* and has a relatively small genome from which unnecessary and duplicate functions are lost. It is likely that the small genome can save the energy for reproduction, resulting in efficient growth in a microbiome. We also suggest the possibility that it has an appropriate repertoire of CDSs relevant to lipopolysaccharide modification, resulting in efficient floc formation and survival in activated sludge. In future studies, functions and expression of these CDSs will be analyzed in detail to understand how lipopolysaccharides contribute to bacterial persistence in activated sludge. Genome sequences provided in the present study are valuable to deepen our understanding of bacterial strategies to adapt to and overgrow in activated-sludge microbiomes.

## Methods

### Enrichment of methylotrophs

SAS was obtained from the Asakawa Water Reclamation Center in Tokyo, Japan. A laboratory activated-sludge reactor was composed of an aeration tank (2 L) and a settling tank (1 L), and its operation was initiated by inoculating the aeration thank with 1 L of SAS. The reactor was continuously suppled with methanol synthetic wastewater (MSW), containing (per L) 0.50 g methanol, 0.12 g NH_4_Cl, 1.9 g NaH_2_PO_4_·2H_2_O, 1.1 g Na_2_HPO_4_, 22 mg CaCl_2_·2H_2_O, 0.43 mg MgSO_4_·7H_2_O, 21 mg KCl, 8.8 mg NaHCO_3_, and 1 mL trace metal solution (DSMZ medium 318, Deutsche Sammlung von Mikroorganismen und Zellkulturen GmbH, Germany), at a hydraulic retention time of 24 h. Air was supplied to the aeration tank at 1 L min^−1^. The mixed-liquor suspended solid concentration was kept at approximately 2,000 mg L^−1^, and the temperature was 30 °C. Sludge-retention time was estimated to be approximately 10 days. For the enrichment in a continuous-culture system, a jar fermenter (2 L in working volume) was inoculated with 330 ml of SAS and continuously suppled with MSW at a flow rate of 2 L day^−1^. The fermenter was agitated at 100 rotation min^−1^ and suppled with air at 1 L min^−1^. For the enrichment in a batch-culture system, 5 mL of MSW in a test tube (25 mL in capacity) was inoculated with 0.1 ml of SAS, and cells were grown by shaking the tube at 100 rpm. After the culture reached the stationary phase, 0.1 ml of the culture was transferred to fresh MSW. The batch culture was repeated five times. Methanol was measured as described elsewhere^37^.

### PCR-based phylogenetic analyses

DNA was extracted from the microbiomes using the FAST DNA Spin Kit for Soil (Q-Bio, Carlsbad, CA, USA). PCR amplification of 16S rRNA gene fragments (V1–V3 region) was performed using primers ad-tag-8F and ad-533R, which contain adaptors for pyrosequencing and an arbitrary tag sequence for sample identification^38^. This region was selected, since previous studies have recommended several specific regions (including the V1 to V3) of 16S rRNA genes for molecular phylogenetic analyses of bacterial populations^39^. PCR conditions were described elsewhere^38^. Amplicons were purified using a QIAquick PCR purification kit (Qiagen, Tokyo, Japan) and subjected to pyrosequencing using a Genome Sequencer FLX system. Twenty- to forty-thousand reads were obtained for each sample, and phylogenetic analyses were conducted using the Silva rRNA database (http://www.arb-silva.de/). Alignment of sequences and construction of neighbor-joining trees were conducted using the MEGA program ver. 5.1^40^.

### Shotgun sequencing and bin-genome reconstruction

After quality check^37^, DNA samples extracted from the four microbiomes were used to construct paired-end and fragmented libraries and sequenced using the HiSeq 2000 sequencing system (Illumina, San Diego, CA) as described elsewhere^37^. One lane was used for each sample, and over 40 Gb raw sequences were obtained for each. Sequence quality check was performed using FastQC (http://www.bioinformatics.babraham.ac.uk/projects/fastqc). Qualified reads were trimmed and assembled into contigs using CLC Genomics Workbench version 6.5.1 (CLC Bio Japan, Tokyo, Japan). Coding sequences in contigs were predicted using MetaGeneMark (http://exon.gatech.edu/meta_gmhmmp.cgi). Gene identification and annotation were performed by BLAST search^41^ (E-value, 10^−3^) against the NCBI nr database and search tools in the KEGG database^26^. Contig selection and bin-genome reconstruction were carried out according to methods described previously^10^,^21^ and using the MaxBin software^42^. ORFs coding for methanol dehydrogenases were searched using the BLAST program^41^ and HMMER web server^43^.

### Comparative genomics

Digital DDH analyses were conducted using genome-to-genome distance calculator^25^. Functional-module analysis was conducted using the MAPLE website (http://www.genome.jp/tools/maple/)^27^. Overall comparison of multiple genomes was conducted using BRIG (http://brig.sourceforge.net/)^35^. Peculiar and shared CDSs in different genomes were extracted by surveying bi-directional best hit relationships across diverse species^34^ using BLSATP with an E-value threshold of 1 × 10^−5^. Redundant functional genes are excluded from the analysis.

## Additional Information

**Accession codes:** Nucleotide sequences determined in the present study have been deposited into the NCBI Sequence Read Archive database under accession number: DRA004030.

**How to cite this article**: Fujinawa, K. *et al*. Genomic features of uncultured methylotrophs in activated-sludge microbiomes grown under different enrichment procedures. *Sci. Rep.* **6**, 26650; doi: 10.1038/srep26650 (2016).

## Supplementary Material

Supplementary Information

## Figures and Tables

**Table 1:** Microbiomes analyzed in the present study.

**Microbiome**	**Culture system**	**Substrate**	**Doubling time (day)**	**Biomass concentration (mg L**^**−1**^)	**Appearance**
SAS	Activated sludge	Sewage	Approx. 10^a^	Approx. 2000^a^	Flocculated
MAS	Activated sludge	Methanol	10	1650 ± 210^b^	Flocculated
MCC	Continuous culture	Methanol	1	132 ± 15.2^b^	Planktonic
MBC	Batch culture	Methanol	0.2	420 ± 0^b^	Planktonic

^a^Data were obtained from operators of the water reclamation center.

^b^Data were obtained by a standard procedure^41^ and are means ± SE.

**Table 2:** Summary of metagenome-sequencing data.

**Metagenome**	**No. of read (×10**^**6**^)	**Total read length (Mbp)**	**No. of contig (×10**^**6**^)	**Average contig length (bp)**	**Total contig length (Mb)**
SAS	323	32300	2.0	693	1635
MAS	427	42700	1.3	1086	1003
MCC	465	46500	0.5	1240	609
MBC	454	45400	0.2	914	142

**Table 3:** Methylotroph bin genomes reconstructed from the MAS, MCC and MBC metagenomes.

**Bin ID**	**Taxon**^a^	**Total Length (bp)**	**No. of Contig**	**G+C content (%)**	**No. of CDS**	**Completeness**^b^ **(%)**	**Predicted genome size**^c^ **(Mb)**	**Relative abundance**^d^ **(%)**	**Methanol dehydrogenase**	**C1-assimilation pathway**^e^
MAS metagenome										
MAS1	AP	3,302,713	20	63.1	3154	98	3.37	9.9	Mxa, Xox	Ser, EMC
MAS3	AP	3,551,003	256	69.6	3576	85	4.12	2.7	Mxa, Xox	Ser, EMC
MAS10	AP	3,189,957	46	61.0	3100	93	3.43	0.7	Xox	EMC
MCC metagenome										
MCC1	BP	2,883,687	19	48.9	2772	87	3.31	17.4	Mxa, Xox	RuMP
MCC2	BP	2,278,161	12	51.6	2159	89	2.56	16.8	Mxa, Xox	RuMP
MBC metagenome										
MBC1	BP	2,865,640	56	51.1	2762	98	2.92	86.0	Mxa, Xox	RuMP

^a^AP, Alphaproteobacteria; BP, Betaproteobacteria.

^b^Estimated based on the frequency of universal single-copy genes (105 genes) in each bin-genome.

^c^Estimated based on the total length and completeness.

^d^Estimated based on numbers of assigned reads and total reads.

^e^EMC, ethylmalonyl-CoA pathway; Ser, serine pathway; RuMP, ribulose-monophosphate cycle.

**Table 4:** Genomic features of MAS1 and two stains (ATCC51888 and 1NES1) affiliated with *H. denitrificans*.

**Genome**	**Size (Mb)**	**No. of CDS**	**G+C content (%)**	**Non-coding DNA (%)**	**Methanol dehydrogenase**	**C1-assimilation pathway**^a^	**Reference (Accession No.)**
MAS1	3,37	3154	63	13.1	Mxa, Xox	Ser, EMC	This strudy
ATCC51888	3.64	3512	60	12.2	Mxa, Xox	Ser, EMC	26 (CP002083)
1NES1	3.81	3842	60	11.8	Mxa	Ser, EMC	27 (CP005587)

^a^Refer to footnotes of Table 3 for abbreviations.
