# Supplementary material for: Genomic features of uncultured methylotrophs in activated-sludge microbiomes grown under different enrichment procedures
Source: Sci Rep. 2016 May 25;6:26650. doi: 10.1038/srep26650 (PMC4879533; doi:10.1038/srep26650)
Supplement: Supplementary Information [file srep26650-s1.pdf]

Supplementary information

Genomic features of uncultured methylotrophs in activated-sludge microbiomes grown under different enrichment procedures

Kazuki Fujinawa[1,*], Yusuke Asai[1,*], Morio Miyahara[1], Atsushi Kouzuma[1], Takashi Abe[2] & Kazuya Watanabe[1]

[1]School of Life Sciences, Tokyo University of Pharmacy and Life Sciences, Horinouchi, Hachioji, Tokyo, Japan. [2]Graduate School of Science and Technology, Niigata University, Niigata, Japan. *These authors contributed equally to this work. Correspondence and requests for materials should be addressed to K.W. (email: kazuyaw@toyaku.ac.jp)

Table S1. Bin genomes reconstructed from the MAS, MCC and MBC metagenomes.
[a]AP, *Alphaproteobacteria*; BP, *Betaproteobacteria*; BR, *Bacteroidia*; CP, *Cytophagia*; DP, *Deltaproteobacteria*; FB, *Flavobacteria*; PM, *Planctomycetia*.
[b]Estimated based on the frequency of universal single-copy genes (105 genes) in each bin-genome. [c]Estimated based on the total length and completeness.
[d]Estimated based on numbers of assigned reads and total reads. [e]EMC, ethylmalonyl-CoA pathway; Ser, serine pathway; RuMP, ribulose-monophosphate cycle.

Bin ID	Taxon[a]	Total Length (bp)	No. of Contig	G+C content (%)	No. of CDS	Completeness[b] (%)	Predicted genome size[c] (Mb)	Relative abundance[d] (%)	Methanol dehydrogenase	C1-assimilation pathway[e]	Function
MAS metagenome											
MAS1	AP	3,302,713	20	63.1	3154	98	3.37	9.9	Mxa, Xox	Ser, EMC	Aerobic methylotroph
MAS2	BR	3,069,599	247	37.3	2697	90	3.41	3.8			Aerobic heterotroph
MAS3	AP	3,551,003	256	69.6	3576	85	4.12	2.7	Mxa, Xox	Ser, EMC	Aerobic methylotroph
MAS4	BP	6,233,189	827	72.0	6685	79	7.89	4.5			Aerobic heterotroph
MAS5	BP	4,265,174	429	71.7	4233	73	5.84	3.3			Aerobic heterotroph
MAS6	FB	2,539,468	142	31.1	2241	87	2.92	1.2			Aerobic heterotroph
MAS8	AP	3,052,623	71	55.6	2824	89	3.43	1.7			Aerobic heterotroph
MAS9	PM	4,860,909	29	64.3	3912	84	5.78	2.0			Aerobic heterotroph
MAS10	AP	3,189,957	46	61.0	3100	93	3.43	0.7	Xox	EMC	Aerobic methylotroph
MCC metagenome											Aerobic heterotroph
MCC1	BP	2,883,687	19	48.9	2772	87	3.31	17.4	Mxa, Xox	RuMP	Aerobic methylotroph
MCC2	BP	2,278,161	12	51.6	2159	89	2.56	16.8	Mxa, Xox	RuMP	Aerobic methylotroph
MCC3	DP	9,811,799	291	70.2	7804	88	11.15	15.3			Aerobic heterotroph
MCC4	CP	5,714,079	36	51.0	4485	79	7.22	0.7			Aerobic heterotroph
MBC metagenome											Aerobic heterotroph
MBC1	BP	2,865,640	56	51.1	2762	98	2.92	86.0	Mxa, Xox	RuMP	Aerobic methylotroph
MBC2	BP	4,436,393	93	69.6	4121	87	5.10	2.3			Aerobic heterotroph
MBC3	AP	4,474,689	57	66.0	4274	72	6.21	1.0			Aerobic heterotroph

Table S2. Genome relatedness as determined by the Digital DDH method.

Query genome	Reference genome	DDH (%)
MAS1	*Hyphomicrobium_denitrificans*_ATCC_51888	16.8
	*Hyphomicrobium_denitrificans*_1NES1	15.4
MAS10	*Hyphomicrobium_denitrificans*_ATCC_51888	80.2
	*Hyphomicrobium_denitrificans*_1NES1	47.2
MBC1	*Methylophilus_methylotrophus*_ATCC53528	26.8
MCC1	*Methylophilus_methylotrophus*_ATCC53528	30.1

Table S2 The presence and completeness of KEGG functional modules in bin genomes and relative strains [a]

ID	Function	Components	MBC1	MCC1	MAS1	mph	mmb	meh	mei	hdn	hdt	hni	Description
Energy metabolism													
Carbon fixation													
M00165	Reductive pentose phosphate cycle (Calvin cycle)	11	72.7	72.7	90.9	72.7	45.5	72.7	72.7	90.9	90.9	81.8	
M00166	Reductive pentose phosphate cycle, ribulose-5P => glyceraldehyde-3P	4	50	50	75	50	50	50	50	75	75	75	
M00167	Reductive pentose phosphate cycle, glyceraldehyde-3P => ribulose-5P	7	85.7	85.7	100	85.7	42.9	85.7	85.7	100	100	85.7	RuMP pathway
M00168	CAM (Crassulacean acid metabolism), dark	2	50	50	100	50	0	50	50	100	100	100	
M00169	CAM (Crassulacean acid metabolism), light	2	50	50	100	50	50	50	50	50	50	50	
M00172	C4-dicarboxylic acid cycle, NADP - malic enzyme type	4	50	50	75	50	25	50	50	50	50	50	
M00171	C4-dicarboxylic acid cycle, NAD - malic enzyme type	7	14.3	14.3	28.6	14.3	0	14.3	14.3	28.6	28.6	28.6	
M00170	C4-dicarboxylic acid cycle, phosphoenolpyruvate carboxykinase type	4	25	25	50	25	0	25	25	50	50	25	
M00173	Reductive citrate cycle (Arnon-Buchanan cycle)	10..12	63.6	63.6	72.7	63.6	60	63.6	54.5	72.7	72.7	72.7	
M00376	3-Hydroxypropionate bi-cycle	13	23.1	23.1	53.8	23.1	23.1	23.1	7.7	53.8	53.8	53.8	
M00375	Hydroxypropionate-hydroxybutylate cycle	14	0	0	14.3	0	0	0	0	14.3	14.3	14.3	
M00374	Dicarboxylate-hydroxybutyrate cycle	13	38.5	38.5	46.2	38.5	30.8	38.5	30.8	46.2	46.2	46.2	
M00377	Reductive acetyl-CoA pathway (Wood-Ljungdahl pathway)	7	42.9	42.9	42.9	42.9	28.6	42.9	42.9	42.9	42.9	42.9	
M00579	Phosphate acetyltransferase-acetate kinase pathway, acetyl-CoA => acetate	2	0	50	0	50	0	0	0	100	100	0	
Nitrogen metabolism													
M00175	Nitrogen fixation, nitrogen => ammonia	1	0	0	0	0	0	0	0	0	0	0	
M00531	Assimilatory nitrate reduction, nitrate => ammonia	2	50	0	50	50	50	50	50	50	50	100	
M00530	Dissimilatory nitrate reduction, nitrate => ammonia	2	50	50	50	50	50	50	50	100	100	50	
M00529	Denitrification, nitrate => nitrogen	4	0	0	0	0	50	0	0	100	100	100	
M00528	Nitrification, ammonia => nitrite	2	0	0	0	0	0	0	0	0	0	0	
Methane metabolism													
M00567	Methanogenesis, CO2 => methane	8	25	25	25	25	25	25	25	37.5	25	25	
M00357	Methanogenesis, acetate => methane	5..6	20	20	20	20	20	20	20	50	33.3	20	
M00356	Methanogenesis, methanol => methane	3	0	0	0	0	0	0	0	33.3	0	0	
M00563	Methanogenesis, methylamine/dimethylamine/trimethylamine => methane	4	0	0	0	0	0	0	0	25	0	0	
M00358	Coenzyme M biosynthesis	4	0	0	0	0	0	0	0	0	0	0	
M00174	Methane oxidation, methanotroph, methane => formaldehyde	2	50	50	50	50	0	0	50	50	0	50	
M00346	Formaldehyde assimilation, serine pathway	9	33.3	33.3	77.8	33.3	22.2	33.3	33.3	77.8	66.7	66.7	
M00345	Formaldehyde assimilation, ribulose monophosphate pathway	3..4	75	75	0	75	50	75	75	0	0	0	
M00344	Formaldehyde assimilation, xylulose monophosphate pathway	4	50	50	25	50	0	50	50	25	25	25	
M00378	F420 biosynthesis	5	0	0	0	0	0	0	0	0	0	0	
M00422	Acetyl-CoA pathway, CO2 => acetyl-CoA	2	0	0	0	0	0	0	0	0	0	0	
Sulfur metabolism													
M00176	Assimilatory sulfate reduction, sulfate => H2S	3	66.7	66.7	100	33.3	33.3	33.3	33.3	100	100	100	
M00596	Dissimilatory sulfate reduction, sulfate => H2S	3	33.3	33.3	0	33.3	33.3	33.3	33.3	0	0	0	
M00595	Thiosulfate oxidation by SOX complex, thiosulfate => sulfate	1	0	0	0	0	0	0	0	100	0	0	

[a]mph, Methylophilus sp. TWE2; mmb, *Methylotenera mobilis* ; meh, *Methylotenera versatilis* ; mei, *Methylovorus glucosetrophus* ; hdn, *Hyphomicrobium denitrificans* ATCC 51888; hdt, Hyphomicrobium denitrificans 1NES1; hni, Hyphomicrobium nitrativorans. A percentage to the total compoments is shown for each organism.

Table S2 Continued

ID	Function	Components	MBC1	MCC1	MAS1	mfa	mmb	meh	mei	hdn	hdt	hni	Descrition
Carbohydrate and lipid metabolism													
Central carbohydrate metabolism													
M00001	Glycolysis (Embden-Meyerhof pathway), glucose => pyruvate	9..10	80	80	90	80	80	90	90	90	90	80	
M00002	Glycolysis, core module involving three-carbon compounds	5..6	83.3	83.3	100	83.3	100	100	100	100	100	100	
M00003	Gluconeogenesis, oxaloacetate => fructose-6P	7..8	87.5	87.5	100	87.5	71.4	87.5	87.5	100	100	100	
M00307	Pyruvate oxidation, pyruvate => acetyl-CoA	1	100	100	100	100	100	100	100	100	100	100	
M00009	Citrate cycle (TCA cycle, Krebs cycle)	8	87.5	87.5	100	87.5	87.5	87.5	75	100	100	87.5	
M00010	Citrate cycle, first carbon oxidation, oxaloacetate => 2-oxoglutarate	3	100	100	100	100	100	100	100	100	100	100	
M00011	Citrate cycle, second carbon oxidation, 2-oxoglutarate => oxaloacetate	5	80	80	100	80	80	80	60	100	100	80	
M00004	Pentose phosphate pathway (Pentose phosphate cycle)	7..8	100	100	71.4	100	100	100	100	71.4	100	71.4	
M00006	Pentose phosphate pathway, oxidative phase, glucose 6P => ribulose 5P	2..3	100	100	0	100	100	100	100	0	100	0	
M00007	Pentose phosphate pathway, non-oxidative phase, fructose 6P => ribose 5P	4	100	100	100	100	100	100	100	100	100	100	
M00580	Pentose phosphate pathway, archaea, fructose 6P => ribose 5P	2..3	100	100	50	100	100	100	100	50	50	50	
M00005	PRPP biosynthesis, ribose 5P => PRPP	1	100	100	100	100	100	100	100	100	100	100	
M00008	Entner-Doudoroff pathway, glucose-6P => glyceraldehyde-3P + pyruvate	4	100	100	0	100	100	100	100	0	50	0	
M00308	Semi-phosphorylative Entner-Doudoroff pathway, gluconate => glycerate-3P	4..5	60	60	40	60	60	60	60	40	40	40	
M00633	Semi-phosphorylative Entner-Doudoroff pathway, gluconate/galactonate => glycerate-3P	4	0	0	0	0	0	0	0	0	0	0	
M00309	Non-phosphorylative Entner-Doudoroff pathway, gluconate/galactonate => glycerate	3	0	0	0	0	0	0	0	0	0	0	
Other carbohydrate metabolism													
M00012	Glyoxylate cycle	5	40	40	60	40	40	40	40	80	60	80	
M00373	Ethylmalonyl pathway	14	7.1	7.1	92.9	7.1	7.1	7.1	0	92.9	92.9	92.9	EMCP pathway
M00532	Photorespiration	10	40	40	60	40	40	40	40	60	60	60	
M00013	Malonate semialdehyde pathway, propanoyl-CoA => Acetyl-CoA	4	0	25	0	0	0	25	0	0	0	25	
M00632	Galactose degradation, Leloir pathway, galactose => alpha-D-glucose-1P	4	25	25	25	0	25	25	25	25	25	25	
M00552	D-galactonate degradation, De Ley-Doudoroff pathway, D-galactonate => glycerate-3P	5	40	40	40	40	40	40	40	40	40	40	
M00014	Glucuronate pathway (uronate pathway)	8	12.5	12.5	12.5	0	12.5	0	12.5	12.5	12.5	12.5	
M00630	D-Galacturonate degradation (fungi), D-galacturonate => glycerol	4	0	0	0	0	0	0	0	0	0	0	
M00631	D-Galacturonate degradation (bacteria), D-galacturonate => pyruvate + D-glyceraldehyde 3P	5	20	20	0	20	20	20	20	0	0	0	
M00061	D-Glucuronate degradation, D-glucuronate => pyruvate + D-glyceraldehyde 3P	5	20	20	0	20	20	20	20	0	0	0	
M00081	Pectin degradation	3	0	0	0	0	0	0	0	0	0	0	
Fatty acid metabolism													
M00082	Fatty acid biosynthesis, initiation	2..3	100	100	100	100	100	100	100	100	100	100	
M00083	Fatty acid biosynthesis, elongation	1..4	100	100	100	100	100	100	100	100	100	100	
M00085	Fatty acid biosynthesis, elongation, mitochondria	3..4	0	0	0	0	0	0	0	0	0	0	
M00415	Fatty acid biosynthesis, elongation, endoplasmic reticulum	4	0	0	0	0	0	0	0	0	0	0	
M00113	Jasmonic acid biosynthesis	8	0	0	0	0	0	0	0	0	0	0	
M00086	beta-Oxidation, acyl-CoA synthesis	1	0	0	100	0	0	0	0	100	100	100	
M00087	beta-Oxidation	3..4	0	33.3	66.7	0	0	0	0	66.7	66.7	66.7	
M00088	Ketone body biosynthesis, acetyl-CoA => acetoacetate/3-hydroxybutyrate/acetone	5	0	0	60	0	0	0	0	60	60	20	

Table S2 Continued

ID	Function	Components	MBC1	MCC1	MAS1	mfa	mmb	meh	mei	hdn	hdt	hni	Descrition
Lipid metabolism													
M00089	Triacylglycerol biosynthesis	4	50	50	50	50	50	50	50	50	50	50	
M00098	Acylglycerol degradation	2	0	0	0	0	0	0	0	0	0	0	
M00090	Phosphatidylcholine (PC) biosynthesis, choline => PC	3	0	0	0	0	33.3	0	0	0	0	0	
M00091	Phosphatidylcholine (PC) biosynthesis, PE => PC	1..2	0	0	100	0	0	0	0	100	100	100	
M00092	Phosphatidylethanolamine (PE) biosynthesis, ethanolamine => PE	3	0	0	0	0	0	0	0	0	0	0	
M00093	Phosphatidylethanolamine (PE) biosynthesis, PA => PS => PE	3	100	100	100	100	100	100	100	100	100	100	
M00130	Inositol phosphate metabolism, PI=> PIP2 => Ins(1,4,5)P3 => Ins(1,3,4,5)P4	4	0	0	0	0	0	0	0	0	0	0	
M00131	Inositol phosphate metabolism, Ins(1,3,4,5)P4 => Ins(1,3,4)P3 => myo-inositol	4	25	25	25	25	25	25	25	25	25	25	
M00132	Inositol phosphate metabolism, Ins(1,3,4)P3 => phytate	3	0	0	0	0	0	0	0	0	0	0	
M00094	Ceramide biosynthesis	4	0	0	0	0	0	0	0	0	0	0	
M00066	Lactosylceramide biosynthesis	2	0	0	0	0	0	0	0	50	50	0	
M00067	Cerebroside and sulfatide biosynthesis	2	0	0	0	0	0	0	0	0	0	0	
M00099	Sphingosine biosynthesis	5	0	0	0	0	0	0	0	0	0	0	
M00100	Sphingosine degradation	2	0	0	0	0	0	0	0	0	0	0	
Lipopolysaccharide metabolism													
M00060	Lipopolysaccharide biosynthesis, KDO2-lipid A	9	88.9	88.9	77.8	88.9	88.9	88.9	88.9	77.8	77.8	77.8	
M00063	CMP-KDO biosynthesis	3	66.7	66.7	66.7	66.7	100	100	100	66.7	66.7	66.7	
M00064	ADP-L-glycero-D-manno-heptose biosynthesis	4	100	100	0	100	100	100	100	0	0	0	
M00080	Lipopolysaccharide biosynthesis, inner core => outer core => O-antigen	12	41.7	41.7	8.3	33.3	41.7	41.7	41.7	8.3	8.3	8.3	
Sugar metabolism													
M00549	Nucleotide sugar biosynthesis, glucose => UDP-glucose	3	100	100	66.7	100	100	100	100	66.7	66.7	33.3	
M00554	Nucleotide sugar biosynthesis, galactose => UDP-galactose	2	0	0	0	0	0	0	0	0	0	0	
M00565	Trehalose biosynthesis, D-glucose 1P => trehalose	5	80	80	0	100	80	80	100	0	0	0	
Glycan metabolism													
M00055	N-glycan precursor biosynthesis	13	0	0	0	0	0	0	0	0	0	0	
M00073	N-glycan precursor trimming	3	0	0	0	0	0	0	0	0	0	0	
M00074	N-glycan biosynthesis, high-mannose type	6	0	0	0	0	0	0	0	0	0	0	
M00075	N-glycan biosynthesis, complex type	9	0	0	11.1	0	0	0	0	0	0	0	
M00056	O-glycan biosynthesis, mucin type core	4	0	0	0	0	0	0	0	0	0	0	
M00070	Glycosphingolipid biosynthesis, lacto-series, LacCer => Lc4Cer	2	0	0	0	0	0	0	0	0	0	0	
M00071	Glycosphingolipid biosynthesis, neolacto-series, LacCer => nLc4Cer	2	0	0	0	0	0	0	0	0	0	0	
M00068	Glycosphingolipid biosynthesis, globo-series, LacCer => Gb4Cer	2	0	0	0	0	0	0	0	0	0	0	
M00069	Glycosphingolipid biosynthesis, ganglio series, LacCer => GT3	2	0	0	0	0	0	0	0	0	0	0	
M00065	GPI-anchor biosynthesis, core oligosaccharide	8	0	0	0	0	0	0	0	0	0	0	

Table S2 Continued

ID	Function	Components	MBC1	MCC1	MAS1	mfa	mmb	meh	mei	hdn	hdt	hni	Descrition
Glycosaminoglycan metabolism													
M00057	Glycosaminoglycan biosynthesis, linkage tetrasaccharide	4	0	0	0	0	0	0	0	0	0	0	
M00058	Glycosaminoglycan biosynthesis, chondroitin sulfate backbone	2	0	0	0	0	0	0	0	0	0	0	
M00059	Glycosaminoglycan biosynthesis, heparan sulfate backbone	5	0	0	0	0	0	0	0	0	0	0	
M00076	Dermatan sulfate degradation	6	0	0	0	0	0	0	0	0	0	0	
M00077	Chondroitin sulfate degradation	4	0	0	0	0	0	0	0	0	0	0	
M00078	Heparan sulfate degradation	8	0	0	0	0	0	0	0	0	0	0	
M00079	Keratan sulfate degradation	5	0	0	0	0	0	0	0	0	0	0	
Terpenoid backbone biosynthesis													
M00095	C5 isoprenoid biosynthesis, mevalonate pathway	7	0	0	28.6	0	0	0	0	28.6	28.6	28.6	
M00096	C5 isoprenoid biosynthesis, non-mevalonate pathway	8	87.5	87.5	87.5	87.5	87.5	87.5	87.5	100	100	87.5	
M00364	C10-C20 isoprenoid biosynthesis, bacteria	2	50	50	100	50	50	50	50	100	100	100	
M00365	C10-C20 isoprenoid biosynthesis, archaea	2	0	0	50	0	0	0	0	50	50	50	
M00366	C10-C20 isoprenoid biosynthesis, plants	4	0	0	25	0	0	0	0	25	25	25	
M00367	C10-C20 isoprenoid biosynthesis, non-plant eukaryotes	3	0	0	33.3	0	0	0	0	33.3	33.3	33.3	
Sterol biosynthesis													
M00101	Cholesterol biosynthesis, squalene 2,3-epoxide => cholesterol	10	0	0	0	0	0	0	0	0	0	0	
M00102	Ergocalciferol biosynthesis	5	0	0	0	0	0	0	0	0	0	0	
M00103	Cholecalciferol biosynthesis	2	0	0	0	0	0	0	0	0	0	0	
M00104	Bile acid biosynthesis, cholesterol => cholate/chenodeoxycholate	12	0	0	0	0	0	0	0	0	0	0	
M00106	Conjugated bile acid biosynthesis, cholate => taurocholate/glycocholate	2	0	0	0	0	0	0	0	0	0	0	
M00107	Steroid hormone biosynthesis, cholesterol => prognenolone => progesterone	2	0	0	0	0	0	0	0	0	0	0	
M00108	C21-Steroid hormone biosynthesis, progesterone => corticosterone/aldosterone	3	0	0	0	0	0	0	0	0	0	0	
M00109	C21-Steroid hormone biosynthesis, progesterone => cortisol/cortisone	4	0	0	0	0	0	0	0	0	0	0	
M00110	C19/C18-Steroid hormone biosynthesis, pregnenolone => androstenedione => estrone	3	0	0	0	0	0	0	0	0	0	0	
Other terpenoid biosynthesis													
M00097	beta-Carotene biosynthesis, GGAP => beta-carotene	6	16.7	16.7	16.7	16.7	16.7	16.7	16.7	16.7	16.7	16.7	
M00372	Abscisic acid biosynthesis, beta-carotene => abscisic acid	5	0	0	0	0	0	0	0	0	0	0	
M00371	Castasterone biosynthesis, campesterol => castasterone	5	0	0	0	0	0	0	0	0	0	0	
Nucleotide and amino acid metabolism													
Purine metabolism													
M00048	Inosine monophosphate biosynthesis, PRPP + glutamine => IMP	9..11	100	100	100	100	100	100	100	100	100	100	
M00049	Adenine ribonucleotide biosynthesis, IMP => ADP,ATP	4	100	100	100	100	100	100	100	100	100	100	
M00050	Guanine ribonucleotide biosynthesis IMP => GDP,GTP	4	100	100	100	100	100	100	100	100	100	100	
M00546	Purine degradation, xanthine => urea	5..6	0	0	0	0	0	0	0	0	0	0	

Table S2 Continued

ID	Function	Components	MBC1	MCC1	MAS1	mfa	mmb	meh	mei	hdn	hdt	hni	Description
Pyrimidine metabolism													
M00051	Uridine monophosphate biosynthesis, glutamine (+ PRPP) => UMP	3..6	66.7	83.3	83.3	83.3	83.3	83.3	83.3	83.3	83.3	75	
M00052	Pyrimidine ribonucleotide biosynthesis, UMP => UDP/UTP,CDP/CTP	3	100	100	100	100	100	100	100	100	100	100	
M00053	Pyrimidine deoxyribonuleotide biosynthesis, CDP/CTP => dCDP/dCTP,dTDP/dTTP	8	87.5	87.5	75	87.5	87.5	87.5	87.5	87.5	75	87.5	
M00046	Pyrimidine degradation, uracil => beta-alanine, thymine => 3-aminoisobutanoate	3	0	0	0	0	0	0	0	0	0	0	
Serine and threonine metabolism													
M00020	Serine biosynthesis, glycerate-3P => serine	3	66.7	66.7	100	100	66.7	66.7	66.7	33.3	100	100	Serine pathway
M00018	Threonine biosynthesis, aspartate => homoserine => threonine	5	100	100	100	100	100	100	100	100	100	100	
M00555	Betaine biosynthesis, choline => betaine	1..2	0	0	0	0	0	100	0	0	0	0	
Cysteine and methionine metabolism													
M00021	Cysteine biosynthesis, serine => cysteine	2	100	100	100	100	100	100	100	100	100	100	
M00338	Cysteine biosynthesis, homocysteine + serine => cysteine	2	0	0	0	0	0	0	0	0	0	0	
M00609	Cysteine biosynthesis, methionine => cysteine	6	16.7	16.7	16.7	16.7	16.7	16.7	16.7	16.7	16.7	16.7	
M00017	Methionine biosynthesis, apartate => homoserine => methionine	7	57.1	57.1	71.4	57.1	57.1	57.1	71.4	71.4	71.4	85.7	
M00034	Methionine salvage pathway	8..11	50	50	50	50	50	50	50	50	50	50	
M00035	Methionine degradation	4	75	75	50	50	75	50	75	75	50	75	
M00368	Ethylene biosynthesis, methionine => ethylene	3	33.3	33.3	33.3	33.3	33.3	33.3	33.3	33.3	33.3	33.3	
Branched-chain amino acid metabolism													
M00019	Valine/isoleucine biosynthesis, pyruvate => valine / 2-oxobutanoate => isoleucine	4	100	100	100	100	100	100	100	100	100	100	
M00535	Isoleucine biosynthesis, pyruvate => 2-oxobutanoate	3	66.7	66.7	66.7	66.7	66.7	66.7	66.7	66.7	66.7	66.7	
M00570	Isoleucine biosynthesis, threonine => 2-oxobutanoate => isoleucine	5	100	100	100	100	100	100	100	100	100	100	
M00432	Leucine biosynthesis, 2-oxoisovalerate => 2-oxoisocaproate	3	100	100	100	100	100	100	100	100	100	100	
M00036	Leucine degradation, leucine => acetoacetate + acetyl-CoA	6	16.7	33.3	16.7	16.7	16.7	16.7	16.7	16.7	16.7	16.7	
Lysine metabolism													
M00016	Lysine biosynthesis, succinyl-DAP pathway, aspartate => lysine	9	100	100	100	100	100	100	100	100	100	77.8	
M00525	Lysine biosynthesis, acetyl-DAP pathway, aspartate => lysine	9	66.7	66.7	66.7	66.7	66.7	66.7	66.7	66.7	66.7	55.6	
M00526	Lysine biosynthesis, DAP dehydrogenase pathway, aspartate => lysine	6	83.3	83.3	83.3	83.3	83.3	83.3	83.3	83.3	83.3	66.7	
M00527	Lysine biosynthesis, DAP aminotransferase pathway, aspartate => lysine	7	85.7	85.7	85.7	85.7	85.7	85.7	85.7	85.7	85.7	71.4	
M00030	Lysine biosynthesis, AAA pathway, 2-oxoglutarate => 2-aminoadipate => lysine	8	0	0	12.5	0	0	0	0	12.5	12.5	12.5	
M00433	Lysine biosynthesis, 2-oxoglutarate => 2-oxoadipate	3..4	0	0	0	0	0	0	0	0	0	0	
M00031	Lysine biosynthesis, 2-aminoadipate => lysine	5	0	0	0	0	0	0	0	0	0	0	
M00032	Lysine degradation, lysine => saccharopine => acetoacetyl-CoA	7..9	0	0	50	0	0	0	0	50	50	37.5	
M00608	2-Oxocarboxylic acid chain extension, 2-oxoglutarate => 2-oxoadipate => 2-oxopimelate => 2-oxosuber	3	0	0	0	0	0	0	0	0	0	0	
Arginine and proline metabolism													
M00015	Proline biosynthesis, glutamate => proline	2..3	100	100	100	100	100	100	100	100	100	100	
M00028	Ornithine biosynthesis, glutamate => ornithine	4..5	100	100	100	100	100	100	100	100	100	100	
M00029	Urea cycle	5	40	40	40	40	40	40	40	40	40	40	

Table S2 Continued

ID	Function	Components	MBC1	MCC1	MAS1	mfa	mmb	meh	mei	hdn	hdt	hni	Descrition
Histidine metabolism													
M00026	Histidine biosynthesis, PRPP => histidine	7..9	88.9	87.5	88.9	88.9	88.9	88.9	88.9	88.9	88.9	88.9	
M00045	Histidine degradation, histidine => N-formiminoglutamate => glutamate	4	0	0	0	0	0	25	0	0	0	0	
Aromatic amino acid metabolism													
M00022	Shikimate pathway, phosphoenolpyruvate + erythrose-4P => chorismate	4..7	100	100	100	100	100	100	100	100	100	85.7	
M00023	Tryptophan biosynthesis, chorismate => tryptophan	3..5	100	100	100	100	100	100	100	100	100	100	
M00024	Phenylalanine biosynthesis, chorismate => phenylalanine	2..3	50	50	33.3	50	50	50	50	33.3	33.3	33.3	
M00025	Tyrosine biosynthesis, chorismate => tyrosine	2..3	66.7	66.7	0	66.7	66.7	66.7	66.7	0	0	0	
M00040	Tyrosine biosynthesis, prephanate => pretyrosine => tyrosine	2	0	0	50	0	0	0	0	50	50	50	
M00042	Catecholamine biosynthesis, tyrosine => dopamine => noradrenaline => adrenaline	4	0	0	0	0	0	0	0	0	0	0	
M00043	Thyroid hormone biosynthesis, tyrosine => triiodothyronine/thyroxine	1	0	0	0	0	0	0	0	0	0	0	
M00044	Tyrosine degradation, tyrosine => homogentisate	5	0	0	0	0	0	0	0	0	0	0	
M00533	Homoprotocatechuate degradation, homoprotocatechuate => 2-oxohept-3-enedioate	4	0	0	0	0	0	0	0	0	0	0	
M00545	Trans-cinnamate degradation, trans-cinnamate => acetyl-CoA	6..7	0	0	0	0	0	0	0	0	0	0	
M00350	Capsaicin biosynthesis, L-Phenylalanine => Capsaicin	4	0	0	0	0	0	0	0	0	0	0	
M00037	Melatonin biosynthesis, tryptophan => serotonin => melatonin	4	0	0	0	0	0	0	0	0	0	0	
M00038	Tryptophan metabolism, tryptophan => kynurenine => 2-aminomuconate	7	0	0	0	0	0	0	0	0	0	0	
Other amino acid metabolism													
M00027	GABA (gamma-Aminobutyrate) shunt	3	0	0	33.3	0	0	66.7	0	66.7	66.7	66.7	
M00047	Creatine pathway	3	0	0	0	0	0	0	0	0	0	0	
Cofactor and vitamin biosynthesis													
M00127	Thiamine biosynthesis, AIR => thiamine-P/thiamine-2P	5	80	80	80	80	80	80	80	80	80	60	
M00125	Riboflavin biosynthesis, GTP => riboflavin/FMN/FAD	6..8	100	100	100	100	100	100	100	100	100	83.3	
M00124	Pyridoxal biosynthesis, erythrose-4P => pyridoxal-5P	6	50	50	66.7	50	50	50	50	66.7	66.7	66.7	
M00115	NAD biosynthesis, aspartate => NAD	5	100	100	100	100	100	100	100	100	100	100	
M00622	Nicotinate degradation, nicotinate => fumarate	6	0	0	0	0	0	0	0	0	0	0	
M00119	Pantothenate biosynthesis, valine/L-aspartate => pantothenate	5	80	80	80	80	100	80	80	80	80	80	
M00120	Coenzyme A biosynthesis, pantothenate => CoA	3..5	100	100	100	100	100	100	100	100	100	100	
M00572	Pimeloyl-ACP biosynthesis, BioC-BioH pathway, malonyl-ACP => pimeloyl-ACP	6	100	100	66.7	83.3	100	100	100	66.7	66.7	66.7	
M00123	Biotin biosynthesis, pimeloyl-ACP/CoA => biotin	4	100	100	100	100	100	100	100	100	100	100	
M00573	Biotin biosynthesis, BioI pathway, long-chain-acyl-ACP => pimeloyl-ACP => biotin	5	80	80	80	80	80	80	80	80	80	80	
M00577	Biotin biosynthesis, BioW pathway, pimelate => pimeloyl-CoA => biotin	5	80	80	80	80	80	80	80	80	80	80	
M00126	Tetrahydrofolate biosynthesis, GTP => THF	5..7	100	100	85.7	100	100	100	100	85.7	85.7	71.4	
M00121	Heme biosynthesis, glutamate => protoheme/siroheme	11	90.9	90.9	72.7	90.9	90.9	90.9	90.9	72.7	90.9	63.6	
M00129	Ascorbate biosynthesis, animals, glucose-1P => ascorbate	6	33.3	33.3	33.3	16.7	33.3	16.7	33.3	33.3	33.3	33.3	
M00114	Ascorbate biosynthesis, plants, glucose-6P => ascorbate	7..9	44.4	44.4	44.4	44.4	44.4	22.2	33.3	44.4	44.4	33.3	
M00550	Ascorbate degradation, ascorbate => D-xylulose-5P	5	0	0	0	0	0	0	0	0	0	0	
M00112	Tocopherol/tocotorienol biosynthesis	4	0	0	0	0	0	0	0	0	0	0	
M00122	Cobalamin biosynthesis, cobinamide => cobalamin	7	100	100	85.7	100	100	100	100	85.7	85.7	100	

Table S2 Continued

ID	Function	Components	MBC1	MCC1	MAS1	mfa	mmb	meh	mei	hdn	hdt	hni	Descrition
Cofactor and vitamin biosynthesis													
M00117	Ubiquinone biosynthesis, prokaryotes, chorismate => ubiquinone	9	66.7	66.7	55.6	77.8	66.7	66.7	66.7	66.7	66.7	66.7	
M00128	Ubiquinone biosynthesis, eukaryotes, 4-hydroxybenzoate => ubiquinone	6	16.7	16.7	0	0	16.7	16.7	16.7	0	0	0	
M00116	Menaquinone biosynthesis, chorismate => menaquinone	9	11.1	11.1	11.1	11.1	11.1	11.1	11.1	11.1	11.1	11.1	
M00118	Glutathione biosynthesis, glutamate => glutathione	2	100	100	100	100	100	100	100	100	100	100	
M00140	C1-unit interconversion, prokaryotes	3	100	100	100	100	66.7	100	100	100	100	100	
M00141	C1-unit interconversion, eukaryotes	2..3	50	50	50	50	50	50	50	50	50	50	
Polyamine biosynthesis													
M00133	Polyamine biosynthesis, arginine => agmatine => putrescine => spermidine	4	50	50	25	50	50	75	50	25	25	25	
M00134	Polyamine biosynthesis, arginine => ornithine => putrescine	2	0	0	50	50	0	0	0	50	50	50	
M00135	GABA biosynthesis, eukaryotes, putrescine => GABA	3	0	0	0	33.3	0	0	0	0	0	33.3	
M00136	GABA biosynthesis, prokaryotes, putrescine => GABA	4	0	0	25	0	0	25	0	25	0	0	
Phenylpropanoid and flavonoid biosynthesis													
M00039	Monolignol biosynthesis, phenylalanine/tyrosine => monolignol	10	0	0	0	0	0	0	0	0	0	0	
M00137	Flavanone biosynthesis, phenylalanine => naringenin	5	0	0	0	0	0	0	0	0	0	0	
M00138	Flavonoid biosynthesis, naringenin => pelargonidin	3	0	0	0	0	0	0	0	0	0	0	
Alkaloid and other secondary metabolite biosynthesis													
M00369	Cyanogenic glycoside biosynthesis, tyrosine => dhurrin	3	0	0	0	0	0	0	0	0	0	0	
M00370	Glucosinolate biosynthesis, tryptophan => glucobrassicin	5	0	0	0	0	0	0	0	0	0	0	
M00033	Ectoine biosynthesis, aspartate => ectoine	5	40	40	40	40	40	40	40	40	40	40	
Secondary metabolism													
Aromatics degradation													
M00538	Toluene degradation, toluene => benzoate	3	0	0	0	0	0	0	0	0	0	0	
M00537	Xylene degradation, xylene => methylbenzoate	3	0	0	0	0	0	0	0	0	0	0	
M00419	Cymene degradation, p-cymene => p-cumate	3	0	0	0	0	0	0	0	0	0	0	
M00547	Benzene/toluene degradation, benzene => catechol / toluene => 3-methylcatechol	2	0	0	0	0	0	0	0	0	0	0	
M00548	Benzene degradation, benzene => catechol	1	0	0	0	0	0	0	0	0	0	0	
M00551	Benzoate degradation, benzoate => catechol / methylbenzoate => methylcatechol	2	0	0	0	0	0	0	0	0	0	0	
M00568	Catechol ortho-cleavage, catechol => 3-oxoadipate	4	0	0	0	0	0	0	0	0	0	0	
M00569	Catechol meta-cleavage, catechol => acetyl-CoA / 4-methylcatechol => propanoyl-CoA	5..7	14.3	14.3	0	14.3	14.3	14.3	14.3	0	0	0	
M00539	Cumate degradation, p-cumate => 2-oxopent-4-enoate + 2-methylpropanoate	5	0	0	0	0	0	0	0	0	0	0	
M00543	Biphenyl degradation, biphenyl => 2-oxopent-4-enoate + benzoate	4	0	0	0	0	0	0	0	0	0	0	
M00544	Carbazole degradation, carbazole => 2-oxopent-4-enoate + anthranilate	3	0	0	0	0	0	0	0	0	0	0	
M00418	Toluene degradation, anaerobic, toluene => benzoyl-CoA	6	0	0	0	0	0	0	0	0	0	0	
M00541	Benzoyl-CoA degradation, benzoyl-CoA => 3-hydroxypimeloyl-CoA	4	0	0	0	0	0	0	0	0	0	0	
M00540	Benzoate degradation, cyclohexanecarboxylic acid =>pimeloyl-CoA	5	0	0	0	0	0	0	0	0	0	0	
M00534	Naphthalene degradation, naphthalene => salicylate	6	0	0	0	0	0	0	0	0	0	0	
M00623	Phthalate degradation, phthalate => protocatechuate	3	0	0	0	0	0	0	0	0	0	0	
M00624	Terephthalate degradation, terephthalate => 3,4-dihydroxybenzoate	2	0	0	0	0	0	0	0	0	0	0	

Table S3. CDSs catergorized into F1 in Figure 4. Functions related to polysaccharide metabolism are marked with yellow, while those related to transporters are marked with orange.

Contig_gene_name	BLASTP								KEGG (KAAS)	
	gi	Refseq ID	% identity	Length (aa)	e-value	Bit score	BLASTP description	Taxonomy	KO	KO description
AS_1_contig_23Ugene_74U96197U97144U+U11_1	503897724	WP_014131718.1	35.61	264	5.00E-29	123	cysteinyl-tRNA synthetase	Pelagibacterium halotolerans	K01884	cysS1; cysteinyl-tRNA synthetase, unknown class [EC:6.1.1.16]
AS_1_contig_23Ugene_78U100243U101085U+U11_1	497225970	WP_009540232.1	30.08	236	2.00E-18	92.4	hypothetical protein	Caenispirillum salinarum		
AS_1_contig_23Ugene_86U110116U110733U+U11_1	563572797	WP_023778017.1	29.66	118	9.00E-05	50.1	hypothetical protein	Mesorhizobium sp. LNHC221B00		
AS_1_contig_23Ugene_125U157358U157627U+U11_1	503715052	WP_013949128.1	36	75	3.00E-09	58.9	MULTISPECIES: hypothetical protein	Hyphomicrobium		
AS_1_contig_23Ugene_140U171849U174209U-U11_1	503712036	WP_013946112.1	69.97	786	0	1177	MULTISPECIES: acylaldehyde oxidase	Hyphomicrobium	K07303	Isoquinoline 1-oxidoreductase, beta subunit [EC:1.3.99.16]
AS_1_contig_23Ugene_141U174212U174664U-U11_1	503712035	WP_013946111.1	79.33	150	5.00E-84	255	MULTISPECIES: (2Fe-2S)-binding protein	Hyphomicrobium	K07302	Isoquinoline 1-oxidoreductase, alpha subunit [EC:1.3.99.16]
AS_1_contig_23Ugene_142U174768U176411U-U11_1	652473402	WP_026868097.1	78.4	551	0	826	lactate permease	Hyphomicrobium zavarzinii	K03303	TC.LCTP; lactate transporter, LctP family
AS_1_contig_23Ugene_144U177347U178363U+U11_1	563755589	WP_023786808.1	52.46	345	5.00E-104	321	hypothetical protein	Hyphomicrobium nitrativorans	K07402	xdhC; xanthine dehydrogenase accessory factor
AS_1_contig_23Ugene_146U180007U180585U-U11_1	635629399	WP_024276298.1	62.64	182	2.00E-65	211	hypothetical protein	Hyphomicrobium sp. 802		
AS_1_contig_23Ugene_159U193814U194086U+U11_1	518932127	WP_020088002.1	48.81	84	3.00E-21	90.5	hypothetical protein	Hyphomicrobium zavarzinii		
AS_1_contig_23Ugene_276U310931U312067U-U11_1	499828152	WP_011508886.1	35.21	284	2.00E-38	154	phage integrase	Nitrobacter hamburgensis		
AS_1_contig_23Ugene_326U367855U368793U+U11_1	518929487	WP_020085362.1	57.88	311	2.00E-130	385	hypothetical protein	Hyphomicrobium zavarzinii		
AS_1_contig_23Ugene_352U398072U399508U-U11_1	518761930	WP_019919219.1	37.72	342	5.00E-56	201	MULTISPECIES: hypothetical protein	Rhodocyclaceae	K03183	ubiE; ubiquinone/menaquinone biosynthesis methyltransferase [EC:2.1.1.163 2.1.1.201]
AS_1_contig_23Ugene_354U401279U401527U+U11_1	506409658	WP_015929377.1	39.47	76	2.00E-12	66.6	phosphopantetheine-binding protein	Methylobacterium nodulans		
AS_1_contig_23Ugene_355U401533U402657U-U11_1	657199172	WP_029314686.1	40.22	358	4.00E-81	263	hypothetical protein	Acidiphilium angustum		
AS_1_contig_23Ugene_382U430304U430486U+U11_1	639928191	WP_024764032.1	53.33	60	1.00E-11	63.5	cobalt transporter	Pseudomonas nitroreducens		
AS_1_contig_23Ugene_383U430496U431254U+U11_1	518928494	WP_020084369.1	49.6	248	7.00E-60	201	hypothetical protein	Hyphomicrobium zavarzinii		
AS_1_contig_23Ugene_394U442300U443415U-U11_1	652395684	WP_026791522.1	59.73	370	5.00E-141	417	cobalt-precorrin-6A synthase	Pleomorphomonas oryzae	K02188	cbiD; cobalt-precorrin-5B (C1)-methyltransferase [EC:2.1.1.195]
AS_1_contig_23Ugene_443U498380U499234U-U11_1	665825352	WP_031193469.1	79.21	279	7.00E-159	456	hypothetical protein, partial	Mesorhizobium sp. L2C066B000		
AS_1_contig_23Ugene_493U545374U546630U+U11_1	497449769	WP_009763967.1	44.92	423	3.00E-112	347	esterase	Microvirga lotononidis		
AS_1_contig_23Ugene_499U553419U553670U+U11_1	498340438	WP_010654594.1	61.45	83	2.00E-30	113	hypothetical protein	Fluoribacter dumoffii		
AS_1_contig_23Ugene_555U605289U605660U+U11_1	503712511	WP_013946587.1	70.49	122	9.00E-59	188	bleomycin resistance protein	Hyphomicrobium sp. MC1		
AS_1_contig_23Ugene_617U665948U666322U+U11_1	646804888	WP_025588472.1	32.97	91	7.00E-04	44.3	pilus assembly protein PilZ	Bradyrhizobium sp. JGI 0001002-A22		
AS_1_contig_23Ugene_654U703927U704193U+U11_1	652533640	WP_026927643.1	68.18	88	1.00E-36	129	hypothetical protein	Granulicoccus phenolivorans		
AS_1_contig_23Ugene_660U707981U708796U-U11_1	496438703	WP_009147548.1	25.74	272	1.00E-25	112	sulfotransferase family protein	Thiorhodovibrio sp. 970		
AS_1_contig_23Ugene_700U752564U753097U+U11_1	521960998	WP_020472603.1	50.3	167	2.00E-55	184	hypothetical protein	Zavarzinella formosa		
AS_1_contig_23Ugene_701U753274U753801U+U11_1	654897122	WP_028348667.1	37.29	177	2.00E-20	93.6	hypothetical protein	Bradyrhizobium elkanii		
AS_1_contig_23Ugene_718U770828U771919U+U11_1	490763656	WP_004625892.1	40.13	309	5.00E-82	268	Tfp pilus assembly protein, major pilin PilA	Clostridium] termitidis		
AS_1_contig_37Ugene_5U2608U2943U-U11_1	563762245	WP_023788192.1	29.11	79	3.00E-05	48.5	hypothetical protein	Hyphomicrobium nitrativorans		
AS_1_contig_37Ugene_15U11112U11525U-U11_1	501486636	WP_012495126.1	48.51	134	2.00E-33	125	hypothetical protein	Rhodopseudomonas palustris		
AS_1_contig_37Ugene_16U11755U12531U+U11_1	654360213	KEC69443.1	62.5	256	7.00E-107	322	transmembrane cytochrome b-type protein	Rhizobium leguminosarum bv. phaseoli CCGM1		
AS_1_contig_37Ugene_62U54954U55697U+U11_1	495826453	WP_008551032.1	37.39	238	5.00E-48	170	hypothetical protein	Pseudovibrio sp. JE062		
AS_1_contig_37Ugene_82U77736U78854U-U11_1	501060664	WP_012112003.1	41.98	343	6.00E-87	279	hypothetical protein	Parvibaculum lavamentivorans		
AS_1_contig_37Ugene_97U92188U92985U+U11_1	652459009	WP_026853818.1	31.6	250	1.00E-34	136	hypothetical protein	Geothrix fermentans		
AS_1_contig_37Ugene_98U93085U93450U-U11_1	518856904	WP_020012794.1	40.38	104	3.00E-06	51.6	MULTISPECIES: hypothetical protein	Agrobacterium		
AS_1_contig_37Ugene_139U136259U136690U+U11_1	503714187	WP_013948263.1	50	108	6.00E-28	115	hypothetical protein	Hyphomicrobium sp. MC1		
AS_1_contig_37Ugene_140U137137U137625U+U11_1	492478113	WP_005858867.1	43.75	112	1.00E-21	95.1	hypothetical protein	Sagittula stellata		
AS_1_contig_37Ugene_142U138049U138960U+U11_1	635740036	KDF21707.1	26.4	125	3.00E-06	56.2	hypothetical protein AF48_02671	Enterobacter aerogenes MGH 62		
AS_1_contig_37Ugene_173U170954U171652U+U11_1	563762890	WP_023788319.1	63.47	219	2.00E-89	275	hypothetical protein	Hyphomicrobium nitrativorans		
AS_1_contig_37Ugene_175U172591U173148U+U11_1	501062877	WP_012114166.1	36.84	95	1.00E-08	59.3	hypothetical protein	Xanthobacter autotrophicus		
AS_1_contig_37Ugene_177U174071U174430U+U11_1	518931876	WP_020087751.1	46.09	115	4.00E-26	104	hypothetical protein	Hyphomicrobium zavarzinii		
AS_1_contig_37Ugene_183U177314U178015U-U11_1	489706329	WP_003610466.1	79.83	233	5.00E-135	391	restriction endonuclease BglII	Methylosinus trichosporium		
AS_1_contig_37Ugene_202U199494U200270U+U11_1	494878903	WP_007604992.1	54.33	254	1.00E-89	277	hypothetical protein	Bradyrhizobium sp. WSM471		
AS_1_contig_37Ugene_211U213939U215186U+U11_1	653050240	WP_027301828.1	66.9	435	0	580	integrase	Rhodospirillales bacterium URHD0088		
AS_1_contig_37Ugene_213U216434U216616U-U11_1	501586676	WP_012590968.1	63.33	60	2.00E-16	75.9	hypothetical protein	Methylocella silvestris		
AS_1_contig_37Ugene_214U217181U218218U+U11_1	662507193	KEQ64802.1	53.67	300	2.00E-110	336	alpha/beta-hydrolase	Aureobasidium melanogenum CBS 110374		
AS_1_contig_37Ugene_217U220643U221380U+U11_1	494148551	WP_007088297.1	76.42	246	6.00E-138	399	membrane protein	Thalassospira xiamenensis	K03386	PRDX, ahpC; peroxiredoxin (alkyl hydroperoxide reductase subunit C) [EC:1.11.1.15]
AS_1_contig_37Ugene_218U221392U221889U+U11_1	497062043	WP_009452890.1	74.68	158	6.00E-82	251	hypothetical protein	Nitratireductor indicus		
AS_1_contig_37Ugene_221U225368U225649U+U11_1	501061238	WP_012112561.1	45.1	51	2.00E-05	48.5	hypothetical protein	Xanthobacter autotrophicus		
AS_1_contig_37Ugene_222U225639U229316U+U11_1	499828446	WP_011509180.1	50.81	185	1.00E-41	176	hypothetical protein	Nitrobacter hamburgensis		
AS_1_contig_37Ugene_222U225639U229316U+U11_1	499828446	WP_011509180.1	53.25	169	8.00E-40	171	hypothetical protein	Nitrobacter hamburgensis		
AS_1_contig_37Ugene_222U225639U229316U+U11_1	499828446	WP_011509180.1	48.65	111	2.00E-22	114	hypothetical protein	Nitrobacter hamburgensis		
AS_1_contig_37Ugene_222U225639U229316U+U11_1	499828446	WP_011509180.1	45.45	132	5.00E-22	113	hypothetical protein	Nitrobacter hamburgensis		
AS_1_contig_37Ugene_222U225639U229316U+U11_1	499828446	WP_011509180.1	38.92	185	8.00E-22	112	hypothetical protein	Nitrobacter hamburgensis		
AS_1_contig_37Ugene_222U225639U229316U+U11_1	499828446	WP_011509180.1	50.44	113	1.00E-21	112	hypothetical protein	Nitrobacter hamburgensis		
AS_1_contig_37Ugene_222U225639U229316U+U11_1	499828446	WP_011509180.1	43.14	153	3.00E-19	104	hypothetical protein	Nitrobacter hamburgensis		
AS_1_contig_37Ugene_225U230707U231114U+U11_1	522151032	WP_020662240.1	41.57	89	5.00E-07	57.4	glycoside hydrolase	Amycolatopsis benzoatilytica		
AS_1_contig_37Ugene_227U231985U232209U+U11_1	327192988	EGE59902.1	75	76	3.00E-30	112	hypothetical protein RHECNPAF_178003	Rhizobium etli CNPAF512		
AS_1_contig_37Ugene_228U232240U232530U+U11_1	640172565	WP_024810226.1	50	96	6.00E-20	87.4	hypothetical protein	Oceanicola sp. HL-35		
AS_1_contig_37Ugene_232U234958U235161U-U11_1	501423687	WP_012448357.1	45.65	46	0.002	44.7	ankyrin	Natranaerobius thermophilus		
AS_1_contig_37Ugene_233U235257U235556U-U11_1	501586688	WP_012590980.1	54.26	94	1.00E-28	110	hypothetical protein	Methylocella silvestris		
AS_1_contig_37Ugene_234U235920U236252U+U11_1	497513207	WP_009827405.1	32.97	91	4.00E-09	60.1	hypothetical protein	Sulfitobacter sp. NAS-14.1		
AS_1_contig_37Ugene_235U236242U237621U+U11_1	652861981	WP_027132570.1	34.16	404	6.00E-61	216	hypothetical protein	Geminicoccus roseus		
AS_1_contig_37Ugene_236U237704U238087U+U11_1	327192993	EGE59907.1	55.12	127	9.00E-40	140	hypothetical protein RHECNPAF_178008	Rhizobium etli CNPAF512		
AS_1_contig_37Ugene_237U238089U238568U-U11_1	511759724	WP_016392938.1	46.1	154	8.00E-40	142	bifunctional autolysin AtlE/transcriptional regulator AtlR	Pseudomonas plecoglossicida		
AS_1_contig_37Ugene_238U238680U239987U-U11_1	652382625	WP_026778553.1	54.7	415	2.00E-150	447	hypothetical protein	Polaromonas sp. EUR3 1.2.1		
AS_1_contig_37Ugene_239U239984U240448U-U11_1	504869683	WP_015056785.1	64.14	145	4.00E-58	190	hypothetical protein	Fibrella aestuarina		
AS_1_contig_37Ugene_240U240478U240867U-U11_1	652473160	WP_026867855.1	58.33	108	3.00E-36	131	hypothetical protein	Hyphomicrobium zavarzinii		
AS_1_contig_37Ugene_242U244012U245520U-U11_1	652473159	WP_026867854.1	59.06	342	2.00E-129	400	hypothetical protein	Hyphomicrobium zavarzinii		
AS_1_contig_37Ugene_242U244012U245520U-U11_1	652473159	WP_026867854.1	52.94	153	6.00E-39	159	hypothetical protein	Hyphomicrobium zavarzinii		
AS_1_contig_37Ugene_247U251744U252076U-U11_1	518928980	WP_020084855.1	38.74	111	6.00E-16	77.8	hypothetical protein	Hyphomicrobium zavarzinii		
AS_1_contig_37Ugene_262U266256U266714U+U11_1	518930491	WP_020086366.1	66.18	136	3.00E-55	181	hypothetical protein	Hyphomicrobium zavarzinii		
AS_1_contig_37Ugene_264U267136U267462U+U11_1	563760606	WP_023787865.1	45.83	96	9.00E-21	90.1	hypothetical protein	Hyphomicrobium nitrativorans		
AS_1_contig_37Ugene_280U284865U286946U-U11_1	517219484	WP_018408302.1	59.8	704	0	758	hypothetical protein	Methylocystis rosea	K07114	yfbK; Ca-activated chloride channel homolog
AS_1_contig_37Ugene_343U345775U347082U-U11_1	658445379	WP_029671147.1	67.71	415	0	568	C4-dicarboxylate transporter	Hyphomicrobium sp. 802	K11103	dctA; aerobic C4-dicarboxylate transport protein
AS_1_contig_37Ugene_348U351727U352272U-U11_1	563551787	WP_023757560.1	53.04	181	2.00E-60	197	heme transporter CcmB	Mesorhizobium sp. LNHC252B00		
AS_1_contig_37Ugene_375U389449U389943U-U11_1	635628167	WP_024275066.1	44.74	152	6.00E-36	133	hypothetical protein	Hyphomicrobium sp. 802		
AS_1_contig_37Ugene_394U409360U409908U+U11_1	660652016	KEO91734.1	51.69	178	2.00E-55	185	hypothetical protein EH31_03420	Erythrobacter longus		
AS_1_contig_37Ugene_416U430424U431218U-U11_1	492482893	WP_005860197.1	51.17	213	4.00E-64	212	hypothetical cytosolic protein	Sagittula stellata		
AS_1_contig_37Ugene_419U433578U434231U+U11_1	503713211	WP_013947287.1	39.31	173	5.00E-32	126	MULTISPECIES: hypothetical protein	Hyphomicrobium		
AS_1_contig_37Ugene_420U434318U435052U-U11_1	630772816	KCZ54505.1	38.74	191	2.00E-34	134	hypothetical protein HY30_09470	Hyphomonas sp. BH-BN04-4		
AS_1_contig_37Ugene_451U473625U474230U+U11_1	563566653	WP_023772150.1	63.69	179	7.00E-74	233	MULTISPECIES: membrane protein	Mesorhizobium		

AS_1_contig_37Ugene_502U531320U531772U+U11_1	518929812	WP_020085687.1	48.12	133	6.00E-41	144	hypothetical protein	Hyphomicrobium zavarzinii		
AS_1_contig_37Ugene_504U532891U533871U+U11_1	491151835	WP_005010229.1	29.56	159	2.00E-10	71.2	hypothetical protein	Nitrospina gracilis		
AS_1_contig_37Ugene_507U536544U537242U+U11_1	494678607	WP_007435942.1	38.83	206	3.00E-32	132	hypothetical protein	Acetobacteraceae bacterium AT-5844		
AS_1_contig_37Ugene_539U564412U565299U-U11_1	655999209	WP_029040683.1	45.02	291	7.00E-64	214	hypothetical protein	Cucumibacter marinus		
AS_1_contig_37Ugene_552U574138U574563U-U11_1	517727390	WP_018897598.1	65.45	110	2.00E-38	145	hypothetical protein	Rhizobium sp. 2MFCol3.1	K03316	TC.CPA1; monovalent cation:H+ antiporter, CPA1 family
AS_1_contig_37Ugene_553U574787U575572U-U11_1	655629023	KED29197.1	37.6	250	6.00E-41	158	hypothetical protein EM98_07540	Vibrio parahaemolyticus		
AS_1_contig_110Ugene_2U1069U2181U+U11_1	496151739	WP_008876246.1	61.62	370	1.00E-152	447	conserved hypothetical protein	Mesorhizobium metallidurans		
AS_1_contig_110Ugene_5U4740U5000U-U11_1	563534853	WP_023741013.1	74.7	83	2.00E-35	126	acyl carrier protein	Mesorhizobium sp. LNJC395A00		
AS_1_contig_110Ugene_28U33362U34600U+U11_1	505063066	WP_015250168.1	34.06	367	1.00E-59	211	hypothetical protein	Singulisphaera acidiphila		
AS_1_contig_110Ugene_36U43387U43788U+U11_1	503716386	WP_013950462.1	46.03	126	2.00E-24	101	hypothetical protein	Hyphomicrobium sp. MC1		
AS_1_contig_118Ugene_3U1671U2492U-U11_1	496258491	WP_008971876.1	38.55	275	2.00E-62	209	conserved hypothetical protein	Bradyrhizobium sp. STM 3843		
AS_1_contig_118Ugene_4U2525U2839U-U11_1	635628903	WP_024275802.1	69.9	103	3.00E-42	145	RND transporter	Hyphomicrobium sp. 802		
AS_1_contig_118Ugene_5U2895U3386U-U11_1	658445185	WP_029670953.1	66.67	120	6.00E-54	179	copper resistance protein	Hyphomicrobium sp. 802		
AS_1_contig_118Ugene_7U4813U6132U-U11_1	658445184	WP_029670952.1	81.22	426	0	679	copper resistance protein, partial	Hyphomicrobium sp. 802		
AS_1_contig_118Ugene_8U6281U6526U-U11_1	82940267	CAJ19376.1	45.21	73	2.00E-08	56.6	hypothetical protein	Pedomicrobium sp. ACM 3067		
AS_1_contig_118Ugene_20U18778U19683U-U11_1	522139633	WP_020650842.1	38.85	278	2.00E-39	152	hypothetical protein	Solimonas variicoloris	K01062	PAFAH; platelet-activating factor acetylhydrolase [EC:3.1.1.47]
AS_1_contig_118Ugene_26U35653U37113U+U11_1	505056084	WP_015243186.1	48.16	463	1.00E-112	353	hypothetical protein	Sinorhizobium meliloti		
AS_1_contig_118Ugene_30U40162U41718U+U11_1	515837677	WP_017268430.1	50.86	466	4.00E-157	468	hypothetical protein	Sinorhizobium meliloti		
AS_1_contig_118Ugene_31U41733U42503U-U11_1	503715574	WP_013949650.1	49.79	243	8.00E-81	254	MULTISPECIES: hypothetical protein	Hyphomicrobium		
AS_1_contig_118Ugene_34U45294U46574U+U11_1	564006195	WP_023832412.1	64.71	357	1.00E-170	497	glycosyl transferase	Mesorhizobium sp. L103C119B0		
AS_1_contig_118Ugene_39U51221U52237U+U11_1	658529949	WP_029724262.1	66.45	313	2.00E-143	421	hypothetical protein	Sphingomonas sp. JGI 0001003-D23	K02297	cyoA; cytochrome o ubiquinol oxidase subunit II [EC:1.10.3.-]
AS_1_contig_118Ugene_42U54872U55267U+U11_1	659889653	WP_029936337.1	61.67	120	8.00E-38	135	hypothetical protein	Sphingomonas sp. UNC305MFCol5.2	K02300	cyoD; cytochrome o ubiquinol oxidase operon protein cyoD
AS_1_contig_118Ugene_126U143192U143725U-U11_1	635629287	WP_024276186.1	70.11	174	1.00E-83	258	NAD-dependent deacetylase	Hyphomicrobium sp. 802	K12410	npdA; NAD-dependent deacetylase [EC:3.5.1.-]
AS_1_contig_118Ugene_154U171110U171604U+U11_1	518931414	WP_020087289.1	47.73	132	4.00E-27	109	hypothetical protein	Hyphomicrobium zavarzinii		
AS_1_contig_118Ugene_203U222763U223380U-U11_1	657604063	WP_029415312.1	45.86	181	7.00E-40	146	hypothetical protein	Brevundimonas bacteroides		
AS_1_contig_118Ugene_204U223597U223986U+U11_1	503713493	WP_013947569.1	65.08	126	2.00E-49	167	hypothetical protein	Hyphomicrobium sp. MC1		
AS_1_contig_118Ugene_224U244857U245345U-U11_1	563761341	WP_023788017.1	41.26	143	1.00E-19	90.9	hypothetical protein	Hyphomicrobium nitrativorans		
AS_1_contig_118Ugene_226U246781U247617U+U11_1	563763302	WP_023788388.1	52.42	124	1.00E-33	130	hypothetical protein	Hyphomicrobium nitrativorans		
AS_1_contig_118Ugene_227U247751U248881U+U11_1	517827250	WP_018997458.1	47.37	285	5.00E-85	273	hypothetical protein	Hirschia maritima	K05195	GLRA3; glycine receptor alpha-3
AS_1_contig_118Ugene_238U262915U264945U-U11_1	503184856	WP_013419517.1	36.83	649	8.00E-119	380	hypothetical protein	Rhodomicrobium vannielii		
AS_1_contig_118Ugene_239U264998U265915U-U11_1	503184857	WP_013419518.1	47.65	277	2.00E-78	253	hypothetical protein	Rhodomicrobium vannielii		
AS_1_contig_118Ugene_246U272396U276946U-U11_1	652915313	WP_027169244.1	43.71	501	9.00E-109	371	hypothetical protein	Mesorhizobium sp. WSM3224		
AS_1_contig_118Ugene_255U290362U290799U-U11_1	503712431	WP_013946507.1	61.27	142	1.00E-49	167	hypothetical protein	Hyphomicrobium sp. MC1		
AS_1_contig_118Ugene_298U334261U335577U+U11_1	648499242	WP_026190993.1	30.03	323	8.00E-25	116	hypothetical protein	Methylobacterium sp. WSM2598		
AS_1_contig_118Ugene_299U335650U338391U-U11_1	653406387	WP_027520844.1	38.59	907	5.00E-177	547	hypothetical protein	Bradyrhizobium sp. Ec3.3	K10407	KLC; kinesin light chain
AS_1_contig_118Ugene_305U342862U343296U+U11_1	639251607	WP_024576410.1	75.86	145	6.00E-50	167	MULTISPECIES: hypothetical protein	Afipia		
AS_1_contig_118Ugene_306U343522U344745U-U11_1	658434473	WP_029660810.1	38.62	391	1.00E-77	257	hypothetical protein, partial	Afipia sp. OHSU_II-C2		
AS_1_contig_118Ugene_307U344854U345141U+U11_1	494445385	WP_007238159.1	35.29	85	1.00E-04	48.9	RNA-directed DNA polymerase	Gordonia otitidis		
AS_1_contig_118Ugene_308U345143U346585U+U11_1	518929489	WP_020085364.1	47.32	467	1.00E-115	361	hypothetical protein	Hyphomicrobium zavarzinii		
AS_1_contig_118Ugene_309U346582U347802U+U11_1	639249989	WP_024575873.1	52.9	397	2.00E-144	429	MULTISPECIES: glycosyl transferase	Afipia	K02840	waaB, rfaB; UDP-D-galactose:(glucosyl)LPS alpha-1,6-D-galactosyltransferase [EC:2.4.1.-]
AS_1_contig_118Ugene_310U347827U348963U-U11_1	653552281	WP_027582021.1	40.16	371	1.00E-80	263	glycoside hydrolase	Bradyrhizobium sp. Ai1a-2		
AS_1_contig_118Ugene_322U360971U361876U-U11_1	563555119	WP_023760877.1	72.19	302	3.00E-152	440	restriction endonuclease	Mesorhizobium sp. LNHC252B00	K07448	mrr; restriction system protein
AS_1_contig_118Ugene_326U365955U366392U-U11_1	503711658	WP_013945734.1	45.27	148	1.00E-26	108	hypothetical protein	Hyphomicrobium sp. MC1		
AS_1_contig_118Ugene_328U366785U367987U+U11_1	635629585	WP_024276484.1	52.36	403	6.00E-147	436	hypothetical protein	Hyphomicrobium sp. 802	K05841	E2.4.1.173; sterol 3beta-glucosyltransferase [EC:2.4.1.173]
AS_1_contig_118Ugene_353U394782U395123U-U11_1	503712750	WP_013946826.1	60.94	64	3.00E-08	57	hypothetical protein	Hyphomicrobium sp. MC1		
AS_1_contig_118Ugene_370U414846U416417U+U11_1	504047260	WP_014281254.1	26.77	467	4.00E-38	155	hypothetical protein	Paenibacillus terrae		
AS_1_contig_118Ugene_377U421958U423487U-U11_1	652920566	WP_027174442.1	38.97	503	1.00E-95	311	hypothetical protein	Methylobacterium sp. 10		
AS_1_contig_118Ugene_381U426547U426801U-U11_1	550970023	WP_022718312.1	54.17	72	7.00E-18	81.6	membrane protein	Rhizobium mongolense		
AS_1_contig_118Ugene_452U486597U488111U+U11_1	496257685	WP_008971070.1	61.94	494	0	655	magnesium-protoporphyrin IX monomethyl ester cyclase	Bradyrhizobium sp. STM 3843	K04034	bchE; anaerobic magnesium-protoporphyrin IX monomethyl ester cyclase [EC:4.-.-.-]
AS_1_contig_118Ugene_477U516405U516743U+U11_1	503712392	WP_013946468.1	37.31	67	4.00E-06	51.2	hypothetical protein	Hyphomicrobium sp. MC1		
AS_1_contig_118Ugene_478U516792U517475U+U11_1	544689024	WP_021120629.1	48.12	133	3.00E-29	120	hypothetical protein	Salipiger mucosus		
AS_1_contig_118Ugene_480U518854U519198U+U11_1	640212996	WP_024824072.1	44.95	109	7.00E-15	79.3	hypothetical protein	Desulfovibrio magneticus		
AS_1_contig_118Ugene_482U519884U520408U+U11_1	640216962	WP_024825590.1	48.87	133	2.00E-33	126	hypothetical protein	Desulfovibrio magneticus		
AS_1_contig_118Ugene_483U520401U522017U+U11_1	589890396	YP_009005927.1	55.54	542	0	578	terminase large subunit	Rhizobium phage vB_RglS_P106B	K06909	xtmB; phage terminase large subunit
AS_1_contig_118Ugene_488U525372U525611U+U11_1	503713559	WP_013947635.1	50	54	3.00E-12	66.2	hypothetical protein	Hyphomicrobium sp. MC1		
AS_1_contig_118Ugene_489U525637U525954U+U11_1	503713558	WP_013947634.1	48.84	43	0.002	44.7	hypothetical protein	Hyphomicrobium sp. MC1		
AS_1_contig_118Ugene_490U526089U526319U-U11_1	518929832	WP_020085707.1	42.25	71	4.00E-06	50.1	hypothetical protein	Hyphomicrobium zavarzinii		
AS_1_contig_118Ugene_491U526651U527208U-U11_1	503714381	WP_013948457.1	54.22	166	1.00E-45	159	hypothetical protein	Hyphomicrobium sp. MC1		
AS_1_contig_118Ugene_518U557555U558652U-U11_1	517827474	WP_018997682.1	45.13	113	7.00E-21	101	hypothetical protein	Hirschia maritima		
AS_1_contig_160Ugene_1U15U995U-U11_1	505279445	WP_015466547.1	43.64	220	1.00E-42	167	hypothetical protein	Micavibrio aeruginosavorus		
AS_1_contig_160Ugene_1U15U995U-U11_1	505279445	WP_015466547.1	38.36	232	7.00E-33	139	hypothetical protein	Micavibrio aeruginosavorus		
AS_1_contig_160Ugene_1U15U995U-U11_1	505279445	WP_015466547.1	35.9	234	9.00E-27	121	hypothetical protein	Micavibrio aeruginosavorus		
AS_1_contig_160Ugene_1U15U995U-U11_1	505279445	WP_015466547.1	32.13	221	2.00E-25	117	hypothetical protein	Micavibrio aeruginosavorus		
AS_1_contig_160Ugene_1U15U995U-U11_1	505279445	WP_015466547.1	40.38	156	9.00E-22	106	hypothetical protein	Micavibrio aeruginosavorus		
AS_1_contig_160Ugene_1U15U995U-U11_1	505279445	WP_015466547.1	33.63	226	1.00E-20	102	hypothetical protein	Micavibrio aeruginosavorus		
AS_1_contig_160Ugene_1U15U995U-U11_1	505279445	WP_015466547.1	41.74	115	4.00E-17	92	hypothetical protein	Micavibrio aeruginosavorus		
AS_1_contig_160Ugene_1U15U995U-U11_1	505279445	WP_015466547.1	29.84	191	6.00E-15	85.1	hypothetical protein	Micavibrio aeruginosavorus		
AS_1_contig_160Ugene_1U15U995U-U11_1	505279445	WP_015466547.1	49.41	85	2.00E-09	68.2	hypothetical protein	Micavibrio aeruginosavorus		
AS_1_contig_160Ugene_2U1000U2979U-U11_1	655000506	WP_028449699.1	38.85	157	1.00E-24	116	hypothetical protein	Chitinibacter tainanensis		
AS_1_contig_160Ugene_3U2972U3250U-U11_1	501061238	WP_012112561.1	34.48	58	0.006	41.6	hypothetical protein	Xanthobacter autotrophicus		
AS_1_contig_160Ugene_5U4625U5683U-U11_1	518928942	WP_020084817.1	46.92	341	4.00E-96	301	hypothetical protein	Hyphomicrobium zavarzinii		
AS_1_contig_160Ugene_6U6329U6598U+U11_1	630772009	KCZ53736.1	61.9	63	6.00E-22	91.7	hypothetical protein HY29_16130	Hyphomonas sp. 25B14_1		
AS_1_contig_160Ugene_8U8583U9164U-U11_1	497499337	WP_009813535.1	40	90	2.00E-14	77.4	hypothetical protein	Roseovarius nubinhibens		
AS_1_contig_160Ugene_9U9226U10467U-U11_1	518928938	WP_020084813.1	28.69	373	3.00E-34	142	hypothetical protein	Hyphomicrobium zavarzinii		
AS_1_contig_160Ugene_11U10968U12095U+U11_1	518928929	WP_020084804.1	38.97	136	5.00E-18	94	hypothetical protein	Hyphomicrobium zavarzinii		
AS_1_contig_160Ugene_12U12092U12460U+U11_1	518928930	WP_020084805.1	48.1	79	2.00E-14	73.6	hypothetical protein	Hyphomicrobium zavarzinii		
AS_1_contig_160Ugene_13U12499U12846U+U11_1	518928930	WP_020084805.1	53.49	86	4.00E-22	93.6	hypothetical protein	Hyphomicrobium zavarzinii	K17512	SHARK; tyrosine-protein kinase shark [EC:2.7.10.2]
AS_1_contig_160Ugene_14U13392U13748U+U11_1	630772010	KCZ53737.1	48	100	1.00E-22	95.9	hypothetical protein HY29_16135	Hyphomonas sp. 25B14_1		
AS_1_contig_160Ugene_15U13738U15147U+U11_1	518928931	WP_020084806.1	37.84	444	1.00E-89	292	hypothetical protein	Hyphomicrobium zavarzinii		
AS_1_contig_160Ugene_18U16831U19407U+U11_1	651323408	WP_026447764.1	47.32	858	0	746	hypothetical protein	Acidobacteriaceae bacterium URHE0068		
AS_1_contig_160Ugene_39U39993U40790U-U11_1	496257610	WP_008970995.1	38.76	258	2.00E-56	194	putative ATP-transporter permease	Bradyrhizobium sp. STM 3843	K01992	ABC-2.P; ABC-2 type transport system permease protein
AS_1_contig_160Ugene_43U43894U44829U+U11_1	503713365	WP_013947441.1	49.49	293	3.00E-92	288	dTDP-4-dehydrorhamnose reductase	Hyphomicrobium sp. MC1	K00067	rfbD; dTDP-4-dehydrorhamnose reductase [EC:1.1.1.133]
AS_1_contig_160Ugene_45U45789U46889U-U11_1	654262799	WP_027778025.1	35.83	360	2.00E-56	199	glycosyl transferase family 1	Burkholderia caledonica		
AS_1_contig_160Ugene_89U92548U93585U+U11_1	635628448	WP_024275347.1	64.51	324	3.00E-150	439	XdhC/CoxI family protein	Hyphomicrobium sp. 802	K07402	xdhC; xanthine dehydrogenase accessory factor
AS_1_contig_160Ugene_90U93582U94181U+U11_1	635628447	WP_024275346.1	48.22	197	5.00E-58	192	hypothetical protein	Hyphomicrobium sp. 802	K07141	mocA; molybdenum cofactor cytidylyltransferase [EC:2.7.7.76]

AS_1_contig_160Ugene_140U143141U143290U+U11_1	503714758	WP_013948834.1	51.06	47	5.00E-05	45.4	hypothetical protein	Hyphomicrobium sp. MC1		
AS_1_contig_160Ugene_142U144225U144422U+U11_1	503711660	WP_013945736.1	71.67	60	5.00E-23	93.2	membrane protein	Hyphomicrobium sp. MC1		
AS_1_contig_160Ugene_150U150203U150430U+U11_1	504886346	WP_015073448.1	47.27	55	0.008	40	hypothetical protein	Gluconobacter oxydans		
AS_1_contig_160Ugene_156U156974U157354U-U11_1	518931544	WP_020087419.1	62.22	90	5.00E-24	103	hypothetical protein	Hyphomicrobium zavarzinii		
AS_1_contig_160Ugene_157U157621U157812U-U11_1	640420119	WP_024899196.1	45.9	61	1.00E-05	48.9	hypothetical protein	Ochrobactrum rhizosphaerae		
AS_1_contig_160Ugene_177U175398U176327U-U11_1	653048109	WP_027299706.1	45.86	290	7.00E-74	246	UbiA prenyltransferase	Rhodospirillales bacterium URHD0088		
AS_1_contig_160Ugene_178U176341U177819U-U11_1	499212873	WP_010910413.1	37.47	483	9.00E-86	285	hypothetical protein	Mesorhizobium loti		
AS_1_contig_160Ugene_215U225138U225590U+U11_1	310722519	YP_003969342.1	50.34	145	5.00E-42	148	unnamed protein product	Aeromonas phage phiAS5		
AS_1_contig_160Ugene_229U236712U237614U+U11_1	504902693	WP_015089795.1	47.5	280	6.00E-72	234	EamA-like transporter	Bdellovibrio bacteriovorus		
AS_1_contig_160Ugene_230U237724U238107U+U11_1	550971578	WP_022719792.1	51.91	131	2.00E-27	108	hypothetical protein	Rhodopseudomonas sp. B29		
AS_1_contig_160Ugene_249U259181U259519U-U11_1	652513390	WP_026907614.1	38.38	99	1.00E-10	64.7	serine protease	Paucisalibacillus globulus		
AS_1_contig_160Ugene_265U275723U276682U-U11_1	495821779	WP_008546358.1	34.84	287	2.00E-47	172	N-acetylglucosaminyltransferase	Pseudovibrio sp. JE062	K00737	MGAT3; beta-1,4-mannosyl-glycoprotein beta-1,4-N-acetylglucosaminyltransferase [EC:2.4.1.144]
AS_1_contig_160Ugene_273U281758U281952U+U11_1	635628827	WP_024275726.1	69.64	56	5.00E-18	80.1	hypothetical protein	Hyphomicrobium sp. 802		
AS_1_contig_160Ugene_279U288289U288867U-U11_1	515892921	WP_017323504.1	59.12	159	2.00E-63	206	hypothetical protein	cyanobacterium PCC 7702		
AS_1_contig_160Ugene_280U289124U289429U+U11_1	653425796	WP_027529212.1	29.21	89	9.00E-05	46.6	hypothetical protein	Bradyrhizobium sp. WSM3983		
AS_1_contig_186Ugene_2U507U923U-U11_1	503712931	WP_013947007.1	57.81	128	1.00E-34	127	hypothetical protein	Hyphomicrobium sp. MC1		
AS_1_contig_186Ugene_4U1499U2002U+U11_1	496252998	WP_008966383.1	57.14	161	2.00E-52	176	MarR family transcriptional regulator	Bradyrhizobium sp. STM 3809	K15973	mhqR; MarR family transcriptional regulator, 2-MHQ and catechol-resistance regulon repressor
AS_1_contig_186Ugene_7U3759U4100U-U11_1	495154324	WP_007879128.1	58.93	112	3.00E-40	142	BadM/Rrf2 family transcriptional regulator	Ochrobactrum sp. CDB2		
AS_1_contig_186Ugene_18U14720U14998U+U11_1	518931375	WP_020087250.1	49.32	73	1.00E-08	57.4	hypothetical protein	Hyphomicrobium zavarzinii		
AS_1_contig_186Ugene_21U18189U18566U-U11_1	515486429	WP_016919697.1	62.4	125	1.00E-46	157	hypothetical protein	Methylocystis parvus		
AS_1_contig_186Ugene_22U18725U18943U+U11_1	635629413	WP_024276312.1	62.12	66	3.00E-22	91.7	hypothetical protein	Hyphomicrobium sp. 802		
AS_1_contig_186Ugene_24U20702U21166U-U11_1	652473612	WP_026868307.1	70	150	3.00E-70	220	membrane protein	Hyphomicrobium zavarzinii		
AS_1_contig_186Ugene_25U21163U21669U-U11_1	497486016	WP_009800214.1	59.76	164	7.00E-65	207	hypothetical protein	Nitrobacter sp. Nb-311A		
AS_1_contig_186Ugene_28U26083U26394U+U11_1	563752339	WP_023786121.1	59.22	103	1.00E-26	105	transcriptional regulator	Hyphomicrobium nitrativorans		
AS_1_contig_186Ugene_31U29444U29755U+U11_1	563752339	WP_023786121.1	62.75	102	2.00E-29	112	transcriptional regulator	Hyphomicrobium nitrativorans		
AS_1_contig_186Ugene_37U33960U34172U+U11_1	665826648	WP_031194765.1	47.54	61	9.00E-14	69.7	hypothetical protein	Mesorhizobium sp. LSHC420B00		
AS_1_contig_186Ugene_40U37129U37995U+U11_1	617475838	KAI93867.1	69.41	255	4.00E-123	373	conjugal transfer protein TraA	Rhodomicrobium udaipurense JA643		
AS_1_contig_186Ugene_41U38037U38597U+U11_1	617475845	KAI93874.1	40.11	187	8.00E-24	102	hypothetical protein T281_14160	Rhodomicrobium udaipurense JA643		
AS_1_contig_186Ugene_42U38866U40050U+U11_1	502884982	WP_013119958.1	41.09	387	2.00E-97	308	hypothetical protein	Thermincola potens		
AS_1_contig_186Ugene_43U40052U40525U+U11_1	519025957	WP_020181832.1	40.26	154	4.00E-34	128	hypothetical protein	Methylotenera sp. 1P/1		
AS_1_contig_186Ugene_44U40797U46100U+U11_1	503156186	WP_013390847.1	37.98	1785	0	1129	hypothetical protein	Achromobacter xylosoxidans		
AS_1_contig_186Ugene_46U47011U47469U-U11_1	643805228	WP_025258671.1	52.35	149	1.00E-48	165	hypothetical protein	Pseudomonas cichorii		
AS_1_contig_186Ugene_47U47650U49803U-U11_1	655485187	WP_028866822.1	40.34	533	2.00E-97	337	hypothetical protein	Ralstonia sp. JGI 0001001-B07		
AS_1_contig_186Ugene_47U47650U49803U-U11_1	655485187	WP_028866822.1	35.38	537	1.00E-56	219	hypothetical protein	Ralstonia sp. JGI 0001001-B07		
AS_1_contig_186Ugene_47U47650U49803U-U11_1	655485187	WP_028866822.1	45.28	318	4.00E-55	215	hypothetical protein	Ralstonia sp. JGI 0001001-B07		
AS_1_contig_186Ugene_47U47650U49803U-U11_1	655485187	WP_028866822.1	32.21	149	2.00E-07	64.7	hypothetical protein	Ralstonia sp. JGI 0001001-B07		
AS_1_contig_186Ugene_49U50744U50977U-U11_1	517202515	WP_018391333.1	38.89	72	5.00E-04	45.8	hypothetical protein	Xanthobacteraceae bacterium 501b		
AS_1_contig_186Ugene_51U53365U53580U+U11_1	518928528	WP_020084403.1	49.09	55	6.00E-09	57	hypothetical protein	Hyphomicrobium zavarzinii		
AS_1_contig_186Ugene_53U54926U55234U-U11_1	587641753	EWY40766.1	50	86	4.00E-17	80.1	hypothetical protein N825_33090	Skermanella stibiiresistens SB22		
AS_1_contig_186Ugene_57U56861U57166U+U11_1	516752586	WP_018083781.1	38.46	104	5.00E-17	80.5	hypothetical protein	Asticcacaulis benevestitus		
AS_1_contig_186Ugene_62U61956U62609U+U11_1	499469553	WP_011156193.1	28.89	225	7.00E-08	58.5	hypothetical protein	Rhodopseudomonas palustris		
AS_1_contig_186Ugene_65U63353U64039U+U11_1	648488351	WP_026180102.1	38.16	76	5.00E-07	55.8	hypothetical protein	Hahella ganghwensis		
AS_1_contig_186Ugene_66U64116U64400U+U11_1	504860984	WP_015048086.1	38.57	70	9.00E-09	57.8	hypothetical protein	Simiduia agarivorans		
AS_1_contig_186Ugene_68U66452U66871U+U11_1	662774103	WP_030137261.1	35.23	88	3.00E-04	48.1	hypothetical protein	Mycobacterium neoaurum		
AS_1_contig_186Ugene_69U67002U67844U+U11_1	488714050	WP_002637926.1	32.91	158	3.00E-08	61.6	hypothetical protein	Myxococcus sp. (contaminant ex DSM 436)		
AS_1_contig_186Ugene_79U73653U73982U-U11_1	563752354	WP_023786124.1	40.28	72	5.00E-08	56.2	hypothetical protein	Hyphomicrobium nitrativorans		
AS_1_contig_186Ugene_82U76966U77265U+U11_1	503184443	WP_013419104.1	62.96	81	7.00E-31	115	hypothetical protein	Rhodomicrobium vannielii		
AS_1_contig_186Ugene_87U78172U78498U+U11_1	518432622	WP_019602829.1	42.42	99	7.00E-15	75.1	hypothetical protein	Teredinibacter turnerae		
AS_1_contig_186Ugene_105U94496U95815U-U11_1	563752533	WP_023786156.1	42.46	325	1.00E-70	237	hypothetical protein	Hyphomicrobium nitrativorans		
AS_1_contig_186Ugene_106U95730U96191U-U11_1	563752522	WP_023786154.1	56.15	130	3.00E-43	150	hypothetical protein	Hyphomicrobium nitrativorans		
AS_1_contig_186Ugene_108U97988U98578U+U11_1	563752511	WP_023786152.1	31.71	164	5.00E-06	53.5	hypothetical protein	Hyphomicrobium nitrativorans		
AS_1_contig_186Ugene_110U99196U99405U-U11_1	563752505	WP_023786151.1	42.11	57	1.00E-04	45.1	hypothetical protein	Hyphomicrobium nitrativorans		
AS_1_contig_186Ugene_111U99402U99563U-U11_1	563752487	WP_023786148.1	61.82	55	1.00E-14	70.9	hypothetical protein	Hyphomicrobium nitrativorans		
AS_1_contig_186Ugene_112U99560U99808U-U11_1	517202529	WP_018391347.1	44.44	54	2.00E-09	58.5	MULTISPECIES: hypothetical protein	Xanthobacteraceae		
AS_1_contig_186Ugene_114U101590U102435U+U11_1	488801098	WP_002713504.1	35.31	286	3.00E-43	159	hypothetical protein	Afipia clevelandensis		
AS_1_contig_186Ugene_117U106336U107124U-U11_1	562967238	ESW93124.1	48.39	248	3.00E-84	264	hypothetical protein X770_00815	Mesorhizobium sp. LSJC269B00		
AS_1_contig_186Ugene_118U107224U107772U-U11_1	516032823	WP_017463406.1	64.04	178	7.00E-84	257	hypothetical protein	Dyella ginsengisoli		
AS_1_contig_186Ugene_119U107769U108935U+U11_1	655351622	WP_028758489.1	63.57	387	0	534	hypothetical protein	Rhizobium sp. JGI 0001003-A11		
AS_1_contig_186Ugene_120U108935U109831U-U11_1	501559052	WP_012563548.1	72.05	297	6.00E-156	449	ATPase	Oligotropha carboxidovorans	K03061	PSMC2, RPT1; 26S proteasome regulatory subunit T1
AS_1_contig_186Ugene_121U109828U110337U-U11_1	499976491	WP_011657209.1	65.19	158	5.00E-64	205	hypothetical protein	Burkholderia ambifaria		
AS_1_contig_186Ugene_122U110340U111284U-U11_1	655351625	WP_028758492.1	71.97	314	3.00E-170	487	nucleotidyltransferase	Rhizobium sp. JGI 0001003-A11		
AS_1_contig_186Ugene_123U111430U111747U+U11_1	563528706	WP_023734944.1	66.02	103	1.00E-38	136	MULTISPECIES: XRE family transcriptional regulator	Mesorhizobium	K19449	#N/A
AS_1_contig_186Ugene_125U112688U112903U-U11_1	495419472	WP_008144169.1	33.33	57	0.006	43.1	hypothetical protein	Bradyrhizobium sp. YR681		
AS_1_contig_186Ugene_126U113084U114253U+U11_1	505279339	WP_015466441.1	46.85	397	3.00E-87	280	Porin 41 (Por41) precursor	Micavibrio aeruginosavorus		
AS_1_contig_186Ugene_131U118665U119204U-U11_1	518932209	WP_020088084.1	69.27	179	6.00E-80	246	hypothetical protein	Hyphomicrobium zavarzinii		
AS_1_contig_186Ugene_134U123868U125253U-U11_1	406953055	EKD82448.1	38.96	385	8.00E-83	273	hypothetical protein ACD_39C01288G0001	uncultured bacterium	K15725	czcC; cobalt-zinc-cadmium resistance protein CzcC
AS_1_contig_186Ugene_140U127835U128095U-U11_1	652473586	WP_026868281.1	41.94	62	2.00E-04	45.4	hypothetical protein	Hyphomicrobium zavarzinii		
AS_1_contig_186Ugene_146U131946U132482U-U11_1	563752376	WP_023786128.1	70.67	150	3.00E-77	239	hypothetical protein	Hyphomicrobium nitrativorans		
AS_1_contig_186Ugene_150U134466U134834U-U11_1	563752354	WP_023786124.1	47.83	115	3.00E-24	100	hypothetical protein	Hyphomicrobium nitrativorans		
AS_1_contig_186Ugene_154U136863U138293U-U11_1	523699683	WP_020817932.1	59.65	456	9.00E-174	508	hypothetical protein	Sphingobium chinhatense		
AS_1_contig_186Ugene_155U138492U139718U+U11_1	566044156	ETI63536.1	52.58	407	4.00E-135	405	hypothetical protein C100_12290	Sphingobium sp. C100		
AS_1_contig_186Ugene_156U140115U140942U-U11_1	563752326	WP_023786118.1	69.96	273	4.00E-139	406	replication protein RepB	Hyphomicrobium nitrativorans		
AS_1_contig_186Ugene_275U244859U245611U-U11_1	537815541	WP_020985612.1	34.17	240	3.00E-37	142	hypothetical protein	Leptospira kmetyi		
AS_1_contig_186Ugene_277U247028U247612U+U11_1	503716174	WP_013950250.1	64.58	192	4.00E-78	243	MULTISPECIES: DSBA oxidoreductase	Hyphomicrobium		
AS_1_contig_186Ugene_341U310831U311946U-U11_1	612167821	EZQ04458.1	68.31	366	1.00E-179	516	RNA methyltransferase	Azospirillum brasilense	K07444	ypsC; putative N6-adenine-specific DNA methylase [EC:2.1.1.-]
AS_1_contig_199Ugene_1U236U1213U-U11_1	655970751	WP_029013340.1	44.72	322	1.00E-86	275	hypothetical protein	Azospirillum irakense		
AS_1_contig_199Ugene_2U1291U2664U-U11_1	563752533	WP_023786156.1	40.06	337	1.00E-56	201	hypothetical protein	Hyphomicrobium nitrativorans		
AS_1_contig_199Ugene_3U2579U3106U-U11_1	563752522	WP_023786154.1	50.97	155	1.00E-43	152	hypothetical protein	Hyphomicrobium nitrativorans		
AS_1_contig_199Ugene_5U5182U5784U+U11_1	563752511	WP_023786152.1	31.47	197	8.00E-12	70.1	hypothetical protein	Hyphomicrobium nitrativorans		
AS_1_contig_199Ugene_9U6990U7154U-U11_1	563752487	WP_023786148.1	64.44	45	1.00E-11	63.2	hypothetical protein	Hyphomicrobium nitrativorans		
AS_1_contig_199Ugene_10U7151U7567U-U11_1	563752482	WP_023786147.1	42.62	122	1.00E-18	86.3	hypothetical protein	Hyphomicrobium nitrativorans		
AS_1_contig_199Ugene_16U11591U11860U+U11_1	488730067	WP_002653487.1	57.38	61	3.00E-15	74.7	hypothetical protein	Blastopirellula marina		
AS_1_contig_199Ugene_17U12186U14525U+U11_1	655033593	WP_028482384.1	37.88	396	2.00E-53	206	hypothetical protein	Nesiotobacter exalbescens		
AS_1_contig_199Ugene_18U14735U15004U+U11_1	521960716	WP_020472321.1	57.81	64	2.00E-16	77.4	hypothetical protein	Zavarzinella formosa		
AS_1_contig_199Ugene_19U15028U16968U+U11_1	499831451	WP_011512185.1	32.46	690	2.00E-47	187	complement C1q protein	Nitrobacter hamburgensis		

AS_1_contig_199Ugene_22U18255U18779U+U11_1	655339873	WP_028747966.1	42.35	170	1.00E-39	144	hypothetical protein	Rhizobium mesoamericanum		
AS_1_contig_199Ugene_25U20261U20893U+U11_1	654371016	KEC79963.1	66.3	184	2.00E-76	241	hypothetical protein SAMCCGM7_c2307	Sinorhizobium americanum CCGM7		
AS_1_contig_199Ugene_27U21623U22183U+U11_1	516613949	WP_017988792.1	71.91	178	3.00E-87	266	hypothetical protein	Rhizobium leguminosarum		
AS_1_contig_199Ugene_29U22915U23364U+U11_1	493380432	WP_006336698.1	71.23	146	2.00E-68	215	conserved membrane hypothetical protein	Mesorhizobium sp. STM 4661		
AS_1_contig_199Ugene_31U24614U25102U+U11_1	654694302	WP_028152851.1	45.33	150	5.00E-34	128	hypothetical protein	Bradyrhizobium japonicum		
AS_1_contig_199Ugene_32U25264U26205U+U11_1	490552537	WP_004417646.1	26.33	281	2.00E-19	95.1	hypothetical protein	Vibrio orientalis		
AS_1_contig_199Ugene_35U27319U27963U+U11_1	406965375	EKD91009.1	49.52	208	5.00E-65	211	hypothetical protein ACD_30C00052G0027	uncultured bacterium		
AS_1_contig_199Ugene_36U28171U28431U+U11_1	518928999	WP_020084874.1	45.71	70	3.00E-12	66.6	hypothetical protein	Hyphomicrobium zavarzinii		
AS_1_contig_199Ugene_37U28431U28733U+U11_1	552529545	WP_022994352.1	43.27	104	2.00E-16	78.6	plasmid maintenance protein CcdB	Alcanivorax sp. PN-3		
AS_1_contig_199Ugene_39U29141U30037U-U11_1	504960338	WP_015147440.1	56.12	294	1.00E-117	352	pyrophosphatase	Oscillatoria acuminata		
AS_1_contig_199Ugene_41U31816U32613U-U11_1	648440053	WP_026131804.1	62.1	248	7.00E-104	315	hypothetical protein	Pandoraea sp. B-6		
AS_1_contig_199Ugene_42U32606U33490U-U11_1	627787667	AHY56884.1	50.68	292	7.00E-95	294	hypothetical protein BJS_08152	Bradyrhizobium japonicum SEMIA 5079		
AS_1_contig_199Ugene_43U33487U33861U-U11_1	504704237	WP_014891339.1	60.98	123	6.00E-48	161	XRE family transcriptional regulator	Methylocystis sp. SC2		
AS_1_contig_199Ugene_44U33858U34169U-U11_1	611841516	EZO18029.1	43.43	99	5.00E-20	88.2	hypothetical protein AJ62_05574	Pseudomonas aeruginosa 3575		
AS_1_contig_199Ugene_45U34330U35526U-U11_1	651612044	WP_026606175.1	72.36	398	0	591	hypothetical protein	Methylocapsa acidiphila	K01154	hsdS; type I restriction enzyme, S subunit [EC:3.1.21.3]
AS_1_contig_199Ugene_48U39537U40046U-U11_1	563752376	WP_023786128.1	71.71	152	2.00E-76	237	hypothetical protein	Hyphomicrobium nitrativorans		
AS_1_contig_199Ugene_49U40335U42074U+U11_1	659014647	WP_029844917.1	47.56	574	0	536	hypothetical protein	Vibrio parahaemolyticus	K03529	smc; chromosome segregation protein
AS_1_contig_199Ugene_50U42058U43470U+U11_1	640614374	WP_025042439.1	49.68	475	7.00E-146	437	lipoprotein	Sulfitobacter sp. MM-124		
AS_1_contig_199Ugene_52U43749U44108U-U11_1	563752354	WP_023786124.1	51.72	116	7.00E-31	117	hypothetical protein	Hyphomicrobium nitrativorans		
AS_1_contig_199Ugene_56U45525U46010U+U11_1	512612027	WP_016456094.1	57.01	107	1.00E-29	118	hypothetical protein	Propionimicrobium lymphophilum		
AS_1_contig_199Ugene_56U45525U46010U+U11_1	512612027	WP_016456094.1	53.92	102	3.00E-26	109	hypothetical protein	Propionimicrobium lymphophilum		
AS_1_contig_199Ugene_56U45525U46010U+U11_1	512612027	WP_016456094.1	50.98	102	6.00E-23	100	hypothetical protein	Propionimicrobium lymphophilum		
AS_1_contig_199Ugene_56U45525U46010U+U11_1	512612027	WP_016456094.1	46.23	106	5.00E-20	92.8	hypothetical protein	Propionimicrobium lymphophilum		
AS_1_contig_199Ugene_56U45525U46010U+U11_1	512612027	WP_016456094.1	47.67	86	9.00E-14	75.1	hypothetical protein	Propionimicrobium lymphophilum		
AS_1_contig_199Ugene_56U45525U46010U+U11_1	512612027	WP_016456094.1	38.71	93	5.00E-07	56.2	hypothetical protein	Propionimicrobium lymphophilum		
AS_1_contig_199Ugene_59U47929U48246U-U11_1	563752330	WP_023786119.1	67.65	68	2.00E-23	95.9	hypothetical protein	Hyphomicrobium nitrativorans		
AS_1_contig_199Ugene_60U48623U49522U-U11_1	563752326	WP_023786118.1	69.57	276	5.00E-144	420	replication protein RepB	Hyphomicrobium nitrativorans		
AS_1_contig_199Ugene_61U49690U50151U-U11_1	503587829	WP_013821905.1	58.57	140	9.00E-47	160	DNA N-6-adenine-methyltransferase	Desulfotomaculum kuznetsovii		
AS_1_contig_358Ugene_1U189U1202U-U11_1	504350377	WP_014537479.1	39.47	304	3.00E-56	196	hypothetical protein	Ketogulonicigenium vulgare		
AS_1_contig_358Ugene_4U3467U4282U+U11_1	333798380	YP_004508653.1	33.59	256	7.00E-36	139	Metallophosphoesterase	Synechococcus phage S-CRM01		
AS_1_contig_358Ugene_5U4285U5598U-U11_1	563752533	WP_023786156.1	36.95	341	4.00E-56	199	hypothetical protein	Hyphomicrobium nitrativorans		
AS_1_contig_358Ugene_6U5618U6076U-U11_1	563752522	WP_023786154.1	51.88	133	3.00E-36	132	hypothetical protein	Hyphomicrobium nitrativorans		
AS_1_contig_358Ugene_8U7828U8388U+U11_1	563752511	WP_023786152.1	30.95	168	2.00E-09	62.8	hypothetical protein	Hyphomicrobium nitrativorans		
AS_1_contig_358Ugene_9U8498U9181U+U11_1	515117551	WP_016746562.1	42.33	215	1.00E-40	149	hypothetical protein	Sphingomonas wittichii		
AS_1_contig_358Ugene_10U9245U9454U-U11_1	563754561	WP_023786590.1	41.38	58	3.00E-04	44.3	hypothetical protein	Hyphomicrobium nitrativorans		
AS_1_contig_358Ugene_12U10113U10286U-U11_1	563752493	WP_023786149.1	50	60	9.00E-08	53.1	hypothetical protein	Hyphomicrobium nitrativorans		
AS_1_contig_358Ugene_14U10450U10632U-U11_1	563752487	WP_023786148.1	73.08	52	5.00E-16	74.7	hypothetical protein	Hyphomicrobium nitrativorans		
AS_1_contig_358Ugene_15U10629U10847U-U11_1	563752482	WP_023786147.1	74.65	71	1.00E-29	112	hypothetical protein	Hyphomicrobium nitrativorans		
AS_1_contig_358Ugene_17U12540U15599U+U11_1	640603213	WP_025031651.1	31.05	570	1.00E-44	185	hypothetical protein	Nitratireductor aquibiodomus		
AS_1_contig_358Ugene_25U23567U23797U-U11_1	497508892	WP_009823090.1	54	50	2.00E-05	51.2	conjugal transfer protein TraG	Sphingomonas sp. SKA58		
AS_1_contig_358Ugene_27U25478U25750U+U11_1	503266872	WP_013501533.1	88.51	87	6.00E-49	161	antitoxin	Rhodopseudomonas palustris		
AS_1_contig_358Ugene_30U26566U27234U+U11_1	630768426	KCZ50361.1	73.99	223	5.00E-113	338	hypothetical protein HY2_14135	Hyphomonas sp. T16B2		
AS_1_contig_358Ugene_34U29466U29969U-U11_1	503406109	WP_013640770.1	57.32	164	6.00E-57	187	hypothetical protein	Acidiphilium multivorum		
AS_1_contig_358Ugene_36U32155U32532U-U11_1	518932216	WP_020088091.1	76.23	122	1.00E-57	186	hypothetical protein	Hyphomicrobium zavarzinii		
AS_1_contig_358Ugene_38U33867U34079U-U11_1	511290607	WP_016350874.1	80.3	66	7.00E-26	104	NAD-dependent epimerase/dehydratase	Aeromonas hydrophila		
AS_1_contig_358Ugene_39U34311U35000U+U11_1	517119046	WP_018307864.1	77.97	227	2.00E-130	379	dimethyl sulfoxide reductase subunit C	Acetobacter aceti		
AS_1_contig_358Ugene_42U36883U37791U+U11_1	491456012	WP_005313793.1	64.29	294	2.00E-142	416	hypothetical protein	Acinetobacter sp. ANC 3880		
AS_1_contig_358Ugene_45U40587U41042U+U11_1	494331544	WP_007183159.1	60	150	3.00E-55	187	hypothetical protein	Hydrocarboniphaga effusa	K07006	K07006
AS_1_contig_358Ugene_46U41053U41553U-U11_1	495665468	WP_008390047.1	78.38	111	2.00E-59	193	hypothetical protein	Rhodovulum sp. PH10	K09022	yjgF; UPF0076 protein YjgF
AS_1_contig_358Ugene_49U45166U45420U-U11_1	490393161	WP_004271616.1	59.21	76	2.00E-23	95.5	XRE family transcriptional regulator	Azospirillum amazonense		
AS_1_contig_358Ugene_50U45534U45962U+U11_1	654693811	WP_028152381.1	29.1	134	8.00E-05	48.1	hypothetical protein	Bradyrhizobium japonicum		
AS_1_contig_358Ugene_52U46756U47355U-U11_1	630943932	KCZ90231.1	31.47	143	3.00E-14	77.8	hypothetical protein HJA_03351	Hyphomonas jannaschiana VP2		
AS_1_contig_358Ugene_58U50767U51297U-U11_1	497483635	WP_009797833.1	51.19	168	6.00E-53	177	thiamine pyrophosphate-requiring enzyme-like protein	Nitrobacter sp. Nb-311A		
AS_1_contig_358Ugene_61U55117U56136U-U11_1	495873867	WP_008598446.1	50.78	321	5.00E-99	307	iron dicitrate transport regulator FecR	Nitratireductor pacificus	K07165	fecR; transmembrane sensor
AS_1_contig_358Ugene_63U57351U57602U-U11_1	490569251	WP_004434271.1	92.31	78	5.00E-43	145	prevent-host-death protein	Sinorhizobium meliloti		
AS_1_contig_358Ugene_67U58640U60613U+U11_1	494879765	WP_007605817.1	50.24	625	0	640	hypothetical protein	Rhizobium sp. PDO1-076		
AS_1_contig_358Ugene_68U60636U60959U+U11_1	648547124	WP_026238875.1	43.81	105	4.00E-22	97.1	hypothetical protein	Rhizobium leguminosarum		
AS_1_contig_358Ugene_70U61703U62464U+U11_1	493658835	WP_006610224.1	54.63	227	2.00E-82	259	conserved hypothetical protein	Bradyrhizobium sp. ORS 285		
AS_1_contig_358Ugene_71U62479U63477U+U11_1	493658834	WP_006610223.1	55.45	321	6.00E-117	352	hypothetical protein	Bradyrhizobium sp. ORS 285		
AS_1_contig_358Ugene_72U63470U64117U+U11_1	493658833	WP_006610222.1	46.45	211	4.00E-47	165	hypothetical protein	Bradyrhizobium sp. ORS 285		
AS_1_contig_358Ugene_73U64119U64700U-U11_1	493658832	WP_006610221.1	64.77	193	5.00E-89	271	hypothetical protein	Bradyrhizobium sp. ORS 285		
AS_1_contig_358Ugene_74U64697U65527U-U11_1	493658831	WP_006610220.1	68.75	272	5.00E-141	410	hypothetical protein	Bradyrhizobium sp. ORS 285	K07057	K07057
AS_1_contig_358Ugene_75U65999U66409U-U11_1	550971321	WP_022719535.1	80.15	136	8.00E-75	230	NtrR protein	Rhodopseudomonas sp. B29	K18828	
AS_1_contig_358Ugene_76U66406U66669U-U11_1	489636905	WP_003541345.1	90.8	87	3.00E-50	164	DNA-binding protein	Rhizobium leguminosarum	K18829	
AS_1_contig_358Ugene_77U66948U68294U+U11_1	651617014	WP_026611130.1	54.59	436	2.00E-164	484	hypothetical protein	Methylocaldum szegediense		
AS_1_contig_358Ugene_78U68291U68905U+U11_1	500002105	WP_011682823.1	58.19	177	4.00E-68	218	hypothetical protein	Candidatus Solibacter usitatus		
AS_1_contig_358Ugene_79U68902U72345U+U11_1	491907909	WP_005664655.1	51.77	1132	0	1138	hypothetical protein	Massilia timonae		
AS_1_contig_358Ugene_80U72560U73609U+U11_1	496218135	WP_008932172.1	54.68	342	2.00E-121	368	hypothetical protein	Ectothiorhodospira sp. PHS-1		
AS_1_contig_358Ugene_81U73778U74062U+U11_1	494320025	WP_007178551.1	47.76	67	1.00E-08	57.4	hypothetical protein	Burkholderia sp. Ch1-1		
AS_1_contig_358Ugene_82U74144U74584U-U11_1	640371175	WP_024881481.1	84.62	143	2.00E-79	243	twitching motility protein PilT	Methylosinus sp. LW3	K07062	K07062
AS_1_contig_358Ugene_83U74588U74839U-U11_1	490569251	WP_004434271.1	97.44	78	2.00E-46	154	prevent-host-death protein	Sinorhizobium meliloti		
AS_1_contig_358Ugene_84U75305U76132U+U11_1	333798380	YP_004508653.1	32.06	262	6.00E-34	134	Metallophosphoesterase	Synechococcus phage S-CRM01		
AS_1_contig_358Ugene_85U76331U76783U-U11_1	515298513	WP_016843605.1	70.95	148	2.00E-66	211	hypothetical protein	Bradyrhizobium elkanii		
AS_1_contig_358Ugene_89U81177U81641U-U11_1	503713350	WP_013947426.1	57.69	104	2.00E-22	99	hypothetical protein	Hyphomicrobium sp. MC1		
AS_1_contig_358Ugene_93U85723U86709U-U11_1	502531933	WP_012814403.1	58.18	330	2.00E-125	380	outermembrane protein	Candidatus Methylomirabilis oxyfera		
AS_1_contig_358Ugene_94U87039U87314U-U11_1	503716346	WP_013950422.1	57.89	57	6.00E-18	80.9	MULTISPECIES: hypothetical protein	Hyphomicrobium		
AS_1_contig_358Ugene_98U89052U89576U-U11_1	518930862	WP_020086737.1	71.97	157	2.00E-64	206	hypothetical protein	Hyphomicrobium zavarzinii		
AS_1_contig_358Ugene_99U89846U90112U+U11_1	652473586	WP_026868281.1	80	65	7.00E-27	104	hypothetical protein	Hyphomicrobium zavarzinii		
AS_1_contig_358Ugene_105U95118U95681U+U11_1	494666751	WP_007424695.1	35.33	167	4.00E-14	79	hypothetical protein	Acidiphilium sp. PM		
AS_1_contig_358Ugene_108U97668U98798U+U11_1	555407520	WP_023263301.1	56.2	363	1.00E-128	389	type I restriction endonuclease subunit S	Cupriavidus sp. HPC(L)	K01154	hsdS; type I restriction enzyme, S subunit [EC:3.1.21.3]
AS_1_contig_358Ugene_130U126469U127212U+U11_1	552542146	WP_023000602.1	36.84	190	1.00E-21	99.4	MULTISPECIES: hypothetical protein	Labrenzia		
AS_1_contig_358Ugene_134U129370U130830U-U11_1	652473372	WP_026868067.1	56.99	465	0	541	peptidase M4	Hyphomicrobium zavarzinii		
AS_1_contig_358Ugene_143U137263U139536U-U11_1	518930466	WP_020086341.1	77.13	752	0	1190	hypothetical protein	Hyphomicrobium zavarzinii	K00029	MaeB; malate dehydrogenase (oxaloacetate-decarboxylating)(NADP+) [EC:1.1.1.40]
AS_1_contig_358Ugene_189U188552U189058U-U11_1	501585114	WP_012589416.1	50.62	162	2.00E-48	167	TetR family transcriptional regulator	Methylocella silvestris		
AS_1_contig_358Ugene_190U189249U190088U+U11_1	497421941	WP_009736139.1	77.34	278	5.00E-156	448	beta-lactamase	Bradyrhizobiaceae bacterium SG-6C	K18610	

AS_1_contig_358Ugene_205U207381U207767U+U11_1	550974165	WP_022722379.1	49.11	112	6.00E-24	99.8	hypothetical protein	Rhodopseudomonas sp. B29		
AS_1_contig_358Ugene_206U207727U209808U+U11_1	516955623	WP_018183099.1	36.22	693	9.00E-90	305	hypothetical protein	Kaistia granuli		
AS_1_contig_358Ugene_208U211364U211822U-U11_1	485735324	WP_001362611.1	39.22	102	6.00E-04	48.5	autotransporter	Escherichia coli		
AS_1_contig_460Ugene_45U46215U47534U+U11_1	518929497	WP_020085372.1	36.63	404	8.00E-72	244	hypothetical protein	Hyphomicrobium zavarzinii		
AS_1_contig_460Ugene_47U48965U49969U-U11_1	505301361	WP_015488463.1	25.51	341	2.00E-30	128	hypothetical protein	Thalassolituus oleivorans		
AS_1_contig_460Ugene_48U49981U50868U-U11_1	655911375	WP_028981596.1	44.06	286	5.00E-86	271	hypothetical protein	Sporocytophaga myxococcoides		
AS_1_contig_460Ugene_49U50872U51576U-U11_1	515878343	WP_017308926.1	38.6	171	1.00E-29	119	hypothetical protein	Fischerella sp. PCC 9339		
AS_1_contig_460Ugene_50U51602U52477U-U11_1	505022215	WP_015209317.1	41.94	279	1.00E-72	236	putative taurine catabolism dioxygenase	Cylindrospermum stagnale	K03119	tauD; taurine dioxygenase [EC:1.14.11.17]
AS_1_contig_460Ugene_51U52703U53674U+U11_1	495104016	WP_007828839.1	49.41	253	2.00E-79	256	membrane protein	Variovorax sp. CF313		
AS_1_contig_460Ugene_52U53671U54399U+U11_1	651247483	WP_026382540.1	65.25	236	3.00E-103	311	peptidase M50	Achromobacter xylosoxidans	K06402	spoIVFB; stage IV sporulation protein FB [EC:3.4.24.-]
AS_1_contig_460Ugene_60U62629U62823U+U11_1	655891377	WP_028970511.1	79.66	59	2.00E-25	99.4	membrane protein	Sphingomonas sp. URHD0057		
AS_1_contig_460Ugene_65U65914U66435U-U11_1	503713505	WP_013947581.1	68.67	150	3.00E-72	226	MULTISPECIES: hypothetical protein	Hyphomicrobium		
AS_1_contig_460Ugene_76U77217U77702U-U11_1	503715241	WP_013949317.1	69.8	149	4.00E-72	225	MULTISPECIES: cupin	Hyphomicrobium		
AS_1_contig_460Ugene_104U104829U105548U+U11_1	503713673	WP_013947749.1	66.09	233	4.00E-107	321	MULTISPECIES: membrane protein	Hyphomicrobium		
AS_1_contig_460Ugene_105U105560U106180U-U11_1	503713674	WP_013947750.1	67	203	2.00E-92	282	abortive infection protein	Hyphomicrobium sp. MC1		
AS_1_contig_460Ugene_113U114167U115243U-U11_1	503713683	WP_013947759.1	40.06	347	9.00E-65	220	hypothetical protein	Hyphomicrobium sp. MC1		
AS_1_contig_460Ugene_122U123569U124210U-U11_1	503713692	WP_013947768.1	70.44	203	3.00E-98	295	MULTISPECIES: hypothetical protein	Hyphomicrobium		
AS_1_contig_460Ugene_129U129564U129758U-U11_1	654369776	KEC78772.1	39.06	64	2.00E-04	44.7	glutamine synthetase	Sinorhizobium americanum CCGM7		
AS_1_contig_460Ugene_162U167449U168969U+U11_1	499904705	WP_011585439.1	22.87	341	2.00E-15	87	hypothetical protein	Cytophaga hutchinsonii		
AS_1_contig_460Ugene_177U184688U185020U+U11_1	501557679	WP_012562180.1	79.41	68	7.00E-32	117	hypothetical protein	Oligotropha carboxidovorans		
AS_1_contig_460Ugene_178U185020U185562U+U11_1	590081644	EXL07951.1	77.22	180	2.00E-101	302	DNA repair protein	Aquamicrobium defluvii		
AS_1_contig_460Ugene_183U191758U192399U+U11_1	503715739	WP_013949815.1	67.43	175	7.00E-75	236	SH3 type 3 domain-containing protein (fragment)	Hyphomicrobium sp. MC1		
AS_1_contig_460Ugene_187U195920U196666U+U11_1	640222779	WP_024827675.1	67.74	248	1.00E-124	366	membrane protein	Methylobacterium sp. EUR3 AL-11	K03676	grxC, GLRX, GLRX2; glutaredoxin 3
AS_1_contig_460Ugene_204U225889U226137U+U11_1	503712442	WP_013946518.1	69.84	63	9.00E-21	88.2	MULTISPECIES: hypothetical protein	Hyphomicrobium		
AS_1_contig_460Ugene_229U254505U254792U-U11_1	654633271	WP_028097369.1	57.32	82	6.00E-25	100	membrane protein	Dongia sp. URHE0060		
AS_1_contig_460Ugene_230U254789U255049U-U11_1	654633270	WP_028097368.1	66.67	84	6.00E-32	117	membrane protein	Dongia sp. URHE0060		
AS_1_contig_460Ugene_232U255632U256639U+U11_1	504703498	WP_014890600.1	44.26	305	2.00E-89	282	cAMP-binding protein, regulatory protein	Methylocystis sp. SC2		
AS_1_contig_460Ugene_233U256705U257538U+U11_1	504703497	WP_014890599.1	63.41	246	4.00E-111	333	Sterol desaturase family protein	Methylocystis sp. SC2	K07750	SC4MOL, ERG25; methylsterol monooxygenase [EC:1.14.13.72]
AS_1_contig_460Ugene_270U295082U295480U-U11_1	503713934	WP_013948010.1	26.37	91	0.003	43.1	hypothetical protein	Hyphomicrobium sp. MC1		
AS_1_contig_483Ugene_9U12995U13597U+U11_1	636788934	WP_024325058.1	31.1	164	5.00E-13	73.2	hypothetical protein	Sinorhizobium medicae		
AS_1_contig_483Ugene_10U13700U15193U+U11_1	495561665	WP_008286244.1	32.3	418	7.00E-55	205	putative protease	Hydrogenivirga sp. 128-5-R1-1	K09607	ina; immune inhibitor A [EC:3.4.24.-]
AS_1_contig_483Ugene_12U15689U18601U-U11_1	505479182	WP_015665723.1	38.89	234	1.00E-29	135	conserved exported hypothetical protein	Bradyrhizobium oligotrophicum		
AS_1_contig_483Ugene_14U19229U20062U-U11_1	503324421	WP_013559082.1	44.11	263	2.00E-63	211	hypothetical protein	Anaerolinea thermophila		
AS_1_contig_483Ugene_15U20321U21412U+U11_1	374850047	BAL53046.1	56.9	290	3.00E-108	331	hypothetical conserved protein	uncultured Chloroflexi bacterium		
AS_1_contig_483Ugene_16U21421U22104U+U11_1	497471711	WP_009785909.1	64.86	222	9.00E-107	318	acylneuraminate cytidylyltransferase	Lyngbya sp. PCC 8106		
AS_1_contig_483Ugene_19U23868U24734U-U11_1	503965950	WP_014199944.1	43.01	286	6.00E-65	217	gluconolactonase	Azospirillum brasilense		
AS_1_contig_483Ugene_20U26909U27754U+U11_1	630765248	KCZ47286.1	38.89	270	5.00E-47	169	hypothetical protein HY17_19015	Hyphomonas sp. CY54-11-8	K09690	ABC-2.LPSE.P; lipopolysaccharide transport system permease protein
AS_1_contig_483Ugene_22U29052U29720U+U11_1	589594346	EXI66412.1	49.1	222	2.00E-62	205	putative O-methyltransferase	Candidatus Accumulibacter sp. SK-11		
AS_1_contig_483Ugene_23U29853U30722U+U11_1	551667632	XP_005836576.1	24.78	230	2.00E-09	65.9	hypothetical protein GUITHDRAFT_162048	Guillardia theta CCMP2712		
AS_1_contig_483Ugene_27U35184U36215U+U11_1	639178013	WP_024517630.1	53.48	316	2.00E-110	337	hypothetical protein	Bradyrhizobium sp. Tv2a-2	K00721	DPM1; dolichol-phosphate mannosyltransferase [EC:2.4.1.83]
AS_1_contig_483Ugene_30U37710U38165U+U11_1	665921853	WP_031287904.1	36.07	122	7.00E-18	85.5	hypothetical protein	Pseudomonas alcaligenes		
AS_1_contig_483Ugene_34U43505U44887U+U11_1	652922836	WP_027176688.1	37.91	277	6.00E-56	198	hypothetical protein	Desulfovibrio aminophilus		
AS_1_contig_483Ugene_35U44884U45777U+U11_1	46241626	AAS83011.1	33.03	218	3.00E-22	103	glycosyl transferase-like protein	Azospirillum brasilense	K13683	wcaE; putative colanic acid biosynthesis glycosyltransferase [EC:2.4.-.-]
AS_1_contig_483Ugene_37U46190U46864U-U11_1	655258326	WP_028668835.1	30.77	208	4.00E-18	89	hypothetical protein	Runella zeae		
AS_1_contig_483Ugene_38U47108U47941U-U11_1	494372356	WP_007199038.1	44.44	81	3.00E-11	72	hypothetical protein	Hoeflea phototrophica		
AS_1_contig_483Ugene_40U48871U49470U+U11_1	504205796	WP_014392898.1	35.25	139	4.00E-24	104	hypothetical protein	Corallococcus coralloides		
AS_1_contig_1378Ugene_3U7935U8351U+U11_1	517759681	WP_018929889.1	29.93	137	9.00E-10	62.4	MULTISPECIES: hypothetical protein	Pseudomonas		
AS_1_contig_1378Ugene_4U8945U10054U+U11_1	496252847	WP_008966232.1	41.7	271	2.00E-53	201	exported hypothetical protein	Bradyrhizobium sp. STM 3809		
AS_1_contig_1645Ugene_1U111U1610U+U11_1	499831507	WP_011512241.1	77.56	499	0	822	transposase	Nitrobacter hamburgensis		
AS_1_contig_1645Ugene_2U1610U2338U+U11_1	639181096	WP_024520684.1	84.65	241	3.00E-155	445	transposase	Bradyrhizobium sp. Tv2a-2		
AS_1_contig_1645Ugene_3U3050U3307U-U11_1	651612538	WP_026606668.1	52.11	71	2.00E-14	72.4	hypothetical protein	Methylocapsa acidiphila		
AS_1_contig_5149Ugene_1U222U1577U-U11_1	493519282	WP_006473457.1	99.78	451	0	924	MULTISPECIES: transposase	Proteobacteria		
AS_1_contig_5270Ugene_2U851U1183U-U11_1	550949726	WP_022698121.1	61.32	106	3.00E-35	127	XRE family transcriptional regulator	Maricaulis sp. JL2009		
AS_1_contig_5270Ugene_3U1503U2816U+U11_1	497483909	WP_009798107.1	72.03	429	0	642	DNA methylase N-4	Nitrobacter sp. Nb-311A		
AS_1_contig_9773Ugene_1U371U1828U-U11_1	654685371	WP_028144162.1	42.7	459	2.00E-115	360	terminase	Bradyrhizobium japonicum		
AS_1_contig_9773Ugene_2U1843U2253U-U11_1	503714955	WP_013949031.1	47.24	127	5.00E-25	103	hypothetical protein	Hyphomicrobium sp. MC1		

Table S4. CDSs categorized into F2 in Figure 4.

Contig_gene_name	BLASTP			KEGG (KAAS)	
	gi	Refseq ID	BLASTP description	KO	KO description
CP002083-37	502979108	WP_013214084.1	hypothetical protein		
CP002083-86	502979157	WP_013214133.1	glyoxalase	K06996	K06996
CP002083-87	502979158	WP_013214134.1	peptidase M23		
CP002083-113	502979184	WP_013214160.1	hypothetical protein		
CP002083-123	502979194	WP_013214170.1	exonuclease		
CP002083-134	502979205	WP_013214181.1	hypothetical protein		
CP002083-135	502979206	WP_013214182.1	hypothetical protein		
CP002083-146	502979217	WP_013214193.1	Pathogenesis-related transcriptional factor and ERF protein		
CP002083-148	502979219	WP_013214195.1	hypothetical protein		
CP002083-149	502979220	WP_013214196.1	ethanolamine utilization protein EutD		
CP002083-150	502979221	WP_013214197.1	hypothetical protein		
CP002083-153	502979224	WP_013214200.1	terminase		
CP002083-154	502979225	WP_013214201.1	terminase		
CP002083-155	502979226	WP_013214202.1	hypothetical protein		
CP002083-156	502979227	WP_013214203.1	hypothetical protein		
CP002083-157	502979228	WP_013214204.1	hypothetical protein		
CP002083-158	502979229	WP_013214205.1	hypothetical protein		
CP002083-171	502979242	WP_013214218.1	hypothetical protein		
CP002083-195	502979266	WP_013214242.1	hypothetical protein		
CP002083-196	502979267	WP_013214243.1	membrane protein		
CP002083-220	502979291	WP_013214267.1	hypothetical protein		
CP002083-221	502979292	WP_013214268.1	hypothetical protein		
CP002083-229	502979300	WP_013214276.1	alpha/beta hydrolase	K06889	K06889
CP002083-260	502979331	WP_013214307.1	hypothetical protein		
CP002083-266	502979337	WP_013214313.1	hypothetical protein		
CP002083-284	502979355	WP_013214331.1	hypothetical protein		
CP002083-339	502979410	WP_013214386.1	general stress protein		
CP002083-340	502979411	WP_013214387.1	hypothetical protein		
CP002083-342	502979413	WP_013214389.1	biotin synthase		
CP002083-348	502979419	WP_013214395.1	hypothetical protein		
CP002083-350	502979421	WP_013214397.1	hypothetical protein		
CP002083-351	502979422	WP_013214398.1	N-6 DNA methylase		
CP002083-352	502979423	WP_013214399.1	hypothetical protein		
CP002083-363	502979434	WP_013214410.1	hypothetical protein		
CP002083-364	502979435	WP_013214411.1	hypothetical protein		
CP002083-399	502979470	WP_013214446.1	outer membrane autotransporter barrel domain-containing protein		
CP002083-423	502979494	WP_013214470.1	peptidase M14	K06987	K06987
CP002083-424	502979495	WP_013214471.1	hypothetical protein		
CP002083-426	502979497	WP_013214473.1	hypothetical protein		
CP002083-430	502979501	WP_013214477.1	ankyrin		
CP002083-487	502979558	WP_013214534.1	cobalt transporter		
CP002083-527	502979598	WP_013214574.1	hypothetical protein		
CP002083-536	502979605	WP_013214581.1	hypothetical protein		
CP002083-570	502979639	WP_013214615.1	hypothetical protein		
CP002083-585	502979654	WP_013214630.1	Host attachment protein		
CP002083-597	502979666	WP_013214642.1	hypothetical protein		
CP002083-598	502979667	WP_013214643.1	hypothetical protein		
CP002083-599	502979668	WP_013214644.1	cyanate hydratase	K01725	cynS; cyanate lyase [EC:4.2.1.104]
CP002083-605	502979674	WP_013214650.1	hypothetical protein		
CP002083-608	502979677	WP_013214653.1	hypothetical protein		
CP002083-647	502979716	WP_013214692.1	hypothetical protein		
CP002083-648	502979717	WP_013214693.1	hypothetical protein		
CP002083-649	502979718	WP_013214694.1	hypothetical protein		
CP002083-650	502979719	WP_013214695.1	hypothetical protein		
CP002083-651	502979720	WP_013214696.1	hypothetical protein		
CP002083-652	502979721	WP_013214697.1	hypothetical protein		
CP002083-653	502979722	WP_013214698.1	hypothetical protein		
CP002083-654	502979723	WP_013214699.1	hypothetical protein		
CP002083-655	502979724	WP_013214700.1	hypothetical protein		

CP002083-656	502979725	WP_013214701.1	hypothetical protein		
CP002083-657	502979726	WP_013214702.1	hypothetical protein		
CP002083-658	502979727	WP_013214703.1	hypothetical protein		
CP002083-659	502979728	WP_013214704.1	hypothetical protein		
CP002083-665	502979734	WP_013214710.1	sulfonate/nitrate transporter substrate-binding protein	K02051	ABC.SN.S; NitT/TauT family transport system substrate-binding protein
CP002083-670	502979739	WP_013214715.1	hypothetical protein		
CP002083-673	502979742	WP_013214718.1	hypothetical protein	K07112	K07112
CP002083-675	502979744	WP_013214720.1	radical SAM protein	K09711	K09711; hypothetical protein
CP002083-676	502979745	WP_013214721.1	geranylgeranyl reductase		
CP002083-677	502979746	WP_013214722.1	radical SAM protein	K01012	bioB; biotin synthase [EC:2.8.1.6]
CP002083-678	502979747	WP_013214723.1	biotin/lipoate A/B protein ligase	K03800	lplA; lipoate-protein ligase A [EC:2.7.7.63]
CP002083-679	502979748	WP_013214724.1	GMP synthase - glutamine amidotransferase domain-like protein		
CP002083-681	502979750	WP_013214726.1	heterodisulfide reductase subunit C	K03390	hdrC; heterodisulfide reductase subunit C [EC:1.8.98.1]
CP002083-682	502979751	WP_013214727.1	heterodisulfide reductase subunit B	K03389	hdrB; heterodisulfide reductase subunit B [EC:1.8.98.1]
CP002083-683	502979752	WP_013214728.1	pyridine nucleotide-disulfide oxidoreductase	K03388	hdrA; heterodisulfide reductase subunit A [EC:1.8.98.1]
CP002083-684	502979753	WP_013214729.1	hypothetical protein		
CP002083-685	502979754	WP_013214730.1	heterodisulfide reductase subunit C	K03390	hdrC; heterodisulfide reductase subunit C [EC:1.8.98.1]
CP002083-686	502979755	WP_013214731.1	disulfide reductase	K03389	hdrB; heterodisulfide reductase subunit B [EC:1.8.98.1]
CP002083-687	502979756	WP_013214732.1	hypothetical protein		
CP002083-690	502979759	WP_013214735.1	response regulator SirA	K04085	tusA, sirA; tRNA 2-thiouridine synthesizing protein A [EC:2.8.1.-]
CP002083-691	502979760	WP_013214736.1	hypothetical protein	K07112	K07112
CP002083-694	502979763	WP_013214739.1	hypothetical protein	K17223	soxX; sulfur-oxidizing protein SoxX
CP002083-695	502979764	WP_013214740.1	hypothetical protein	K17222	soxA1; sulfur-oxidizing protein SoxA
CP002083-698	502979767	WP_013214743.1	5'-nucleotidase	K17224	soxB1; sulfur-oxidizing protein SoxB
CP002083-701	502979769	WP_013214745.1	transposase		
CP002083-702	502979770	WP_013214746.1	MULTISPECIES: transcriptional regulator	K17071	dcmR; transcriptional repressor of dcmA and dcmR
CP002083-704	502979772	WP_013214748.1	hypothetical protein		
CP002083-705	502979773	WP_013214749.1	MULTISPECIES: hypothetical protein		
CP002083-706	502979496	WP_013214472.1	MULTISPECIES: transposase		
CP002083-713	502979780	WP_013214756.1	metal-dependent hydrolase	K06897	K06897; 7,8-dihydropterin-6-yl-methyl-4-(beta-D-ribofuranosyl)aminobenzene 5'-phosphate synthase [EC:2.5.1.10[illegible]
CP002083-715	502979782	WP_013214758.1	hypothetical protein		
CP002083-719	502979786	WP_013214762.1	hypothetical protein		
CP002083-720	502979787	WP_013214763.1	hypothetical protein		
CP002083-721	502979788	WP_013214764.1	NAD(P)H dehydrogenase (quinone)	K01118	acpD, azoR; FMN-dependent NADH-azoreductase [EC:1.7.-.-]
CP002083-723	502979790	WP_013214766.1	fatty acid desaturase	K00496	alkB1_2; alkane 1-monooxygenase [EC:1.14.15.3]
CP002083-727	502979794	WP_013214770.1	hypothetical protein		
CP002083-729	502979796	WP_013214772.1	hypothetical protein		
CP002083-734	502979801	WP_013214777.1	selenium-binding protein	K17285	SELENBP1; selenium-binding protein 1
CP002083-737	502979804	WP_013214780.1	hypothetical protein		
CP002083-740	502979807	WP_013214783.1	hypothetical protein		
CP002083-742	502979809	WP_013214785.1	hypothetical protein		
CP002083-754	502979821	WP_013214797.1	Crp/Fnr family transcriptional regulator		
CP002083-755	502979822	WP_013214798.1	hypothetical protein		
CP002083-756	502979823	WP_013214799.1	hypothetical protein		
CP002083-757	502979824	WP_013214800.1	hypothetical protein		
CP002083-759	502979826	WP_013214802.1	hypothetical protein		
CP002083-774	502979841	WP_013214817.1	acetoin:2,6-dichlorophenolindophenol oxidoreductase subunit alpha	K00161	PDHA, pdhA; pyruvate dehydrogenase E1 component subunit alpha [EC:1.2.4.1]
CP002083-780	502979847	WP_013214823.1	hypothetical protein		
CP002083-783	502979850	WP_013214826.1	hypothetical protein		
CP002083-794	502979861	WP_013214837.1	hypothetical protein		
CP002083-797	502979864	WP_013214840.1	ribonucleotide-diphosphate reductase subunit beta	K00526	E1.17.4.1B, nrdB, nrdF; ribonucleoside-diphosphate reductase beta chain [EC:1.17.4.1]
CP002083-800	502979867	WP_013214843.1	beta-lactamase		
CP002083-802	502979869	WP_013214845.1	signal peptide protein		
CP002083-810	502979877	WP_013214853.1	hypothetical protein		
CP002083-821	502979888	WP_013214864.1	hypothetical protein		
CP002083-823	502979890	WP_013214866.1	membrane protein		
CP002083-897	502979964	WP_013214940.1	HNH endonuclease		
CP002083-898	502979965	WP_013214941.1	hypothetical protein		
CP002083-900	502979967	WP_013214943.1	hypothetical protein		
CP002083-906	502979973	WP_013214949.1	RND transporter		
CP002083-908	502979975	WP_013214951.1	hypothetical protein		
CP002083-909	502979976	WP_013214952.1	hypothetical protein		
CP002083-917	502979984	WP_013214960.1	hypothetical protein		

CP002083-930	502979997	WP_013214973.1	hypothetical protein		
CP002083-932	502979999	WP_013214975.1	hypothetical protein		
CP002083-933	502980000	WP_013214976.1	signal peptide protein		
CP002083-935	502980002	WP_013214978.1	hypothetical protein		
CP002083-944	502980011	WP_013214987.1	hypothetical protein		
CP002083-985	502980052	WP_013215028.1	hypothetical protein		
CP002083-1036	502980103	WP_013215079.1	hypothetical protein		
CP002083-1066	502980133	WP_013215109.1	hypothetical protein		
CP002083-1093	502980160	WP_013215136.1	hypothetical protein		
CP002083-1094	502980161	WP_013215137.1	hypothetical protein		
CP002083-1095	502980162	WP_013215138.1	hypothetical protein		
CP002083-1143	502980210	WP_013215186.1	hypothetical protein		
CP002083-1146	502980213	WP_013215189.1	integrase family protein		
CP002083-1147	502980214	WP_013215190.1	hypothetical protein		
CP002083-1148	502980215	WP_013215191.1	hypothetical protein		
CP002083-1149	502980216	WP_013215192.1	hypothetical protein		
CP002083-1150	502980217	WP_013215193.1	restriction alleviation protein, Lar family		
CP002083-1151	502980218	WP_013215194.1	hypothetical protein		
CP002083-1152	502980219	WP_013215195.1	hypothetical protein		
CP002083-1153	502980220	WP_013215196.1	hypothetical protein		
CP002083-1154	502980221	WP_013215197.1	hypothetical protein		
CP002083-1155	502980222	WP_013215198.1	hypothetical protein		
CP002083-1157	502980224	WP_013215200.1	hypothetical protein		
CP002083-1158	502980225	WP_013215201.1	hypothetical protein		
CP002083-1159	502980226	WP_013215202.1	hypothetical protein		
CP002083-1160	502980227	WP_013215203.1	hypothetical protein		
CP002083-1162	502980229	WP_013215205.1	chemotaxis protein CheA		
CP002083-1163	502980230	WP_013215206.1	hypothetical protein		
CP002083-1165	502980232	WP_013215208.1	hypothetical protein		
CP002083-1166	502980233	WP_013215209.1	DEAD/DEAH box helicase		
CP002083-1167	502980234	WP_013215210.1	hypothetical protein		
CP002083-1168	502980235	WP_013215211.1	hypothetical protein		
CP002083-1169	502980236	WP_013215212.1	hypothetical protein		
CP002083-1170	502980237	WP_013215213.1	hypothetical protein		
CP002083-1171	502980238	WP_013215214.1	hypothetical protein		
CP002083-1172	502980239	WP_013215215.1	hypothetical protein		
CP002083-1173	502980240	WP_013215216.1	hypothetical protein		
CP002083-1174	502980241	WP_013215217.1	DNA methyltransferase	K06223	dam; DNA adenine methylase [EC:2.1.1.72]
CP002083-1175	502980242	WP_013215218.1	hypothetical protein		
CP002083-1176	502980243	WP_013215219.1	hypothetical protein		
CP002083-1177	502980244	WP_013215220.1	hypothetical protein		
CP002083-1178	502980245	WP_013215221.1	hypothetical protein		
CP002083-1179	502980246	WP_013215222.1	hypothetical protein		
CP002083-1181	502980248	WP_013215224.1	hypothetical protein		
CP002083-1182	502980249	WP_013215225.1	hypothetical protein		
CP002083-1183	502980250	WP_013215226.1	hypothetical protein		
CP002083-1184	502980251	WP_013215227.1	hypothetical protein		
CP002083-1185	502980252	WP_013215228.1	hypothetical protein		
CP002083-1186	502980253	WP_013215229.1	hypothetical protein		
CP002083-1187	502980254	WP_013215230.1	hypothetical protein		
CP002083-1188	502980255	WP_013215231.1	hypothetical protein		
CP002083-1189	502980256	WP_013215232.1	plasmid maintenance system antidote protein, XRE family		
CP002083-1190	502980257	WP_013215233.1	hypothetical protein		
CP002083-1191	502980258	WP_013215234.1	hypothetical protein		
CP002083-1192	502980259	WP_013215235.1	hypothetical protein		
CP002083-1193	502980260	WP_013215236.1	hypothetical protein		
CP002083-1194	502980261	WP_013215237.1	hypothetical protein		
CP002083-1195	502980262	WP_013215238.1	hypothetical protein		
CP002083-1196	502980263	WP_013215239.1	peptidoglycan-binding protein		
CP002083-1197	502980264	WP_013215240.1	hypothetical protein		
CP002083-1198	502980265	WP_013215241.1	hypothetical protein		
CP002083-1199	502980266	WP_013215242.1	hypothetical protein		
CP002083-1200	502980267	WP_013215243.1	hypothetical protein		

CP002083-1201	502980268	WP_013215244.1	terminase GpA
CP002083-1202	502980269	WP_013215245.1	hypothetical protein
CP002083-1203	502980270	WP_013215246.1	phage portal protein, lambda family
CP002083-1205	502980272	WP_013215248.1	hypothetical protein
CP002083-1206	502980273	WP_013215249.1	hypothetical protein
CP002083-1207	502980274	WP_013215250.1	hypothetical protein
CP002083-1208	502980275	WP_013215251.1	hypothetical protein
CP002083-1209	502980276	WP_013215252.1	hypothetical protein
CP002083-1210	502980277	WP_013215253.1	phage-like protein
CP002083-1211	502980278	WP_013215254.1	Mu tail sheath family protein
CP002083-1212	502980279	WP_013215255.1	hypothetical protein
CP002083-1213	502980280	WP_013215256.1	hypothetical protein
CP002083-1214	502980281	WP_013215257.1	phage tail tape measure protein, TP901 family
CP002083-1215	502980282	WP_013215258.1	DNA circulation family protein
CP002083-1216	502980283	WP_013215259.1	Mu P family protein
CP002083-1217	502980284	WP_013215260.1	Mu-like prophage protein GP45-like protein
CP002083-1218	502980285	WP_013215261.1	hypothetical protein
CP002083-1219	502980286	WP_013215262.1	GP46 family protein
CP002083-1220	502980287	WP_013215263.1	Baseplate J family protein
CP002083-1221	502980288	WP_013215264.1	hypothetical protein
CP002083-1222	502980289	WP_013215265.1	hypothetical protein
CP002083-1223	502980290	WP_013215266.1	hypothetical protein
CP002083-1224	502980291	WP_013215267.1	hypothetical protein
CP002083-1226	502980293	WP_013215269.1	hypothetical protein
CP002083-1227	502980294	WP_013215270.1	hypothetical protein
CP002083-1228	502980295	WP_013215271.1	hypothetical protein
CP002083-1229	502980296	WP_013215272.1	hypothetical protein
CP002083-1252	502980319	WP_013215295.1	hypothetical protein
CP002083-1285	502980352	WP_013215328.1	OsmC family protein
CP002083-1314	502980381	WP_013215357.1	AraC family transcriptional regulator
CP002083-1317	502980384	WP_013215360.1	hypothetical protein
CP002083-1318	502980385	WP_013215361.1	hypothetical protein
CP002083-1320	502980387	WP_013215363.1	hypothetical protein
CP002083-1321	502980388	WP_013215364.1	hypothetical protein
CP002083-1322	502980389	WP_013215365.1	hypothetical protein
CP002083-1323	502980390	WP_013215366.1	transcription factor jumonji
CP002083-1327	502980394	WP_013215370.1	hypothetical protein
CP002083-1330	502980397	WP_013215373.1	glycosyl transferase family 2
CP002083-1332	502980399	WP_013215375.1	hypothetical protein
CP002083-1333	502980400	WP_013215376.1	pectin lyase-like (virulence factor-like)
CP002083-1336	502980403	WP_013215379.1	glycosyl transferase family 1
CP002083-1337	502980404	WP_013215380.1	hypothetical protein
CP002083-1434	502980501	WP_013215477.1	hypothetical protein
CP002083-1473	502980540	WP_013215516.1	hypothetical protein
CP002083-1476	502980543	WP_013215519.1	chromosome partitioning protein ParB
CP002083-1488	502980555	WP_013215531.1	FAD-binding monooxygenase protein
CP002083-1510	502980577	WP_013215553.1	integrase family protein
CP002083-1511	502979177	WP_013214153.1	hypothetical protein
CP002083-1512	502979178	WP_013214154.1	hypothetical protein
CP002083-1513	502979179	WP_013214155.1	Fis family transcriptional regulator
CP002083-1514	502979180	WP_013214156.1	hypothetical protein
CP002083-1515	502979181	WP_013214157.1	hypothetical protein
CP002083-1516	502979182	WP_013214158.1	hypothetical protein
CP002083-1517	502979183	WP_013214159.1	hypothetical protein
CP002083-1518	502980578	WP_013215554.1	hypothetical protein
CP002083-1519	502979185	WP_013214161.1	hypothetical protein
CP002083-1520	502979186	WP_013214162.1	hypothetical protein
CP002083-1521	502979187	WP_013214163.1	hypothetical protein
CP002083-1522	502979188	WP_013214164.1	hypothetical protein
CP002083-1523	502979189	WP_013214165.1	hypothetical protein
CP002083-1524	502979190	WP_013214166.1	hypothetical protein
CP002083-1525	502979191	WP_013214167.1	hypothetical protein
CP002083-1526	502979192	WP_013214168.1	HNH endonuclease

CP002083-1527	502979193	WP_013214169.1	hypothetical protein		
CP002083-1528	502980579	WP_013215555.1	exonuclease		
CP002083-1529	502979195	WP_013214171.1	single-stranded DNA-binding protein		
CP002083-1530	502979196	WP_013214172.1	hypothetical protein		
CP002083-1531	502979197	WP_013214173.1	hypothetical protein		
CP002083-1532	502979198	WP_013214174.1	hypothetical protein		
CP002083-1533	502979199	WP_013214175.1	hypothetical protein		
CP002083-1534	502979200	WP_013214176.1	hypothetical protein		
CP002083-1535	502979201	WP_013214177.1	hypothetical protein		
CP002083-1536	502979202	WP_013214178.1	hypothetical protein		
CP002083-1537	502979203	WP_013214179.1	hypothetical protein		
CP002083-1538	502979204	WP_013214180.1	hypothetical protein		
CP002083-1539	502980580	WP_013215556.1	hypothetical protein		
CP002083-1540	502980581	WP_013215557.1	hypothetical protein		
CP002083-1541	502979207	WP_013214183.1	hypothetical protein		
CP002083-1542	502979208	WP_013214184.1	hypothetical protein		
CP002083-1543	502979209	WP_013214185.1	hypothetical protein		
CP002083-1544	502979210	WP_013214186.1	hypothetical protein		
CP002083-1546	502979212	WP_013214188.1	hypothetical protein		
CP002083-1547	502979213	WP_013214189.1	hypothetical protein		
CP002083-1548	502979214	WP_013214190.1	hypothetical protein		
CP002083-1549	502979215	WP_013214191.1	hypothetical protein		
CP002083-1550	502979216	WP_013214192.1	hypothetical protein	K07726	K07726; putative transcriptional regulator
CP002083-1551	502979218	WP_013214194.1	hypothetical protein		
CP002083-1552	502980582	WP_013215558.1	hypothetical protein		
CP002083-1553	502980583	WP_013215559.1	hypothetical protein		
CP002083-1554	502979222	WP_013214198.1	hypothetical protein		
CP002083-1555	502979223	WP_013214199.1	hypothetical protein		
CP002083-1557	502980585	WP_013215561.1	hypothetical protein		
CP002083-1558	502980586	WP_013215562.1	hypothetical protein		
CP002083-1559	502979230	WP_013214206.1	hypothetical protein		
CP002083-1560	502979231	WP_013214207.1	hypothetical protein		
CP002083-1561	502979232	WP_013214208.1	hypothetical protein		
CP002083-1562	502979233	WP_013214209.1	hypothetical protein		
CP002083-1563	502979234	WP_013214210.1	hypothetical protein		
CP002083-1564	502979235	WP_013214211.1	transporter		
CP002083-1565	502979236	WP_013214212.1	hypothetical protein		
CP002083-1566	502979237	WP_013214213.1	hypothetical protein		
CP002083-1567	502979238	WP_013214214.1	Fis family transcriptional regulator		
CP002083-1568	502979239	WP_013214215.1	hypothetical protein		
CP002083-1569	502979240	WP_013214216.1	hypothetical protein		
CP002083-1571	502980588	WP_013215564.1	hypothetical protein		
CP002083-1572	502979243	WP_013214219.1	hypothetical protein		
CP002083-1574	502979245	WP_013214221.1	hypothetical protein		
CP002083-1575	502979246	WP_013214222.1	Fis family transcriptional regulator		
CP002083-1576	502979247	WP_013214223.1	hypothetical protein		
CP002083-1577	502979248	WP_013214224.1	hypothetical protein		
CP002083-1578	502980589	WP_013215565.1	ankyrin		
CP002083-1579	502980590	WP_013215566.1	hypothetical protein		
CP002083-1643	502980654	WP_013215630.1	hypothetical protein		
CP002083-1714	502980725	WP_013215701.1	hypothetical protein		
CP002083-1715	502980726	WP_013215702.1	excisionase family DNA binding domain-containing protein		
CP002083-1717	502980728	WP_013215704.1	phage/plasmid primase, P4 family	K06919	K06919; putative DNA primase/helicase
CP002083-1721	502980732	WP_013215708.1	hypothetical protein		
CP002083-1727	502980738	WP_013215714.1	ABC transporter substrate-binding protein	K02016	ABC.FEV.S; iron complex transport system substrate-binding protein
CP002083-1731	502980742	WP_013215718.1	hypothetical protein		
CP002083-1732	502980743	WP_013215719.1	hypothetical protein		
CP002083-1733	502980744	WP_013215720.1	PadR family transcripitonal regulator	K10947	padR; PadR family transcriptional regulator, regulatory protein PadR
CP002083-1734	502980745	WP_013215721.1	cupin		
CP002083-1735	502980746	WP_013215722.1	hypothetical protein		
CP002083-1742	502980753	WP_013215729.1	AMP-binding domain-containing protein		
CP002083-1782	502980793	WP_013215769.1	hypothetical protein		
CP002083-1843	502980854	WP_013215830.1	molybdenum cofactor sulfurase		

CP002083-1870	502980881	WP_013215857.1	regulatory protein		
CP002083-1871	502980882	WP_013215858.1	hypothetical protein		
CP002083-1872	502980883	WP_013215859.1	RNA-binding protein		
CP002083-1873	502980884	WP_013215860.1	DNA-damage-inducible protein D	K14623	dinD; DNA-damage-inducible protein D
CP002083-1946	502980957	WP_013215933.1	hypothetical protein		
CP002083-1994	502981005	WP_013215981.1	hypothetical protein		
CP002083-2029	502981040	WP_013216016.1	acyl-CoA hydrolase		
CP002083-2030	502981041	WP_013216017.1	hypothetical protein		
CP002083-2031	502981042	WP_013216018.1	isocitrate lyase	K01637	E4.1.3.1, aceA; isocitrate lyase [EC:4.1.3.1]
CP002083-2032	502981043	WP_013216019.1	Cro/Cl family transcriptional regulator	K07110	K07110
CP002083-2033	502981044	WP_013216020.1	hypothetical protein		
CP002083-2035	502981046	WP_013216022.1	hypothetical protein		
CP002083-2036	502981047	WP_013216023.1	hypothetical protein		
CP002083-2037	502981048	WP_013216024.1	hypothetical protein		
CP002083-2038	502981049	WP_013216025.1	hypothetical protein		
CP002083-2039	502981050	WP_013216026.1	hypothetical protein		
CP002083-2042	502981053	WP_013216029.1	hypothetical protein	K05569	mnhE, mrpE; multicomponent Na+:H+ antiporter subunit E
CP002083-2093	502981104	WP_013216080.1	methyltransferase		
CP002083-2112	502981123	WP_013216099.1	arabinogalactan protein		
CP002083-2114	502981125	WP_013216101.1	hypothetical protein		
CP002083-2116	502981127	WP_013216103.1	hypothetical protein		
CP002083-2117	502981128	WP_013216104.1	hypothetical protein		
CP002083-2118	502981129	WP_013216105.1	general secretion pathway protein M	K02462	gspM; general secretion pathway protein M
CP002083-2119	502981130	WP_013216106.1	fimbrial assembly protein	K02461	gspL; general secretion pathway protein L
CP002083-2120	502981131	WP_013216107.1	hypothetical protein	K02460	gspK; general secretion pathway protein K
CP002083-2121	502981132	WP_013216108.1	hypothetical protein	K02459	gspJ; general secretion pathway protein J
CP002083-2122	502981133	WP_013216109.1	hypothetical protein	K02458	gspI; general secretion pathway protein I
CP002083-2123	502981134	WP_013216110.1	general secretion pathway protein H	K02457	gspH; general secretion pathway protein H
CP002083-2124	502981135	WP_013216111.1	general secretion pathway protein G	K02456	gspG; general secretion pathway protein G
CP002083-2125	502981136	WP_013216112.1	type II secretion protein F	K02455	gspF; general secretion pathway protein F
CP002083-2128	502981139	WP_013216115.1	peptidase A24A prepilin type IV	K02654	pilD, pppA; leader peptidase (prepilin peptidase) / N-methyltransferase [EC:3.4.23.43 2.1.1.-]
CP002083-2130	502981141	WP_013216117.1	carboxylesterase	K03929	pnbA; para-nitrobenzyl esterase [EC:3.1.1.-]
CP002083-2131	502981142	WP_013216118.1	carboxylesterase	K03929	pnbA; para-nitrobenzyl esterase [EC:3.1.1.-]
CP002083-2132	502981143	WP_013216119.1	hypothetical protein		
CP002083-2134	502981145	WP_013216121.1	hypothetical protein		
CP002083-2136	502981147	WP_013216123.1	hypothetical protein		
CP002083-2155	502981166	WP_013216142.1	hypothetical protein		
CP002083-2158	502981169	WP_013216145.1	hypothetical protein		
CP002083-2161	502981172	WP_013216148.1	hypothetical protein		
CP002083-2162	502981173	WP_013216149.1	hypothetical protein		
CP002083-2163	502981174	WP_013216150.1	hypothetical protein		
CP002083-2164	502981175	WP_013216151.1	hypothetical protein	K06193	phnA; phosphonoacetate hydrolase [EC:3.11.1.2]
CP002083-2166	502981177	WP_013216153.1	hypothetical protein		
CP002083-2186	502981197	WP_013216173.1	deoxyribodipyrimidine photo-lyase	K01669	E4.1.99.3, phrB; deoxyribodipyrimidine photo-lyase [EC:4.1.99.3]
CP002083-2226	502981237	WP_013216213.1	hypothetical protein		
CP002083-2300	502981311	WP_013216287.1	excinuclease ABC subunit C		
CP002083-2310	502981321	WP_013216297.1	arsenic transporter	K03325	TC.ACR3; arsenite transporter, ACR3 family
CP002083-2320	502981331	WP_013216307.1	hypothetical protein		
CP002083-2326	502981337	WP_013216313.1	hypothetical protein		
CP002083-2353	502981364	WP_013216340.1	osmoprotectant transporter permease		
CP002083-2427	502981438	WP_013216414.1	hypothetical protein		
CP002083-2447	502981458	WP_013216434.1	phosphoglycerate mutase		
CP002083-2453	502981464	WP_013216440.1	hypothetical protein		
CP002083-2471	502981482	WP_013216458.1	hypothetical protein		
CP002083-2530	502981541	WP_013216517.1	hypothetical protein		
CP002083-2540	502981551	WP_013216527.1	hypothetical protein		
CP002083-2565	502981576	WP_013216552.1	hypothetical protein	K09965	K09965; hypothetical protein
CP002083-2598	502981609	WP_013216585.1	hypothetical protein		
CP002083-2716	502981727	WP_013216703.1	cystathionine gamma-synthase		
CP002083-2764	502981775	WP_013216751.1	heme oxygenase	K07215	hemO; heme oxygenase
CP002083-2770	502981781	WP_013216757.1	hypothetical protein		
CP002083-2771	502981782	WP_013216758.1	hypothetical protein		
CP002083-2772	502981783	WP_013216759.1	isochorismatase hydrolase		

CP002083-2773	502981784	WP_013216760.1	phosphate-selective porin O and P		
CP002083-2775	502981786	WP_013216762.1	urease accessory protein UreD	K03190	ureD, ureH; urease accessory protein
CP002083-2777	502981788	WP_013216764.1	urease subunit alpha	K01428	ureC; urease subunit alpha [EC:3.5.1.5]
CP002083-2778	502981789	WP_013216765.1	urease subunit beta	K14048	ureAB; urease subunit gamma/beta [EC:3.5.1.5]
CP002083-2779	502981790	WP_013216766.1	urease accessory protein UreF	K03188	ureF; urease accessory protein
CP002083-2780	502981791	WP_013216767.1	urease accessory protein UreE	K03187	ureE; urease accessory protein
CP002083-2783	502981794	WP_013216770.1	urea ABC transporter permease	K11961	urtC; urea transport system permease protein
CP002083-2784	502981795	WP_013216771.1	ABC transporter permease	K11960	urtB; urea transport system permease protein
CP002083-2785	502981796	WP_013216772.1	ABC transporter substrate-binding protein	K11959	urtA; urea transport system substrate-binding protein
CP002083-2787	502981798	WP_013216774.1	amino acid ABC substrate-binding protein	K01999	livK; branched-chain amino acid transport system substrate-binding protein
CP002083-2798	502981809	WP_013216785.1	deaminase reductase		
CP002083-2800	502981811	WP_013216787.1	LuxR family transcriptional regulator		
CP002083-2801	502981812	WP_013216788.1	outer membrane autotransporter barrel domain-containing protein		
CP002083-2805	502981816	WP_013216792.1	hypothetical protein		
CP002083-2897	502981908	WP_013216884.1	hypothetical protein		
CP002083-2904	502981915	WP_013216891.1	hypothetical protein	K07005	K07005
CP002083-2913	502981924	WP_013216900.1	hypothetical protein		
CP002083-2917	502981928	WP_013216904.1	hypothetical protein		
CP002083-2921	502981932	WP_013216908.1	hypothetical protein		
CP002083-2922	502981933	WP_013216909.1	hypothetical protein		
CP002083-2923	502981934	WP_013216910.1	hypothetical protein		
CP002083-2924	502981935	WP_013216911.1	hypothetical protein		
CP002083-2926	502981937	WP_013216913.1	hypothetical protein		
CP002083-2927	502981938	WP_013216914.1	hypothetical protein		
CP002083-2928	502981939	WP_013216915.1	hypothetical protein		
CP002083-2929	502981940	WP_013216916.1	hypothetical protein		
CP002083-2930	502981941	WP_013216917.1	hypothetical protein		
CP002083-2931	502981942	WP_013216918.1	hypothetical protein		
CP002083-2932	502981943	WP_013216919.1	hypothetical protein		
CP002083-2933	502981944	WP_013216920.1	Phage tail tape measure protein, conserved region		
CP002083-2934	502981945	WP_013216921.1	hypothetical protein		
CP002083-2935	502981946	WP_013216922.1	hypothetical protein		
CP002083-2936	502981947	WP_013216923.1	hypothetical protein		
CP002083-2938	502981949	WP_013216925.1	phage protein, HK97 gp10 family		
CP002083-2939	502981950	WP_013216926.1	phage head-tail adaptor		
CP002083-2941	502981952	WP_013216928.1	hypothetical protein		
CP002083-2942	502981953	WP_013216929.1	hypothetical protein		
CP002083-2943	502981954	WP_013216930.1	hypothetical protein		
CP002083-2944	502981955	WP_013216931.1	hypothetical protein		
CP002083-2947	502981958	WP_013216934.1	hypothetical protein		
CP002083-2948	502981959	WP_013216935.1	hypothetical protein		
CP002083-2949	502981960	WP_013216936.1	hypothetical protein		
CP002083-2950	502981961	WP_013216937.1	phage portal protein, HK97 family		
CP002083-2951	502981962	WP_013216938.1	terminase		
CP002083-2952	502981963	WP_013216939.1	hypothetical protein		
CP002083-2953	502981964	WP_013216940.1	hypothetical protein		
CP002083-2954	502981965	WP_013216941.1	HNH nuclease		
CP002083-2955	502981966	WP_013216942.1	hypothetical protein		
CP002083-2956	502981967	WP_013216943.1	hypothetical protein		
CP002083-2957	502981968	WP_013216944.1	hypothetical protein		
CP002083-2958	502981969	WP_013216945.1	hypothetical protein		
CP002083-2959	502981970	WP_013216946.1	hypothetical protein		
CP002083-2961	502981972	WP_013216948.1	hypothetical protein		
CP002083-2962	502981973	WP_013216949.1	hypothetical protein		
CP002083-2963	502981974	WP_013216950.1	hypothetical protein		
CP002083-2964	502981975	WP_013216951.1	hypothetical protein		
CP002083-2965	502981976	WP_013216952.1	hypothetical protein		
CP002083-2966	502981977	WP_013216953.1	transcriptional regulator		
CP002083-2967	502981978	WP_013216954.1	hypothetical protein		
CP002083-2968	502981979	WP_013216955.1	hypothetical protein		
CP002083-2969	502981980	WP_013216956.1	hypothetical protein		
CP002083-2970	502981981	WP_013216957.1	hypothetical protein		
CP002083-2971	502981982	WP_013216958.1	hypothetical protein		

CP002083-2972	502981983	WP_013216959.1	hypothetical protein		
CP002083-2973	502981984	WP_013216960.1	hypothetical protein		
CP002083-2974	502981985	WP_013216961.1	hypothetical protein		
CP002083-2975	502981986	WP_013216962.1	hypothetical protein		
CP002083-2976	502981987	WP_013216963.1	hypothetical protein		
CP002083-2977	502981988	WP_013216964.1	DEAD/DEAH box helicase		
CP002083-2978	502981989	WP_013216965.1	hypothetical protein		
CP002083-2979	502981990	WP_013216966.1	hypothetical protein		
CP002083-2981	502981992	WP_013216968.1	hypothetical protein		
CP002083-2982	502981993	WP_013216969.1	DNA methyltransferase	K00558	DNMT1, dcm; DNA (cytosine-5)-methyltransferase 1 [EC:2.1.1.37]
CP002083-2983	502981994	WP_013216970.1	hypothetical protein		
CP002083-2984	502981995	WP_013216971.1	transmembrane and ubiquitin-like domain containing 1		
CP002083-2985	502981996	WP_013216972.1	hypothetical protein		
CP002083-2986	502981997	WP_013216973.1	hypothetical protein		
CP002083-2987	502981998	WP_013216974.1	hypothetical protein		
CP002083-2988	502981999	WP_013216975.1	hypothetical protein		
CP002083-2989	502982000	WP_013216976.1	hypothetical protein		
CP002083-2991	502982002	WP_013216978.1	hypothetical protein		
CP002083-2992	502982003	WP_013216979.1	hypothetical protein		
CP002083-2993	502982004	WP_013216980.1	XRE family transcriptional regulator		
CP002083-2995	502982006	WP_013216982.1	hypothetical protein		
CP002083-2996	502982007	WP_013216983.1	hypothetical protein		
CP002083-2997	502982008	WP_013216984.1	hypothetical protein		
CP002083-3092	502982103	WP_013217079.1	thyroglobulin		
CP002083-3175	502982186	WP_013217162.1	transmembrane protein		
CP002083-3182	502982193	WP_013217169.1	hypothetical protein		
CP002083-3183	502982194	WP_013217170.1	permease		
CP002083-3200	502982211	WP_013217187.1	hypothetical protein		
CP002083-3215	502982226	WP_013217202.1	hypothetical protein		
CP002083-3264	502982275	WP_013217251.1	beta-lactamase		
CP002083-3265	502982276	WP_013217252.1	AraC family transcriptional regulator		
CP002083-3388	502982399	WP_013217375.1	ribonuclease P	K03536	rnpA; ribonuclease P protein component [EC:3.1.26.5]
CP002083-3408	502982419	WP_013217395.1	peptidase C14 caspase catalytic subunit p20		
CP002083-3409	502982420	WP_013217396.1	hypothetical protein		
CP002083-3430	502982441	WP_013217417.1	hypothetical protein		
CP002083-3460	502982471	WP_013217447.1	hypothetical protein		
CP002083-3463	502982474	WP_013217450.1	3'-kinase	K04343	E2.7.1.72; streptomycin 6-kinase [EC:2.7.1.72]
CP002083-3485	502982496	WP_013217472.1	acyltransferase 3		
CP002083-3493	502982504	WP_013217480.1	hypothetical protein		

Table S5. CDSs categorized into F3 in Figure 4.

Contig_gene_name	BLASTP			KEGG (KAAS)	
	gi	Refseq ID	BLASTP description	KO	KO description
CP005587-17	505409147	WP_015596249.1	hypothetical protein		
CP005587-27	505409157	WP_015596259.1	hypothetical protein		
CP005587-33	505409163	WP_015596265.1	hypothetical protein		
CP005587-41	505409171	WP_015596273.1	hypothetical protein		
CP005587-47	505409177	WP_015596279.1	hypothetical protein		
CP005587-50	505409180	WP_015596282.1	hypothetical protein		
CP005587-76	505409206	WP_015596308.1	hypothetical protein		
CP005587-80	505409210	WP_015596312.1	hypothetical protein		
CP005587-90	505409220	WP_015596322.1	hypothetical protein		
CP005587-91	505409221	WP_015596323.1	hypothetical protein		
CP005587-92	505409222	WP_015596324.1	hypothetical protein		
CP005587-93	505409223	WP_015596325.1	hypothetical protein		
CP005587-96	505409226	WP_015596328.1	hypothetical protein		
CP005587-97	505409227	WP_015596329.1	hypothetical protein		
CP005587-99	505409229	WP_015596331.1	hypothetical protein		
CP005587-101	505409231	WP_015596333.1	hypothetical protein		
CP005587-102	505409232	WP_015596334.1	histidine kinase		
CP005587-105	505409235	WP_015596337.1	hypothetical protein		
CP005587-106	505409236	WP_015596338.1	hypothetical protein		
CP005587-107	505409237	WP_015596339.1	hypothetical protein		
CP005587-109	505409239	WP_015596341.1	hypothetical protein		
CP005587-111	505409241	WP_015596343.1	hypothetical protein		
CP005587-127	505409257	WP_015596359.1	hypothetical protein		
CP005587-129	505409259	WP_015596361.1	glycosyl transferase family 2	K19425	
CP005587-131	505409261	WP_015596363.1	hypothetical protein		
CP005587-132	505409262	WP_015596364.1	hypothetical protein		
CP005587-134	505409264	WP_015596366.1	hypothetical protein		
CP005587-136	505409266	WP_015596368.1	hypothetical protein		
CP005587-137	505409267	WP_015596369.1	PEBP family protein	K06910	K06910
CP005587-140	505409270	WP_015596372.1	hypothetical protein		
CP005587-141	505409271	WP_015596373.1	hypothetical protein		
CP005587-142	505409272	WP_015596374.1	transposase	K07486	K07486; transposase
CP005587-143	505409273	WP_015596375.1	hypothetical protein		
CP005587-145	505409275	WP_015596377.1	hypothetical protein	K04065	osmY; hyperosmotically inducible periplasmic protein
CP005587-146	505409276	WP_015596378.1	hypothetical protein		
CP005587-148	505409278	WP_015596380.1	hypothetical protein		
CP005587-150	505409280	WP_015596382.1	hypothetical protein		
CP005587-151	505409281	WP_015596383.1	hypothetical protein		
CP005587-153	505409283	WP_015596385.1	hypothetical protein		
CP005587-154	505409284	WP_015596386.1	nitroreductase		
CP005587-156	505409286	WP_015596388.1	hypothetical protein		
CP005587-158	505409288	WP_015596390.1	ribokinase-like domain-containing protein	K00852	rbsK, RBKS; ribokinase [EC:2.7.1.15]
CP005587-159	505409289	WP_015596391.1	hypothetical protein		
CP005587-160	505409290	WP_015596392.1	hypothetical protein		
CP005587-163	505409293	WP_015596395.1	hypothetical protein		
CP005587-176	505409306	WP_015596408.1	hypothetical protein		
CP005587-179	505409309	WP_015596411.1	glycosyltransferase		
CP005587-181	505409311	WP_015596413.1	putative glycosyltransferase protein		
CP005587-183	505409313	WP_015596415.1	hypothetical protein		
CP005587-189	505409319	WP_015596421.1	hypothetical protein		
CP005587-190	505409320	WP_015596422.1	hypothetical protein		
CP005587-191	505409321	WP_015596423.1	hypothetical protein		
CP005587-193	505409323	WP_015596425.1	cytochrome B561		

CP005587-196	505409326	WP_015596428.1	hypothetical protein		
CP005587-198	505409328	WP_015596430.1	hypothetical protein		
CP005587-200	505409330	WP_015596432.1	major facilitator transporter		
CP005587-201	505409331	WP_015596433.1	hypothetical protein		
CP005587-218	505409348	WP_015596450.1	hypothetical protein		
CP005587-227	505409357	WP_015596459.1	hypothetical protein		
CP005587-229	505409359	WP_015596461.1	hypothetical protein		
CP005587-251	505409381	WP_015596483.1	hypothetical protein		
CP005587-263	505409393	WP_015596495.1	hypothetical protein		
CP005587-280	505409410	WP_015596512.1	hypothetical protein		
CP005587-325	505409455	WP_015596557.1	hypothetical protein		
CP005587-331	505409461	WP_015596563.1	hypothetical protein		
CP005587-335	505409465	WP_015596567.1	hydrogenase-4 component E	K12140	hyfE; hydrogenase-4 component E [EC:1.-.-.-]
CP005587-336	505409466	WP_015596568.1	formate hydrogenlyase		
CP005587-339	505409469	WP_015596571.1	hypothetical protein		
CP005587-340	505409470	WP_015596572.1	cytochrome B561	K03620	hyaC; Ni/Fe-hydrogenase 1 B-type cytochrome subunit
CP005587-343	505409473	WP_015596575.1	hypothetical protein		
CP005587-344	505409474	WP_015596576.1	cation-transporting ATPase		
CP005587-346	505409476	WP_015596578.1	hypothetical protein		
CP005587-347	505409477	WP_015596579.1	cytochrome B561		
CP005587-354	505409484	WP_015596586.1	hypothetical protein		
CP005587-401	505409530	WP_015596632.1	hypothetical protein		
CP005587-402	505409531	WP_015596633.1	hypothetical protein		
CP005587-403	505409532	WP_015596634.1	membrane protein		
CP005587-407	505409536	WP_015596638.1	hypothetical protein		
CP005587-428	505409557	WP_015596659.1	acyltransferase 3		
CP005587-449	505409578	WP_015596680.1	hypothetical protein		
CP005587-459	505409588	WP_015596690.1	hypothetical protein		
CP005587-502	505409631	WP_015596733.1	hypothetical protein		
CP005587-509	505409638	WP_015596740.1	hypothetical protein		
CP005587-513	505409642	WP_015596744.1	cbb3-type cytochrome oxidase, subunit CcoQ		
CP005587-526	505409655	WP_015596757.1	hypothetical protein		
CP005587-530	505409659	WP_015596761.1	hypothetical protein		
CP005587-533	505409662	WP_015596764.1	hypothetical protein		
CP005587-544	505409673	WP_015596775.1	hypothetical protein		
CP005587-545	505409674	WP_015596776.1	hypothetical protein	K09003	K09003; hypothetical protein
CP005587-551	505409680	WP_015596782.1	sulfurtransferase		
CP005587-591	505409720	WP_015596822.1	hypothetical protein		
CP005587-595	505409724	WP_015596826.1	formate dehydrogenase-N subunit gamma	K00127	E1.2.1.2G; formate dehydrogenase, gamma subunit
CP005587-596	505409725	WP_015596827.1	protein fdhE	K02380	fdhE; FdhE protein
CP005587-597	505409726	WP_015596828.1	membrane protein	K07089	K07089
CP005587-600	505409729	WP_015596831.1	selenocysteine synthase	K01042	selA; L-seryl-tRNA(Ser) seleniumtransferase [EC:2.9.1.1]
CP005587-601	505409730	WP_015596832.1	hypothetical protein		
CP005587-603	505409732	WP_015596834.1	C4-dicarboxylate ABC transporter		
CP005587-604	505409733	WP_015596835.1	hypothetical protein		
CP005587-607	505409736	WP_015596838.1	hypothetical protein		
CP005587-615	505409744	WP_015596846.1	hypothetical protein		
CP005587-617	505409746	WP_015596848.1	GCN5 family N-acetyltransferase	K09181	yfiQ; hypothetical protein
CP005587-620	505409749	WP_015596851.1	molecular chaperone (small heat shock protein)	K13993	HSP20; HSP20 family protein
CP005587-624	505409753	WP_015596855.1	UspA domain-containing protein		
CP005587-627	505409756	WP_015596858.1	ABC transporter permease	K02066	ABC.X1.P; putative ABC transport system permease protein
CP005587-629	505409758	WP_015596860.1	hypothetical protein	K02067	ABC.X1.S; putative ABC transport system substrate-binding protein
CP005587-632	505409761	WP_015596863.1	hypothetical protein		
CP005587-634	505409763	WP_015596865.1	hypothetical protein		
CP005587-637	505409766	WP_015596868.1	membrane protein	K01992	ABC-2.P; ABC-2 type transport system permease protein
CP005587-649	505409778	WP_015596880.1	hypothetical protein		
CP005587-650	505409779	WP_015596881.1	small heat shock protein (HSP20) family		

CP005587-653	505409782	WP_015596884.1	hypothetical protein		
CP005587-654	505409783	WP_015596885.1	hypothetical protein		
CP005587-655	505409784	WP_015596886.1	hypothetical protein		
CP005587-676	505409805	WP_015596907.1	hypothetical protein		
CP005587-677	505409806	WP_015596908.1	hypothetical protein		
CP005587-680	505409809	WP_015596911.1	hypothetical protein		
CP005587-689	505409818	WP_015596920.1	hypothetical protein		
CP005587-694	505409823	WP_015596925.1	hypothetical protein		
CP005587-696	505409825	WP_015596927.1	hypothetical protein		
CP005587-699	505409828	WP_015596930.1	cupin		
CP005587-703	505409832	WP_015596934.1	hypothetical protein		
CP005587-705	505409834	WP_015596936.1	MFS transporter		
CP005587-706	505409835	WP_015596937.1	hypothetical protein		
CP005587-707	505409836	WP_015596938.1	hypothetical protein		
CP005587-708	505409837	WP_015596939.1	hypothetical protein		
CP005587-711	505409840	WP_015596942.1	hypothetical protein		
CP005587-713	505409842	WP_015596944.1	hypothetical protein		
CP005587-714	505409843	WP_015596945.1	cytochrome B561		
CP005587-715	505409844	WP_015596946.1	hypothetical protein		
CP005587-716	505409845	WP_015596947.1	hypothetical protein		
CP005587-717	505409846	WP_015596948.1	hypothetical protein		
CP005587-719	505409848	WP_015596950.1	Transposase IS3/IS911 family protein		
CP005587-722	505409851	WP_015596953.1	hypothetical protein		
CP005587-723	505409852	WP_015596954.1	hypothetical protein		
CP005587-725	505409854	WP_015596956.1	6-phosphogluconolactonase	K01057	PGLS, pgl, devB; 6-phosphogluconolactonase [EC:3.1.1.31]
CP005587-726	505409855	WP_015596957.1	hypothetical protein		
CP005587-727	505409856	WP_015596958.1	haloacid dehalogenase	K07024	K07024
CP005587-728	505409857	WP_015596959.1	glucose-6-phosphate 1-dehydrogenase	K00036	G6PD, zwf; glucose-6-phosphate 1-dehydrogenase [EC:1.1.1.49]
CP005587-734	505409863	WP_015596965.1	hypothetical protein		
CP005587-735	505409864	WP_015596966.1	hypothetical protein		
CP005587-736	505409865	WP_015596967.1	hypothetical protein		
CP005587-740	505409869	WP_015596971.1	hypothetical protein		
CP005587-744	505409873	WP_015596975.1	hypothetical protein		
CP005587-745	505409874	WP_015596976.1	hypothetical protein		
CP005587-748	505409877	WP_015596979.1	hypothetical protein		
CP005587-752	505409881	WP_015596983.1	hypothetical protein		
CP005587-753	505409882	WP_015596984.1	hypothetical protein		
CP005587-754	505409883	WP_015596985.1	adenosine monophosphate-protein transferase	K04095	fic; cell filamentation protein
CP005587-755	505409884	WP_015596986.1	hypothetical protein		
CP005587-756	505409885	WP_015596987.1	hypothetical protein		
CP005587-757	505409886	WP_015596988.1	hypothetical protein		
CP005587-758	505409887	WP_015596989.1	hypothetical protein		
CP005587-759	505409888	WP_015596990.1	hypothetical protein		
CP005587-760	505409889	WP_015596991.1	hypothetical protein		
CP005587-761	505409890	WP_015596992.1	hypothetical protein		
CP005587-762	505409891	WP_015596993.1	hypothetical protein		
CP005587-767	505409896	WP_015596998.1	hypothetical protein		
CP005587-768	505409897	WP_015596999.1	hypothetical protein		
CP005587-769	505409898	WP_015597000.1	hypothetical protein		
CP005587-770	505409899	WP_015597001.1	XRE family transcriptional regulator		
CP005587-771	505409900	WP_015597002.1	HigB toxin protein	K07334	higA; proteic killer suppression protein
CP005587-774	505409903	WP_015597005.1	hypothetical protein		
CP005587-775	505409904	WP_015597006.1	anti-sigma factor		
CP005587-778	505409907	WP_015597009.1	metallophosphoesterase		
CP005587-781	505409910	WP_015597012.1	hypothetical protein		
CP005587-782	505409911	WP_015597013.1	hypothetical protein		
CP005587-785	505409914	WP_015597016.1	transposase	K07487	K07487; transposase

CP005587-787	505409916	WP_015597018.1	hypothetical protein		
CP005587-788	505409917	WP_015597019.1	hypothetical protein		
CP005587-789	505409918	WP_015597020.1	hypothetical protein		
CP005587-791	505409920	WP_015597022.1	hypothetical protein		
CP005587-792	505409921	WP_015597023.1	hypothetical protein		
CP005587-797	505409926	WP_015597028.1	hypothetical protein		
CP005587-800	505409929	WP_015597031.1	hypothetical protein		
CP005587-809	505409938	WP_015597040.1	hypothetical protein		
CP005587-810	505409939	WP_015597041.1	hypothetical protein		
CP005587-811	505409940	WP_015597042.1	hypothetical protein		
CP005587-812	505409941	WP_015597043.1	hypothetical protein		
CP005587-816	505409945	WP_015597047.1	cytochrome B561		
CP005587-820	505409949	WP_015597051.1	hypothetical protein		
CP005587-822	505409951	WP_015597053.1	hypothetical protein		
CP005587-824	505409953	WP_015597055.1	hypothetical protein		
CP005587-825	505409954	WP_015597056.1	type I restriction-modification system specificity determinant	K01154	hsdS; type I restriction enzyme, S subunit [EC:3.1.21.3]
CP005587-827	505409956	WP_015597058.1	hypothetical protein		
CP005587-828	505409957	WP_015597059.1	SMC domain-containing protein		
CP005587-829	505409958	WP_015597060.1	hypothetical protein		
CP005587-830	505409959	WP_015597061.1	hypothetical protein		
CP005587-831	505409960	WP_015597062.1	hypothetical protein		
CP005587-832	505409961	WP_015597063.1	DNA primase	K02316	dnaG; DNA primase [EC:2.7.7.-]
CP005587-833	505409962	WP_015597064.1	hypothetical protein		
CP005587-834	505409963	WP_015597065.1	hypothetical protein		
CP005587-835	505409964	WP_015597066.1	hypothetical protein		
CP005587-836	505409965	WP_015597067.1	hypothetical protein		
CP005587-838	505409967	WP_015597069.1	hypothetical protein		
CP005587-839	505409968	WP_015597070.1	hypothetical protein		
CP005587-840	505409969	WP_015597071.1	hypothetical protein		
CP005587-841	505409970	WP_015597072.1	hypothetical protein		
CP005587-842	505409971	WP_015597073.1	hypothetical protein		
CP005587-843	505409972	WP_015597074.1	hypothetical protein		
CP005587-844	505409973	WP_015597075.1	hypothetical protein		
CP005587-845	505409974	WP_015597076.1	hypothetical protein		
CP005587-846	505409975	WP_015597077.1	hypothetical protein		
CP005587-847	505409976	WP_015597078.1	hypothetical protein		
CP005587-849	505409978	WP_015597080.1	hypothetical protein		
CP005587-850	505409979	WP_015597081.1	hypothetical protein		
CP005587-851	505409980	WP_015597082.1	hypothetical protein		
CP005587-853	505409982	WP_015597084.1	hypothetical protein		
CP005587-854	505409983	WP_015597085.1	hypothetical protein		
CP005587-855	505409984	WP_015597086.1	hypothetical protein		
CP005587-857	505409986	WP_015597088.1	hypothetical protein		
CP005587-858	505409987	WP_015597089.1	hypothetical protein		
CP005587-859	505409988	WP_015597090.1	hypothetical protein		
CP005587-860	505409989	WP_015597091.1	hypothetical protein		
CP005587-861	505409990	WP_015597092.1	hypothetical protein		
CP005587-862	505409991	WP_015597093.1	hypothetical protein		
CP005587-863	505409992	WP_015597094.1	hemolysin activation/secretion protein		
CP005587-864	505409993	WP_015597095.1	filamentous hemagglutinin outer membrane protein		
CP005587-865	505409994	WP_015597096.1	hypothetical protein		
CP005587-867	505409996	WP_015597098.1	putative addiction module toxin	K19157	
CP005587-868	505409997	WP_015597099.1	addiction module antitoxin RelB		
CP005587-869	505409998	WP_015597100.1	hypothetical protein		
CP005587-870	505409999	WP_015597101.1	hypothetical protein		
CP005587-871	505410000	WP_015597102.1	hypothetical protein		
CP005587-872	505410001	WP_015597103.1	protoporphyrinogen oxidase	K00230	hemG; menaquinone-dependent protoporphyrinogen oxidase [EC:1.3.5.3]

CP005587-873	505410002	WP_015597104.1	hypothetical protein		
CP005587-877	505410006	WP_015597108.1	transcriptional regulator		
CP005587-880	505410009	WP_015597111.1	hypothetical protein		
CP005587-881	505410010	WP_015597112.1	hypothetical protein		
CP005587-882	505410011	WP_015597113.1	hypothetical protein		
CP005587-883	505410012	WP_015597114.1	hypothetical protein		
CP005587-885	505410014	WP_015597116.1	hypothetical protein		
CP005587-886	505410015	WP_015597117.1	hypothetical protein		
CP005587-887	505410016	WP_015597118.1	hypothetical protein		
CP005587-888	505410017	WP_015597119.1	hypothetical protein		
CP005587-889	505410018	WP_015597120.1	hypothetical protein		
CP005587-890	505410019	WP_015597121.1	hypothetical protein		
CP005587-891	505410020	WP_015597122.1	adenosine monophosphate-protein transferase	K04095	fic; cell filamentation protein
CP005587-892	505410021	WP_015597123.1	hypothetical protein		
CP005587-895	505410024	WP_015597126.1	sulfide dehydrogenase, cytochrome subunit		
CP005587-896	505410025	WP_015597127.1	hypothetical protein		
CP005587-897	505410026	WP_015597128.1	hypothetical protein		
CP005587-904	505410033	WP_015597135.1	hypothetical protein		
CP005587-908	505410037	WP_015597139.1	hypothetical protein		
CP005587-912	505410041	WP_015597143.1	hypothetical protein		
CP005587-913	505410042	WP_015597144.1	MFS transporter		
CP005587-915	505410044	WP_015597146.1	hypothetical protein		
CP005587-916	505410045	WP_015597147.1	hypothetical protein		
CP005587-920	505410049	WP_015597151.1	hypothetical protein		
CP005587-921	505410050	WP_015597152.1	hypothetical protein		
CP005587-925	505410054	WP_015597156.1	hypothetical protein		
CP005587-929	505410058	WP_015597160.1	heavy metal transporter	K17686	copA, ATP7; Cu+-exporting ATPase [EC:3.6.3.54]
CP005587-931	505410060	WP_015597162.1	cation transporter	K17686	copA, ATP7; Cu+-exporting ATPase [EC:3.6.3.54]
CP005587-935	505410064	WP_015597166.1	hypothetical protein		
CP005587-938	505410067	WP_015597169.1	hypothetical protein		
CP005587-939	505410068	WP_015597170.1	putative carboxymuconolactone decarboxylase	K07486	K07486; transposase
CP005587-940	505410069	WP_015597171.1	hypothetical protein		
CP005587-941	505410070	WP_015597172.1	hypothetical protein		
CP005587-942	505410071	WP_015597173.1	hypothetical protein		
CP005587-943	505410072	WP_015597174.1	haloacid dehalogenase	K01560	E3.8.1.2; 2-haloacid dehalogenase [EC:3.8.1.2]
CP005587-944	505410073	WP_015597175.1	alkyl hydroperoxide reductase		
CP005587-945	505410074	WP_015597176.1	DoxX	K15977	K15977; putative oxidoreductase
CP005587-948	505410077	WP_015597179.1	hypothetical protein		
CP005587-950	505410079	WP_015597181.1	hypothetical protein		
CP005587-954	505410083	WP_015597185.1	alkylhydroperoxidase		
CP005587-955	505410084	WP_015597186.1	hypothetical protein	K09931	K09931; hypothetical protein
CP005587-958	505410087	WP_015597189.1	membrane protein		
CP005587-961	505410090	WP_015597192.1	radical SAM domain protein		
CP005587-962	505410091	WP_015597193.1	hypothetical protein		
CP005587-964	505410093	WP_015597195.1	conjugal transfer protein TraI	K13060	lasI, luxI; acyl homoserine lactone synthase [EC:2.3.1.184]
CP005587-966	505410095	WP_015597197.1	hypothetical protein		
CP005587-967	505410096	WP_015597198.1	conjugal transfer protein TrbI	K03195	virB10, lvhB10; type IV secretion system protein VirB10
CP005587-968	505410097	WP_015597199.1	conjugative transfer protein TrbG	K03204	virB9, lvhB9; type IV secretion system protein VirB9
CP005587-969	505410098	WP_015597200.1	conjugal transfer protein TrbF	K03200	virB5, lvhB5; type IV secretion system protein VirB5
CP005587-971	505410100	WP_015597202.1	hypothetical protein		
CP005587-977	505410106	WP_015597208.1	hypothetical protein		
CP005587-978	505410107	WP_015597209.1	hypothetical protein		
CP005587-984	505410113	WP_015597215.1	mercury transporter MerT	K08363	merT; mercuric ion transport protein
CP005587-985	505410114	WP_015597216.1	mercury transporter	K08364	merP; periplasmic mercuric ion binding protein
CP005587-986	505410115	WP_015597217.1	hypothetical protein		
CP005587-989	505410118	WP_015597220.1	hypothetical protein		
CP005587-992	505410121	WP_015597223.1	hypothetical protein		

CP005587-993	505410122	WP_015597224.1	hypothetical protein		
CP005587-997	505410126	WP_015597228.1	hypothetical protein		
CP005587-998	505410127	WP_015597229.1	hypothetical protein		
CP005587-999	505410128	WP_015597230.1	XRE family transcriptional regulator		
CP005587-1002	505410131	WP_015597233.1	hypothetical protein		
CP005587-1003	505410132	WP_015597234.1	hypothetical protein		
CP005587-1006	505410135	WP_015597237.1	methylase		
CP005587-1009	505410138	WP_015597240.1	hypothetical protein		
CP005587-1012	505410141	WP_015597243.1	hypothetical protein		
CP005587-1013	505410142	WP_015597244.1	hypothetical protein		
CP005587-1014	505410143	WP_015597245.1	hypothetical protein		
CP005587-1015	505410144	WP_015597246.1	DEAD/DEAH box helicase	K06877	K06877; DEAD/DEAH box helicase domain-containing protein
CP005587-1016	505410145	WP_015597247.1	Putative DNA or RNA helicase		
CP005587-1017	505410146	WP_015597248.1	hypothetical protein		
CP005587-1025	505410154	WP_015597256.1	hypothetical protein		
CP005587-1038	505410167	WP_015597269.1	hypothetical protein		
CP005587-1046	505410175	WP_015597277.1	hypothetical protein		
CP005587-1059	505410188	WP_015597290.1	methyltransferase		
CP005587-1070	505410199	WP_015597301.1	hypothetical protein		
CP005587-1071	505410200	WP_015597302.1	hypothetical protein		
CP005587-1075	505410204	WP_015597306.1	hypothetical protein		
CP005587-1080	505410209	WP_015597311.1	hypothetical protein		
CP005587-1085	505410214	WP_015597316.1	hypothetical protein		
CP005587-1089	505410218	WP_015597320.1	hypothetical protein		
CP005587-1090	505410219	WP_015597321.1	hypothetical protein		
CP005587-1098	505410227	WP_015597329.1	O-antigen acyltransferase		
CP005587-1099	505410228	WP_015597330.1	hypothetical protein		
CP005587-1104	505410233	WP_015597335.1	hypothetical protein		
CP005587-1105	505410234	WP_015597336.1	hypothetical protein		
CP005587-1107	505410236	WP_015597338.1	hypothetical protein		
CP005587-1110	505410239	WP_015597341.1	hypothetical protein		
CP005587-1126	505410255	WP_015597357.1	hypothetical protein		
CP005587-1130	505410259	WP_015597361.1	hypothetical protein		
CP005587-1137	505410266	WP_015597368.1	hypothetical protein		
CP005587-1139	505410268	WP_015597370.1	hypothetical protein		
CP005587-1140	505410269	WP_015597371.1	MarR family transcriptional regulator		
CP005587-1141	505410270	WP_015597372.1	hydrogenase (NiFe) small subunit HydA	K06282	E1.12.99.6S; hydrogenase small subunit [EC:1.12.99.6]
CP005587-1142	505410271	WP_015597373.1	hydrogenase 2 large subunit	K06281	E1.12.99.6L; hydrogenase large subunit [EC:1.12.99.6]
CP005587-1144	505410273	WP_015597375.1	Ni/Fe hydrogenase	K03620	hyaC; Ni/Fe-hydrogenase 1 B-type cytochrome subunit
CP005587-1145	505410274	WP_015597376.1	hydrogenase expression protein	K03605	hyaD; hydrogenase 1 maturation protease [EC:3.4.24.-]
CP005587-1146	505410275	WP_015597377.1	hydrogenase	K04653	hypC; hydrogenase expression/formation protein HypC
CP005587-1147	505410276	WP_015597378.1	hydrogenase-1 expression HyaE	K03619	hyaE; hydrogenase-1 operon protein HyaE
CP005587-1148	505410277	WP_015597379.1	hydrogenase expression protein HupH	K03618	hyaF; hydrogenase-1 operon protein HyaF
CP005587-1149	505410278	WP_015597380.1	rubredoxin	K03618	hyaF; hydrogenase-1 operon protein HyaF
CP005587-1150	505410279	WP_015597381.1	rubredoxin		
CP005587-1151	505410280	WP_015597382.1	Ni,Fe-hydrogenase I large subunit	K06281	E1.12.99.6L; hydrogenase large subunit [EC:1.12.99.6]
CP005587-1152	505410281	WP_015597383.1	hydrogenase nickel incorporation protein HypA	K04651	hypA, hybF; hydrogenase nickel incorporation protein HypA/HybF
CP005587-1154	505410283	WP_015597385.1	hydrogenase	K04653	hypC; hydrogenase expression/formation protein HypC
CP005587-1155	505410284	WP_015597386.1	hydrogenase formation protein HypD	K04654	hypD; hydrogenase expression/formation protein HypD
CP005587-1157	505410286	WP_015597388.1	hydrogenase maturation protein		
CP005587-1160	505410289	WP_015597391.1	NADH ubiquinone oxidoreductase 20 kDa subunit	K06282	E1.12.99.6S; hydrogenase small subunit [EC:1.12.99.6]
CP005587-1161	505410290	WP_015597392.1	nickel-dependent hydrogenase large subunit	K06281	E1.12.99.6L; hydrogenase large subunit [EC:1.12.99.6]
CP005587-1162	505410291	WP_015597393.1	carbamoyltransferase	K04656	hypF; hydrogenase maturation protein HypF
CP005587-1163	505410292	WP_015597394.1	hypothetical protein		
CP005587-1170	505410299	WP_015597401.1	hypothetical protein		
CP005587-1176	505410305	WP_015597407.1	hypothetical protein		
CP005587-1182	505410311	WP_015597413.1	hypothetical protein		

CP005587-1217	505410346	WP_015597448.1	hypothetical protein
CP005587-1230	505410359	WP_015597461.1	hypothetical protein
CP005587-1231	505410360	WP_015597462.1	hypothetical protein
CP005587-1235	505410364	WP_015597466.1	hypothetical protein
CP005587-1243	505410371	WP_015597473.1	hypothetical protein
CP005587-1247	505410375	WP_015597477.1	hypothetical protein
CP005587-1248	505410376	WP_015597478.1	hypothetical protein
CP005587-1277	505410404	WP_015597506.1	hypothetical protein
CP005587-1281	505410408	WP_015597510.1	hypothetical protein
CP005587-1284	505410411	WP_015597513.1	hypothetical protein
CP005587-1295	505410422	WP_015597524.1	hypothetical protein
CP005587-1300	505410427	WP_015597529.1	hypothetical protein
CP005587-1305	505410432	WP_015597534.1	hypothetical protein
CP005587-1308	505410435	WP_015597537.1	hypothetical protein
CP005587-1326	505410453	WP_015597555.1	hypothetical protein
CP005587-1327	505410454	WP_015597556.1	hypothetical protein
CP005587-1328	505410455	WP_015597557.1	hypothetical protein
CP005587-1329	505410456	WP_015597558.1	hypothetical protein
CP005587-1330	505410457	WP_015597559.1	hypothetical protein
CP005587-1333	505410460	WP_015597562.1	hypothetical protein
CP005587-1339	505410466	WP_015597568.1	hypothetical protein
CP005587-1372	505410499	WP_015597601.1	hypothetical protein
CP005587-1387	505410514	WP_015597616.1	hypothetical protein
CP005587-1394	505410521	WP_015597623.1	hypothetical protein
CP005587-1398	505410525	WP_015597627.1	aminopeptidase
CP005587-1399	505410526	WP_015597628.1	SAM-dependent methyltransferase
CP005587-1401	505410528	WP_015597630.1	GlcNAc-PI de-N-acetylase
CP005587-1404	505410531	WP_015597633.1	hypothetical protein
CP005587-1405	505410532	WP_015597634.1	cholera toxin secretion EpsM protein
CP005587-1419	505410546	WP_015597648.1	hypothetical protein
CP005587-1452	505410579	WP_015597681.1	hypothetical protein
CP005587-1467	505410594	WP_015597696.1	hypothetical protein
CP005587-1474	505410601	WP_015597703.1	hypothetical protein
CP005587-1505	505410632	WP_015597734.1	hypothetical protein
CP005587-1521	505410648	WP_015597750.1	hypothetical protein
CP005587-1528	505410655	WP_015597757.1	hypothetical protein
CP005587-1550	505410677	WP_015597779.1	hypothetical protein
CP005587-1570	505410696	WP_015597798.1	hypothetical protein
CP005587-1572	505410698	WP_015597800.1	hypothetical protein
CP005587-1595	505410720	WP_015597822.1	hypothetical protein
CP005587-1599	505410724	WP_015597826.1	hypothetical protein
CP005587-1600	505410725	WP_015597827.1	hypothetical protein
CP005587-1601	505410726	WP_015597828.1	hypothetical protein
CP005587-1602	505410727	WP_015597829.1	hypothetical protein
CP005587-1609	505410734	WP_015597836.1	hypothetical protein
CP005587-1611	505410736	WP_015597838.1	hypothetical protein
CP005587-1622	505410747	WP_015597849.1	hypothetical protein
CP005587-1624	505410749	WP_015597851.1	hypothetical protein
CP005587-1634	505410759	WP_015597861.1	hypothetical protein
CP005587-1645	505410770	WP_015597872.1	hypothetical protein
CP005587-1660	505410785	WP_015597887.1	hypothetical protein
CP005587-1667	505410792	WP_015597894.1	hypothetical protein
CP005587-1683	505410807	WP_015597909.1	hypothetical protein
CP005587-1699	505410823	WP_015597925.1	hypothetical protein
CP005587-1718	505410842	WP_015597944.1	hypothetical protein
CP005587-1790	505410913	WP_015598015.1	hypothetical protein
CP005587-1793	505410916	WP_015598018.1	hypothetical protein

CP005587-1797	505410920	WP_015598022.1	hypothetical protein		
CP005587-1827	505410950	WP_015598052.1	hypothetical protein		
CP005587-1830	505410953	WP_015598055.1	hypothetical protein		
CP005587-1851	505410974	WP_015598076.1	Na/Pi-cotransporter II-like protein		
CP005587-1860	505410983	WP_015598085.1	hypothetical protein		
CP005587-1896	505411019	WP_015598121.1	hypothetical protein		
CP005587-1936	505411059	WP_015598161.1	hypothetical protein		
CP005587-1954	505411077	WP_015598179.1	hypothetical protein		
CP005587-1956	505411079	WP_015598181.1	hypothetical protein		
CP005587-1960	505411083	WP_015598185.1	hypothetical protein		
CP005587-1962	505411085	WP_015598187.1	universal stress protein		
CP005587-1963	505411086	WP_015598188.1	hypothetical protein		
CP005587-1964	505411087	WP_015598189.1	hypothetical protein		
CP005587-1965	505411088	WP_015598190.1	hypothetical protein		
CP005587-1968	505411091	WP_015598193.1	hypothetical protein		
CP005587-1975	505411098	WP_015598200.1	hypothetical protein		
CP005587-1983	505411106	WP_015598208.1	hypothetical protein		
CP005587-1984	505411107	WP_015598209.1	hypothetical protein		
CP005587-2018	505411138	WP_015598240.1	hypothetical protein		
CP005587-2021	505411141	WP_015598243.1	hypothetical protein		
CP005587-2029	505411149	WP_015598251.1	hypothetical protein		
CP005587-2046	505411164	WP_015598266.1	hypothetical protein		
CP005587-2048	505411166	WP_015598268.1	hypothetical protein		
CP005587-2051	505411169	WP_015598271.1	hypothetical protein		
CP005587-2064	505411182	WP_015598284.1	hypothetical protein		
CP005587-2066	505411184	WP_015598286.1	hypothetical protein		
CP005587-2067	505411185	WP_015598287.1	hypothetical protein		
CP005587-2068	505411186	WP_015598288.1	hypothetical protein		
CP005587-2069	505411187	WP_015598289.1	hypothetical protein		
CP005587-2080	505411198	WP_015598300.1	PadR family transcriptional regulator		
CP005587-2083	505411201	WP_015598303.1	hypothetical protein		
CP005587-2093	505411211	WP_015598313.1	hypothetical protein		
CP005587-2094	505411212	WP_015598314.1	hypothetical protein		
CP005587-2096	505411214	WP_015598316.1	hypothetical protein		
CP005587-2116	505411234	WP_015598336.1	hypothetical protein		
CP005587-2120	505411238	WP_015598340.1	carboxypeptidase C (cathepsin A)	K01288	KEX1; carboxypeptidase D [EC:3.4.16.6]
CP005587-2121	505411239	WP_015598341.1	hypothetical protein		
CP005587-2124	505411241	WP_015598343.1	hypothetical protein		
CP005587-2125	505411242	WP_015598344.1	hypothetical protein		
CP005587-2126	505411243	WP_015598345.1	hypothetical protein		
CP005587-2127	505411244	WP_015598346.1	hypothetical protein		
CP005587-2129	505411246	WP_015598348.1	hypothetical protein		
CP005587-2131	505411248	WP_015598350.1	hypothetical protein		
CP005587-2132	505411249	WP_015598351.1	membrane protein		
CP005587-2134	505411251	WP_015598353.1	hypothetical protein		
CP005587-2135	505411252	WP_015598354.1	hypothetical protein		
CP005587-2136	505411253	WP_015598355.1	hypothetical protein		
CP005587-2139	505411256	WP_015598358.1	hypothetical protein		
CP005587-2140	505411257	WP_015598359.1	hypothetical protein		
CP005587-2141	505411258	WP_015598360.1	sulfurtransferase		
CP005587-2144	505411261	WP_015598363.1	hypothetical protein		
CP005587-2145	505411262	WP_015598364.1	hypothetical protein		
CP005587-2146	505411263	WP_015598365.1	ParA family protein		
CP005587-2147	505411264	WP_015598366.1	hypothetical protein		
CP005587-2152	505411269	WP_015598371.1	hypothetical protein		
CP005587-2154	505411271	WP_015598373.1	hypothetical protein		
CP005587-2168	505411285	WP_015598387.1	hypothetical protein		

CP005587-2183	505411300	WP_015598402.1	hypothetical protein		
CP005587-2185	505411302	WP_015598404.1	hypothetical protein		
CP005587-2190	505411307	WP_015598409.1	hypothetical protein		
CP005587-2213	505411330	WP_015598432.1	hypothetical protein		
CP005587-2222	505411339	WP_015598441.1	hypothetical protein		
CP005587-2225	505411342	WP_015598444.1	hypothetical protein		
CP005587-2239	505411356	WP_015598458.1	hypothetical protein	K07275	ompW; outer membrane protein
CP005587-2243	505411360	WP_015598462.1	hypothetical protein		
CP005587-2253	505410952	WP_015598054.1	phage protein		
CP005587-2255	505411370	WP_015598472.1	hypothetical protein		
CP005587-2257	505411372	WP_015598474.1	hypothetical protein		
CP005587-2258	505411373	WP_015598475.1	retron-type reverse transcriptase		
CP005587-2259	505411374	WP_015598476.1	hypothetical protein		
CP005587-2265	505411380	WP_015598482.1	hypothetical protein		
CP005587-2268	505411383	WP_015598485.1	hypothetical protein		
CP005587-2269	505411384	WP_015598486.1	hypothetical protein		
CP005587-2284	505411399	WP_015598501.1	hypothetical protein		
CP005587-2296	505411411	WP_015598513.1	hypothetical protein		
CP005587-2318	505411433	WP_015598535.1	hypothetical protein		
CP005587-2330	505411445	WP_015598547.1	hypothetical protein		
CP005587-2348	505411463	WP_015598565.1	hypothetical protein		
CP005587-2353	505411468	WP_015598570.1	hypothetical protein		
CP005587-2358	505411473	WP_015598575.1	hypothetical protein		
CP005587-2361	505411476	WP_015598578.1	hypothetical protein		
CP005587-2374	505411489	WP_015598591.1	hypothetical protein		
CP005587-2412	505411527	WP_015598629.1	hypothetical protein		
CP005587-2414	505411529	WP_015598631.1	hypothetical protein		
CP005587-2416	505411531	WP_015598633.1	hypothetical protein		
CP005587-2417	505411532	WP_015598634.1	GTPase RsgA	K06949	rsgA, engC; ribosome biogenesis GTPase [EC:3.6.1.-]
CP005587-2420	505411535	WP_015598637.1	hypothetical protein		
CP005587-2421	505411536	WP_015598638.1	hypothetical protein		
CP005587-2422	505411537	WP_015598639.1	hypothetical protein		
CP005587-2423	505411538	WP_015598640.1	hypothetical protein		
CP005587-2424	505411539	WP_015598641.1	hypothetical protein		
CP005587-2425	505411540	WP_015598642.1	hypothetical protein		
CP005587-2427	505411542	WP_015598644.1	hypothetical protein		
CP005587-2429	505411544	WP_015598646.1	hypothetical protein		
CP005587-2430	505411545	WP_015598647.1	hypothetical protein		
CP005587-2432	505411547	WP_015598649.1	hypothetical protein		
CP005587-2434	505411549	WP_015598651.1	hypothetical protein		
CP005587-2436	505411551	WP_015598653.1	hypothetical protein		
CP005587-2437	505411552	WP_015598654.1	hypothetical protein		
CP005587-2440	505411555	WP_015598657.1	hypothetical protein		
CP005587-2441	505411556	WP_015598658.1	hypothetical protein		
CP005587-2442	505411557	WP_015598659.1	hypothetical protein		
CP005587-2444	505411559	WP_015598661.1	hypothetical protein		
CP005587-2450	505411565	WP_015598667.1	hypothetical protein		
CP005587-2451	505411566	WP_015598668.1	cytochrome C		
CP005587-2454	505411569	WP_015598671.1	hypothetical protein		
CP005587-2455	505411570	WP_015598672.1	hypothetical protein		
CP005587-2456	505411571	WP_015598673.1	hypothetical protein		
CP005587-2457	505411572	WP_015598674.1	hypothetical protein		
CP005587-2459	505411574	WP_015598676.1	cupin		
CP005587-2463	505411578	WP_015598680.1	hypothetical protein		
CP005587-2465	505411580	WP_015598682.1	hypothetical protein		
CP005587-2481	505411592	WP_015598694.1	hypothetical protein		
CP005587-2482	505411593	WP_015598695.1	CopG/Arc/MetJ family transcriptional regulator		

CP005587-2483	505411594	WP_015598696.1	plasmid stabilization system protein		
CP005587-2484	505411595	WP_015598697.1	hypothetical protein		
CP005587-2485	505411596	WP_015598698.1	hypothetical protein		
CP005587-2486	505411597	WP_015598699.1	transcriptional regulator		
CP005587-2488	505411599	WP_015598701.1	organic radical activating enzyme family protein	K04068	nrdG; anaerobic ribonucleoside-triphosphate reductase activating protein [EC:1.97.1.4]
CP005587-2489	505411600	WP_015598702.1	serine/threonine protein kinase		
CP005587-2490	505411601	WP_015598703.1	hypothetical protein		
CP005587-2491	505411602	WP_015598704.1	hypothetical protein		
CP005587-2492	505411603	WP_015598705.1	hypothetical protein		
CP005587-2494	505411605	WP_015598707.1	phage-related minor tail protein		
CP005587-2518	505411629	WP_015598731.1	hypothetical protein		
CP005587-2534	505411645	WP_015598747.1	hypothetical protein		
CP005587-2537	505411648	WP_015598750.1	hypothetical protein		
CP005587-2566	505411677	WP_015598779.1	hypothetical protein		
CP005587-2567	505411678	WP_015598780.1	hypothetical protein		
CP005587-2568	505411679	WP_015598781.1	hypothetical protein		
CP005587-2576	505411687	WP_015598789.1	hypothetical protein		
CP005587-2580	505411691	WP_015598793.1	glycosyltransferase		
CP005587-2586	505411697	WP_015598799.1	hypothetical protein		
CP005587-2610	505411721	WP_015598823.1	hypothetical protein		
CP005587-2623	505411734	WP_015598836.1	hypothetical protein		
CP005587-2657	505411768	WP_015598870.1	hypothetical protein		
CP005587-2674	505411785	WP_015598887.1	hypothetical protein		
CP005587-2678	505411789	WP_015598891.1	hypothetical protein		
CP005587-2684	505411794	WP_015598896.1	hypothetical protein		
CP005587-2695	505411804	WP_015598906.1	hypothetical protein		
CP005587-2743	505411852	WP_015598954.1	hypothetical protein		
CP005587-2752	505411861	WP_015598963.1	hypothetical protein		
CP005587-2767	505411876	WP_015598978.1	hypothetical protein		
CP005587-2777	505411886	WP_015598988.1	hypothetical protein		
CP005587-2812	505411921	WP_015599023.1	hypothetical protein		
CP005587-2821	505411930	WP_015599032.1	hypothetical protein		
CP005587-2833	505411942	WP_015599044.1	hypothetical protein		
CP005587-2837	505411946	WP_015599048.1	hypothetical protein		
CP005587-2843	505411952	WP_015599054.1	hypothetical protein		
CP005587-2851	505411959	WP_015599061.1	hypothetical protein		
CP005587-2870	505411978	WP_015599080.1	membrane protein		
CP005587-2880	505411988	WP_015599090.1	hypothetical protein		
CP005587-2890	505411998	WP_015599100.1	hypothetical protein		
CP005587-2925	505412033	WP_015599135.1	hypothetical protein		
CP005587-2926	505412034	WP_015599136.1	hypothetical protein		
CP005587-2932	505412040	WP_015599142.1	hypothetical protein		
CP005587-2933	505412041	WP_015599143.1	hypothetical protein		
CP005587-2934	505412042	WP_015599144.1	hypothetical protein		
CP005587-2939	505412047	WP_015599149.1	membrane protein		
CP005587-2940	505412048	WP_015599150.1	fusaric acid resistance protein		
CP005587-2941	505412049	WP_015599151.1	hypothetical protein		
CP005587-2942	505412050	WP_015599152.1	hypothetical protein		
CP005587-2944	505412052	WP_015599154.1	hypothetical protein		
CP005587-2970	505412078	WP_015599180.1	hypothetical protein		
CP005587-2971	505412079	WP_015599181.1	hypothetical protein		
CP005587-3014	505412122	WP_015599224.1	hypothetical protein		
CP005587-3020	505412128	WP_015599230.1	hypothetical protein		
CP005587-3024	505412132	WP_015599234.1	hypothetical protein		
CP005587-3100	505412207	WP_015599309.1	hypothetical protein		
CP005587-3114	505412221	WP_015599323.1	hypothetical protein		
CP005587-3115	505412222	WP_015599324.1	hypothetical protein		

CP005587-3117	505412224	WP_015599326.1	hypothetical protein		
CP005587-3118	505412225	WP_015599327.1	hypothetical protein		
CP005587-3119	505412226	WP_015599328.1	hypothetical protein		
CP005587-3120	505412227	WP_015599329.1	hypothetical protein		
CP005587-3121	505412228	WP_015599330.1	hypothetical protein		
CP005587-3128	505412235	WP_015599337.1	glyoxalase/bleomycin resistance protein/dioxygenase		
CP005587-3129	505412236	WP_015599338.1	hypothetical protein		
CP005587-3142	505412249	WP_015599351.1	hypothetical protein		
CP005587-3158	505412265	WP_015599367.1	TetR family transcriptional regulator		
CP005587-3159	505412266	WP_015599368.1	TetR family transcriptional regulator	K16137	nemR; TetR/AcrR family transcriptional regulator, transcriptional repressor for nem operor
CP005587-3165	505412272	WP_015599374.1	hypothetical protein		
CP005587-3197	505412304	WP_015599406.1	hypothetical protein		
CP005587-3233	505412340	WP_015599442.1	hypothetical protein		
CP005587-3238	505412345	WP_015599447.1	hypothetical protein		
CP005587-3246	505412353	WP_015599455.1	hypothetical protein		
CP005587-3249	505412356	WP_015599458.1	hypothetical protein		
CP005587-3253	505412360	WP_015599462.1	hypothetical protein		
CP005587-3293	505412400	WP_015599502.1	hypothetical protein		
CP005587-3322	505412429	WP_015599531.1	hypothetical protein		
CP005587-3325	505412432	WP_015599534.1	hypothetical protein		
CP005587-3326	505412433	WP_015599535.1	hypothetical protein		
CP005587-3327	505412434	WP_015599536.1	hypothetical protein		
CP005587-3328	505412435	WP_015599537.1	hypothetical protein		
CP005587-3332	505412439	WP_015599541.1	hypothetical protein		
CP005587-3344	505412451	WP_015599553.1	hypothetical protein		
CP005587-3346	505412453	WP_015599555.1	hypothetical protein		
CP005587-3350	505412457	WP_015599559.1	hypothetical protein		
CP005587-3352	505412459	WP_015599561.1	hypothetical protein		
CP005587-3353	505412460	WP_015599562.1	hypothetical protein		
CP005587-3354	505412461	WP_015599563.1	prevent-host-death family protein		
CP005587-3355	505412462	WP_015599564.1	hypothetical protein		
CP005587-3357	505412464	WP_015599566.1	hypothetical protein		
CP005587-3358	505412465	WP_015599567.1	hypothetical protein		
CP005587-3392	505412499	WP_015599601.1	hypothetical protein		
CP005587-3420	505412527	WP_015599629.1	hypothetical protein		
CP005587-3421	505412528	WP_015599630.1	hypothetical protein		
CP005587-3422	505412529	WP_015599631.1	IS1 transposase		
CP005587-3459	505412566	WP_015599668.1	cupin		
CP005587-3487	505412594	WP_015599696.1	hypothetical protein		
CP005587-3492	505412599	WP_015599701.1	hypothetical protein		
CP005587-3522	505412629	WP_015599731.1	hypothetical protein		
CP005587-3523	505412630	WP_015599732.1	hypothetical protein		
CP005587-3524	505412631	WP_015599733.1	hypothetical protein		
CP005587-3525	505412632	WP_015599734.1	hypothetical protein		
CP005587-3528	505412635	WP_015599737.1	exodeoxyribonuclease I		
CP005587-3529	505412636	WP_015599738.1	hypothetical protein		
CP005587-3530	505412637	WP_015599739.1	hypothetical protein		
CP005587-3531	505412638	WP_015599740.1	hypothetical protein		
CP005587-3533	505412640	WP_015599742.1	hypothetical protein		
CP005587-3534	505412641	WP_015599743.1	hypothetical protein		
CP005587-3535	505412642	WP_015599744.1	hypothetical protein		
CP005587-3537	505412644	WP_015599746.1	membrane protein	K09771	TC.SMR3; small multidrug resistance family-3 protein
CP005587-3538	505412645	WP_015599747.1	TatD-related deoxyribonuclease		
CP005587-3541	505412648	WP_015599750.1	hypothetical protein		
CP005587-3542	505412649	WP_015599751.1	hypothetical protein		
CP005587-3545	505412652	WP_015599754.1	hypothetical protein		
CP005587-3547	505412654	WP_015599756.1	phosphoesterase PA-phosphatase-like protein	K19302	

CP005587-3548	505412655	WP_015599757.1	ChrB domain-containing protein		
CP005587-3549	505412656	WP_015599758.1	undecaprenyl-diphosphatase	K19302	
CP005587-3553	505412660	WP_015599762.1	multidrug transporter		
CP005587-3558	505412665	WP_015599767.1	conjugal transfer protein TraI	K13060	lasI, luxI; acyl homoserine lactone synthase [EC:2.3.1.184]
CP005587-3560	505412667	WP_015599769.1	hypothetical protein		
CP005587-3561	505412668	WP_015599770.1	conjugal transfer protein TrbI	K03195	virB10, lvhB10; type IV secretion system protein VirB10
CP005587-3562	505412669	WP_015599771.1	conjugative transfer protein TrbG	K03204	virB9, lvhB9; type IV secretion system protein VirB9
CP005587-3563	505412670	WP_015599772.1	conjugal transfer protein TrbF	K03200	virB5, lvhB5; type IV secretion system protein VirB5
CP005587-3565	505412672	WP_015599774.1	hypothetical protein		
CP005587-3573	505412680	WP_015599782.1	sodium/hydrogen exchanger		
CP005587-3575	505412682	WP_015599784.1	hypothetical protein		
CP005587-3576	505412683	WP_015599785.1	lytic transglycosylase		
CP005587-3578	505412685	WP_015599787.1	hypothetical protein		
CP005587-3579	505412686	WP_015599788.1	hypothetical protein		
CP005587-3583	505412690	WP_015599792.1	hypothetical protein		
CP005587-3584	505412691	WP_015599793.1	hypothetical protein		
CP005587-3585	505412692	WP_015599794.1	hypothetical protein		
CP005587-3587	505412693	WP_015599795.1	RpiR family transcriptional regulator	K19337	
CP005587-3588	505412694	WP_015599796.1	membrane protein		
CP005587-3590	505412696	WP_015599798.1	hypothetical protein		
CP005587-3591	505412697	WP_015599799.1	phosphoesterase	K19302	
CP005587-3594	505412700	WP_015599802.1	hypothetical protein		
CP005587-3595	505412701	WP_015599803.1	hypothetical protein		
CP005587-3597	505412703	WP_015599805.1	hypothetical protein		
CP005587-3599	505412705	WP_015599807.1	methylase/helicase		
CP005587-3600	505412706	WP_015599808.1	hypothetical protein		
CP005587-3601	505412707	WP_015599809.1	hypothetical protein		
CP005587-3602	505412708	WP_015599810.1	hypothetical protein		
CP005587-3603	505412709	WP_015599811.1	hypothetical protein		
CP005587-3604	505412710	WP_015599812.1	hypothetical protein		
CP005587-3605	505412711	WP_015599813.1	hypothetical protein		
CP005587-3606	505412712	WP_015599814.1	hypothetical protein		
CP005587-3611	505412717	WP_015599819.1	hypothetical protein		
CP005587-3612	505412718	WP_015599820.1	hypothetical protein		
CP005587-3613	505412719	WP_015599821.1	hypothetical protein		
CP005587-3615	505412721	WP_015599823.1	NTPase KAP		
CP005587-3616	505412722	WP_015599824.1	hypothetical protein		
CP005587-3617	505412723	WP_015599825.1	hypothetical protein		
CP005587-3638	505412744	WP_015599846.1	hypothetical protein		
CP005587-3644	505412750	WP_015599852.1	hypothetical protein		
CP005587-3673	505412779	WP_015599881.1	hypothetical protein		
CP005587-3674	505412780	WP_015599882.1	hypothetical protein		
CP005587-3675	505412781	WP_015599883.1	50S ribosomal protein L34	K02914	RP-L34, MRPL34, rpmH; large subunit ribosomal protein L34
CP005587-3678	505412784	WP_015599886.1	hypothetical protein		
CP005587-3679	505412785	WP_015599887.1	hypothetical protein		
CP005587-3680	505412786	WP_015599888.1	hypothetical protein		
CP005587-3683	505412789	WP_015599891.1	serine protease	K07403	nfeD; membrane-bound serine protease (ClpP class)
CP005587-3690	505412796	WP_015599898.1	hypothetical protein		
CP005587-3694	505412800	WP_015599902.1	hypothetical protein		
CP005587-3698	505412804	WP_015599906.1	hypothetical protein		
CP005587-3701	505412807	WP_015599909.1	hypothetical protein		
CP005587-3705	505412811	WP_015599913.1	hypothetical protein		
CP005587-3706	505412812	WP_015599914.1	hypothetical protein		
CP005587-3707	505412813	WP_015599915.1	hypothetical protein		
CP005587-3710	505412816	WP_015599918.1	hypothetical protein		
CP005587-3711	505412817	WP_015599919.1	coagulation factor 5/8 type domain-containing protein		
CP005587-3713	505412819	WP_015599921.1	hypothetical protein		

CP005587-3714	505412820	WP_015599922.1	hypothetical protein	
CP005587-3715	505412821	WP_015599923.1	hypothetical protein	
CP005587-3716	505412822	WP_015599924.1	hypothetical protein	
CP005587-3717	505412823	WP_015599925.1	hypothetical protein	
CP005587-3719	505412825	WP_015599927.1	hypothetical protein	
CP005587-3720	505412826	WP_015599928.1	hypothetical protein	
CP005587-3721	505412827	WP_015599929.1	hypothetical protein	
CP005587-3722	505412828	WP_015599930.1	hypothetical protein	
CP005587-3724	505412830	WP_015599932.1	hypothetical protein	
CP005587-3725	505412831	WP_015599933.1	hypothetical protein	
CP005587-3726	505412832	WP_015599934.1	hypothetical protein	
CP005587-3729	505412835	WP_015599937.1	hypothetical protein	
CP005587-3730	505412836	WP_015599938.1	hypothetical protein	
CP005587-3731	505412837	WP_015599939.1	hypothetical protein	
CP005587-3732	505412838	WP_015599940.1	hypothetical protein	
CP005587-3733	505412839	WP_015599941.1	hypothetical protein	
CP005587-3734	505412840	WP_015599942.1	hypothetical protein	
CP005587-3735	505412841	WP_015599943.1	hypothetical protein	
CP005587-3736	505412842	WP_015599944.1	hypothetical protein	
CP005587-3737	505412843	WP_015599945.1	hypothetical protein	
CP005587-3756	505412862	WP_015599964.1	hypothetical protein	
CP005587-3757	505412863	WP_015599965.1	hypothetical protein	
CP005587-3760	505412866	WP_015599968.1	hypothetical protein	
CP005587-3761	505412867	WP_015599969.1	hypothetical protein	
CP005587-3829	505412935	WP_015600037.1	prolyl-tRNA synthetase	K19055
CP005587-3832	505412938	WP_015600040.1	hypothetical protein	

Table S6. CDSs categorized into F4 in Figure 4.

Contig_gene_name	BLASTP			KEGG (KAAS)	
	gi	Refseq ID	BLASTP description	KO	KO description
CP002083-4	502979075	WP_013214051.1	hypothetical protein		
CP002083-21	502979092	WP_013214068.1	molecular chaperone Hsp33	K04083	hslO; molecular chaperone Hsp33
CP002083-52	502979123	WP_013214099.1	hypothetical protein		
CP002083-55	502979126	WP_013214102.1	oxidoreductase		
CP002083-72	502979143	WP_013214119.1	hypothetical protein		
CP002083-74	502979145	WP_013214121.1	hypothetical protein		
CP002083-76	502979147	WP_013214123.1	hypothetical protein		
CP002083-77	502979148	WP_013214124.1	ABC transporter substrate-binding protein		
CP002083-78	502979149	WP_013214125.1	GntR family transcriptional regulator		
CP002083-79	502979150	WP_013214126.1	hypothetical protein		
CP002083-81	502979152	WP_013214128.1	hypothetical protein		
CP002083-82	502979153	WP_013214129.1	dioxygenase subunit beta		
CP002083-83	502979154	WP_013214130.1	hypothetical protein		
CP002083-85	502979156	WP_013214132.1	globin	K06886	glbN; hemoglobin
CP002083-93	502979164	WP_013214140.1	aminoglycoside phosphotransferase	K07028	K07028
CP002083-94	502979165	WP_013214141.1	DNA-binding protein		
CP002083-96	502979167	WP_013214143.1	flavin reductase		
CP002083-97	502979168	WP_013214144.1	hypothetical protein		
CP002083-105	502979176	WP_013214152.1	integrase family protein		
CP002083-179	502979250	WP_013214226.1	glycosyl transferase	K07270	K07270; glycosyl transferase, family 25
CP002083-180	502979251	WP_013214227.1	polysaccharide biosynthesis protein	K03328	TC.PST; polysaccharide transporter, PST family
CP002083-181	502979252	WP_013214228.1	glycosyl transferase family 1		
CP002083-182	502979253	WP_013214229.1	hypothetical protein		
CP002083-184	502979255	WP_013214231.1	UDP-2-acetamido-2,6-dideoxy-beta-L-talose 4-dehydrogenase	K19068	
CP002083-185	502979256	WP_013214232.1	UDP-N-acetylglucosamine 2-epimerase	K01791	wecB; UDP-N-acetylglucosamine 2-epimerase [EC:5.1.3.14]
CP002083-186	502979257	WP_013214233.1	glycosyltransferase		
CP002083-189	502979260	WP_013214236.1	aminotransferase		
CP002083-192	502979263	WP_013214239.1	hypothetical protein	K06995	K06995
CP002083-210	502979281	WP_013214257.1	hypothetical protein		
CP002083-222	502979293	WP_013214269.1	cytochrome C peroxidase		
CP002083-226	502979297	WP_013214273.1	methyltransferase		
CP002083-227	502979298	WP_013214274.1	hypothetical protein		
CP002083-228	502979299	WP_013214275.1	hypothetical protein		
CP002083-343	502979414	WP_013214390.1	membrane protein		
CP002083-344	502979415	WP_013214391.1	transporter	K08978	TC.BAT1; bacterial/archaeal transporter family protein
CP002083-345	502979416	WP_013214392.1	hypothetical protein		
CP002083-359	502979430	WP_013214406.1	peptidase M15		
CP002083-361	502979432	WP_013214408.1	hypothetical protein		
CP002083-367	502979438	WP_013214414.1	membrane protein		
CP002083-377	502979448	WP_013214424.1	glycosyl hydrolase family 5		
CP002083-381	502979452	WP_013214428.1	hypothetical protein		
CP002083-395	502979466	WP_013214442.1	hypothetical protein		
CP002083-419	502979490	WP_013214466.1	hypothetical protein		
CP002083-420	502979491	WP_013214467.1	hypothetical protein	K07347	fimD, fimC, mrkC, htrE, cssD; outer membrane usher protein
CP002083-421	502979492	WP_013214468.1	hypothetical protein		
CP002083-422	502979493	WP_013214469.1	hypothetical protein		
CP002083-431	502979502	WP_013214478.1	antitermination factor NusG	K05785	rfaH; transcriptional antiterminator RfaH
CP002083-432	502979503	WP_013214479.1	colanic acid biosynthesis glycosyl transferase		
CP002083-433	502979504	WP_013214480.1	hypothetical protein		
CP002083-434	502979505	WP_013214481.1	acyl transferase	K03818	wcaF; putative colanic acid biosynthesis acetyltransferase WcaF [EC:2.3.1.-]
CP002083-437	502979508	WP_013214484.1	membrane protein		
CP002083-439	502979510	WP_013214486.1	glycosyl transferase family 1		
CP002083-442	502979513	WP_013214489.1	FkbM family methyltransferase		
CP002083-443	502979514	WP_013214490.1	FkbM family methyltransferase		
CP002083-445	502979516	WP_013214492.1	polysaccharide biosynthesis protein		
CP002083-446	502979517	WP_013214493.1	hypothetical protein		

CP002083-447	502979518	WP_013214494.1	molecular chaperone DnaJ		
CP002083-449	502979520	WP_013214496.1	capsular polysaccharide biosynthesis protein	K01104	E3.1.3.48; protein-tyrosine phosphatase [EC:3.1.3.48]
CP002083-450	502979521	WP_013214497.1	hypothetical protein		
CP002083-451	502979522	WP_013214498.1	polymerase		
CP002083-462	502979533	WP_013214509.1	hypothetical protein		
CP002083-463	502979534	WP_013214510.1	TetR family transcriptional regulator		
CP002083-466	502979537	WP_013214513.1	glycosyl transferase family 2		
CP002083-467	502979538	WP_013214514.1	hypothetical protein		
CP002083-468	502979539	WP_013214515.1	phytoene synthase	K02291	crtB; phytoene synthase [EC:2.5.1.32]
CP002083-469	502979540	WP_013214516.1	hypothetical protein		
CP002083-470	502979541	WP_013214517.1	squalene--hopene cyclase	K06045	shc, sqhC; squalene-hopene/tetraprenyl-beta-curcumene cyclase [EC:5.4.99.17 4.2.1.129]
CP002083-471	502979542	WP_013214518.1	phosphorylase		
CP002083-472	502979543	WP_013214519.1	radical SAM protein		
CP002083-475	502979546	WP_013214522.1	radical SAM protein		
CP002083-476	502979547	WP_013214523.1	hopanoid biosynthesis associated glycosyl transferase HpnI	K00720	UGCG; ceramide glucosyltransferase [EC:2.4.1.80]
CP002083-477	502979548	WP_013214524.1	ABC transporter	K04754	vacJ; lipoprotein
CP002083-478	502979549	WP_013214525.1	hopanoid biosynthesis associated membrane protein HpnM	K07323	ttg2; putative toluene tolerance protein
CP002083-480	502979551	WP_013214527.1	membrane protein		
CP002083-481	502979552	WP_013214528.1	hopanoid biosynthesis associated glycosyl transferase HpnI	K00720	UGCG; ceramide glucosyltransferase [EC:2.4.1.80]
CP002083-482	502979553	WP_013214529.1	hopanoid biosynthesis associated protein HpnK		
CP002083-483	502979554	WP_013214530.1	hypothetical protein		
CP002083-486	502979557	WP_013214533.1	membrane protein		
CP002083-491	502979562	WP_013214538.1	hypothetical protein		
CP002083-492	502979563	WP_013214539.1	MFS transporter		
CP002083-498	502979569	WP_013214545.1	hypothetical protein		
CP002083-499	502979570	WP_013214546.1	hypothetical protein		
CP002083-500	502979571	WP_013214547.1	hypothetical protein		
CP002083-501	502979572	WP_013214548.1	cupin		
CP002083-502	502979573	WP_013214549.1	hypothetical protein		
CP002083-503	502979574	WP_013214550.1	ferredoxin--NADP reductase	K00528	E1.18.1.2, fpr; ferredoxin--NADP+ reductase [EC:1.18.1.2]
CP002083-506	502979577	WP_013214553.1	protein glxC		
CP002083-512	502979583	WP_013214559.1	hypothetical protein	K07226	K07226; hypothetical protein
CP002083-515	502979586	WP_013214562.1	hypothetical protein		
CP002083-516	502979587	WP_013214563.1	hypothetical protein		
CP002083-517	502979588	WP_013214564.1	RNA polymerase subunit sigma-24	K03088	SIG3.2, rpoE; RNA polymerase sigma-70 factor, ECF subfamily
CP002083-520	502979591	WP_013214567.1	ligand-binding protein		
CP002083-521	502979592	WP_013214568.1	hypothetical protein		
CP002083-523	502979594	WP_013214570.1	hypothetical protein		
CP002083-524	502979595	WP_013214571.1	glutamate dehydrogenase	K00262	E1.4.1.4, gdhA; glutamate dehydrogenase (NADP+) [EC:1.4.1.4]
CP002083-525	502979596	WP_013214572.1	ABC transporter substrate-binding protein	K01999	livK; branched-chain amino acid transport system substrate-binding protein
CP002083-526	502979597	WP_013214573.1	GntR family transcriptional regulator		
CP002083-538	502979607	WP_013214583.1	hypothetical protein		
CP002083-539	502979608	WP_013214584.1	short-chain dehydrogenase	K07234	K07234; uncharacterized protein involved in response to NO
CP002083-540	502979609	WP_013214585.1	hypothetical protein		
CP002083-543	502979612	WP_013214588.1	membrane protein		
CP002083-545	502979614	WP_013214590.1	coenzyme PQQ synthesis D	K06138	pqqD; pyrroloquinoline quinone biosynthesis protein D
CP002083-546	502979615	WP_013214591.1	coenzyme PQQ biosynthesis protein A	K06135	pqqA; pyrroloquinoline quinone biosynthesis protein A
CP002083-553	502979622	WP_013214598.1	acyl carrier protein		
CP002083-555	502979624	WP_013214600.1	FkbH		
CP002083-556	502979625	WP_013214601.1	hypothetical protein	K02078	acpP; acyl carrier protein
CP002083-557	502979626	WP_013214602.1	3-octaprenyl-4-hydroxybenzoate carboxy-lyase	K03186	ubiX; 3-octaprenyl-4-hydroxybenzoate carboxy-lyase UbiX [EC:4.1.1.-]
CP002083-558	502979627	WP_013214603.1	3-octaprenyl-4-hydroxybenzoate carboxy-lyase	K03182	ubiD; 3-octaprenyl-4-hydroxybenzoate carboxy-lyase UbiD [EC:4.1.1.-]
CP002083-559	502979628	WP_013214604.1	lipid carrier		
CP002083-560	502979629	WP_013214605.1	protease	K08303	K08303; putative protease [EC:3.4.-.-]
CP002083-561	502979630	WP_013214606.1	protease		
CP002083-564	502979633	WP_013214609.1	hypothetical protein		
CP002083-565	502979634	WP_013214610.1	hypothetical protein		
CP002083-566	502979635	WP_013214611.1	membrane protein		
CP002083-568	502979637	WP_013214613.1	hypothetical protein	K07230	K07230

CP002083-571	502979640	WP_013214616.1	hypothetical protein		
CP002083-572	502979641	WP_013214617.1	hypothetical protein		
CP002083-574	502979643	WP_013214619.1	cytochrome C	K02305	norC; nitric oxide reductase subunit C
CP002083-575	502979644	WP_013214620.1	nitric oxide reductase	K04561	norB; nitric oxide reductase subunit B [EC:1.7.2.5]
CP002083-577	502979646	WP_013214622.1	protein norD	K02448	norD; nitric oxide reductase NorD protein
CP002083-578	502979647	WP_013214623.1	hypothetical protein		
CP002083-580	502979649	WP_013214625.1	hypothetical protein		
CP002083-582	502979651	WP_013214627.1	hypothetical protein		
CP002083-583	502979652	WP_013214628.1	short-chain dehydrogenase		
CP002083-588	502979657	WP_013214633.1	Crp/Fnr family transcription regulator	K01420	fnr; CRP/FNR family transcriptional regulator, anaerobic regulatory protein
CP002083-589	502979658	WP_013214634.1	histidine kinase		
CP002083-603	502979672	WP_013214648.1	hypothetical protein		
CP002083-604	502979673	WP_013214649.1	hypothetical protein		
CP002083-637	502979706	WP_013214682.1	hypothetical protein		
CP002083-725	502979792	WP_013214768.1	oxidoreductase FAD/NAD(P)-binding domain-containing protein	K00523	ascD, ddhD, rfbI; CDP-4-dehydro-6-deoxyglucose reductase [EC:1.17.1.1]
CP002083-741	502979808	WP_013214784.1	DNA polymerase IV	K02346	DPO4, dinB; DNA polymerase IV [EC:2.7.7.7]
CP002083-758	502979825	WP_013214801.1	hypothetical protein		
CP002083-763	502979830	WP_013214806.1	endoribonuclease L-PSP		
CP002083-806	502979873	WP_013214849.1	choloylglycine hydrolase	K01442	E3.5.1.24; choloylglycine hydrolase [EC:3.5.1.24]
CP002083-819	502979886	WP_013214862.1	CrtK	K07185	tspO; tryptophan-rich sensory protein
CP002083-836	502979903	WP_013214879.1	hypothetical protein		
CP002083-862	502979929	WP_013214905.1	Crp/Fnr family transcription regulator		
CP002083-869	502979936	WP_013214912.1	sulfurtransferase		
CP002083-871	502979938	WP_013214914.1	hypothetical protein		
CP002083-873	502979940	WP_013214916.1	hypothetical protein		
CP002083-874	502979941	WP_013214917.1	hypothetical protein		
CP002083-875	502979942	WP_013214918.1	hypothetical protein		
CP002083-877	502979944	WP_013214920.1	aminotransferase DegT		
CP002083-878	502979945	WP_013214921.1	GCN5 family acetyltransferase		
CP002083-882	502979949	WP_013214925.1	hypothetical protein		
CP002083-886	502979953	WP_013214929.1	autotransporter		
CP002083-887	502979954	WP_013214930.1	hypothetical protein		
CP002083-889	502979956	WP_013214932.1	hypothetical protein	K03088	SIG3.2, rpoE; RNA polymerase sigma-70 factor, ECF subfamily
CP002083-890	502979957	WP_013214933.1	hypothetical protein		
CP002083-913	502979980	WP_013214956.1	nitrate reductase A subunit beta	K00371	narH; nitrate reductase beta subunit [EC:1.7.99.4]
CP002083-914	502979981	WP_013214957.1	nitrate reductase	K00373	narJ; nitrate reductase delta subunit
CP002083-915	502979982	WP_013214958.1	nitrate reductase	K00374	narI; nitrate reductase gamma subunit [EC:1.7.99.4]
CP002083-918	502979985	WP_013214961.1	hypothetical protein		
CP002083-919	502979986	WP_013214962.1	hypothetical protein		
CP002083-920	502979987	WP_013214963.1	membrane protein		
CP002083-921	502979988	WP_013214964.1	glycoprotein gp100		
CP002083-966	502980033	WP_013215009.1	hypothetical protein		
CP002083-969	502980036	WP_013215012.1	hypothetical protein		
CP002083-970	502980037	WP_013215013.1	polyhydroxyalkanoic acid synthase		
CP002083-971	502980038	WP_013215014.1	hypothetical protein		
CP002083-972	502980039	WP_013215015.1	hypothetical protein		
CP002083-973	502980040	WP_013215016.1	hypothetical protein		
CP002083-974	502980041	WP_013215017.1	glucokinase		
CP002083-979	502980046	WP_013215022.1	glycosyl hydrolase		
CP002083-980	502980047	WP_013215023.1	signal peptide protein		
CP002083-982	502980049	WP_013215025.1	oxidoreductase	K00459	E1.13.12.16; nitronate monooxygenase [EC:1.13.12.16]
CP002083-1040	502980107	WP_013215083.1	cytochrome C		
CP002083-1041	502980108	WP_013215084.1	hypothetical protein		
CP002083-1042	502980109	WP_013215085.1	hypothetical protein		
CP002083-1043	502980110	WP_013215086.1	hypothetical protein		
CP002083-1046	502980113	WP_013215089.1	IclR family transcriptional regulator		
CP002083-1047	502980114	WP_013215090.1	uroporphyrin-III C-methyltransferase		
CP002083-1053	502980120	WP_013215096.1	hypothetical protein		
CP002083-1057	502980124	WP_013215100.1	hypothetical protein		

CP002083-1058	502980125	WP_013215101.1	hypothetical protein		
CP002083-1061	502980128	WP_013215104.1	hypothetical protein		
CP002083-1062	502980129	WP_013215105.1	hypothetical protein		
CP002083-1063	502980130	WP_013215106.1	photosystem reaction center subunit H		
CP002083-1064	502980131	WP_013215107.1	photosystem reaction center subunit H		
CP002083-1100	502980167	WP_013215143.1	biopolymer transporter ExbD		
CP002083-1131	502980198	WP_013215174.1	cytochrome C	K08738	CYC; cytochrome c
CP002083-1139	502980206	WP_013215182.1	AraC family transcriptional regulator		
CP002083-1142	502980209	WP_013215185.1	hypothetical protein		
CP002083-1144	502980211	WP_013215187.1	hypothetical protein		
CP002083-1145	502980212	WP_013215188.1	FAD-dependent oxidoreductase		
CP002083-1156	502980223	WP_013215199.1	hypothetical protein		
CP002083-1180	502980247	WP_013215223.1	hypothetical protein		
CP002083-1250	502980317	WP_013215293.1	hypothetical protein		
CP002083-1262	502980329	WP_013215305.1	hypothetical protein		
CP002083-1353	502980420	WP_013215396.1	amylo-alpha-1,6-glucosidase		
CP002083-1382	502980449	WP_013215425.1	hypothetical protein		
CP002083-1406	502980473	WP_013215449.1	SAM-dependent methyltransferase		
CP002083-1409	502980476	WP_013215452.1	putrescine/spermidine ABC transporter substrate-binding protein	K11073	potF; putrescine transport system substrate-binding protein
CP002083-1418	502980485	WP_013215461.1	hypothetical protein		
CP002083-1472	502980539	WP_013215515.1	coenzyme PQQ biosynthesis protein A		
CP002083-1480	502980547	WP_013215523.1	hypothetical protein		
CP002083-1486	502980553	WP_013215529.1	hypothetical protein		
CP002083-1487	502980554	WP_013215530.1	hypothetical protein		
CP002083-1490	502980557	WP_013215533.1	magnesium transporter MgtC	K07507	mgtC; putative Mg2+ transporter-C (MgtC) family protein
CP002083-1491	502980558	WP_013215534.1	MFS transporter		
CP002083-1495	502980562	WP_013215538.1	hypothetical protein		
CP002083-1496	502980563	WP_013215539.1	hypothetical protein		
CP002083-1501	502980568	WP_013215544.1	hypothetical protein		
CP002083-1583	502980594	WP_013215570.1	transglycosylase		
CP002083-1584	502980595	WP_013215571.1	cytochrome C biogenesis protein		
CP002083-1603	502980614	WP_013215590.1	cytochrome C biogenesis protein	K06196	ccdA; cytochrome c-type biogenesis protein
CP002083-1645	502980656	WP_013215632.1	hypothetical protein		
CP002083-1646	502980657	WP_013215633.1	GTP-binding protein HSR1		
CP002083-1728	502980739	WP_013215715.1	luciferase		
CP002083-1736	502980747	WP_013215723.1	hypothetical protein		
CP002083-1738	502980749	WP_013215725.1	hypothetical protein		
CP002083-1740	502980751	WP_013215727.1	hypothetical protein		
CP002083-1746	502980757	WP_013215733.1	hypothetical protein		
CP002083-1747	502980758	WP_013215734.1	thioesterase		
CP002083-1774	502980785	WP_013215761.1	GCN5 family acetyltransferase		
CP002083-1796	502980807	WP_013215783.1	ethyl tert-butyl ether degradation protein EthD		
CP002083-1804	502980815	WP_013215791.1	mucin		
CP002083-1805	502980816	WP_013215792.1	membrane protein	K09790	K09790; hypothetical protein
CP002083-1806	502980817	WP_013215793.1	GCN5-like N-acetyltransferase		
CP002083-1816	502980827	WP_013215803.1	universal stress protein UspA		
CP002083-1826	502980837	WP_013215813.1	hypothetical protein		
CP002083-1830	502980841	WP_013215817.1	hypothetical protein		
CP002083-1832	502980843	WP_013215819.1	hypothetical protein		
CP002083-1846	502980857	WP_013215833.1	Crp/Fnr family transcription regulator	K01420	fnr; CRP/FNR family transcriptional regulator, anaerobic regulatory protein
CP002083-1848	502980859	WP_013215835.1	cytochrome C		
CP002083-1851	502980862	WP_013215838.1	hypothetical protein		
CP002083-1852	502980863	WP_013215839.1	thiamine biosynthesis protein ApbE	K03734	apbE; thiamine biosynthesis lipoprotein
CP002083-1853	502980864	WP_013215840.1	NosL family protein	K19342	
CP002083-1854	502980865	WP_013215841.1	copper ABC transporter permease	K19341	
CP002083-1856	502980867	WP_013215843.1	carbohydrate-binding protein	K07218	nosD; nitrous oxidase accessory protein
CP002083-1857	502980868	WP_013215844.1	nitrous-oxide reductase	K00376	nosZ; nitrous-oxide reductase [EC:1.7.2.4]
CP002083-1858	502980869	WP_013215845.1	FMN-binding protein	K19339	
CP002083-1859	502980870	WP_013215846.1	hypothetical protein		

CP002083-1861	502980872	WP_013215848.1	hypothetical protein		
CP002083-1863	502980874	WP_013215850.1	hypothetical protein		
CP002083-1864	502980875	WP_013215851.1	pilus biosynthesis protein PilZ		
CP002083-1866	502980877	WP_013215853.1	hypothetical protein		
CP002083-1869	502980880	WP_013215856.1	SinR		
CP002083-1921	502980932	WP_013215908.1	hypothetical protein		
CP002083-1927	502980938	WP_013215914.1	hypothetical protein		
CP002083-1949	502980960	WP_013215936.1	rod shape-determining protein RodA		
CP002083-1995	502981006	WP_013215982.1	hypothetical protein		
CP002083-1998	502981009	WP_013215985.1	transporter		
CP002083-1999	502981010	WP_013215986.1	3-hydroxyalkanoate synthetase		
CP002083-2000	502981011	WP_013215987.1	hypothetical protein		
CP002083-2001	502981012	WP_013215988.1	phosphate acetyltransferase	K00625	E2.3.1.8, pta; phosphate acetyltransferase [EC:2.3.1.8]
CP002083-2002	502981013	WP_013215989.1	acetate kinase	K00925	ackA; acetate kinase [EC:2.7.2.1]
CP002083-2007	502981018	WP_013215994.1	permease		
CP002083-2008	502981019	WP_013215995.1	hypothetical protein		
CP002083-2009	502981020	WP_013215996.1	hypothetical protein		
CP002083-2010	502981021	WP_013215997.1	histidine kinase		
CP002083-2011	502981022	WP_013215998.1	membrane protein		
CP002083-2012	502981023	WP_013215999.1	hypothetical protein		
CP002083-2013	502981024	WP_013216000.1	hypothetical protein		
CP002083-2015	502981026	WP_013216002.1	hypothetical protein	K09792	K09792; hypothetical protein
CP002083-2016	502981027	WP_013216003.1	cytochrome oxidase maturation protein Cbb3		
CP002083-2018	502981029	WP_013216005.1	FixH		
CP002083-2019	502981030	WP_013216006.1	(Fe-S)-binding protein		
CP002083-2020	502981031	WP_013216007.1	cytochrome CBB3	K00406	ccoP; cytochrome c oxidase cbb3-type subunit III
CP002083-2021	502981032	WP_013216008.1	cytochrome oxidase	K00407	ccoQ; cytochrome c oxidase cbb3-type subunit IV
CP002083-2022	502981033	WP_013216009.1	peptidase S41	K00405	ccoO; cytochrome c oxidase cbb3-type subunit II
CP002083-2023	502981034	WP_013216010.1	cytochrome C oxidase	K00404	ccoN; cytochrome c oxidase cbb3-type subunit I [EC:1.9.3.1]
CP002083-2028	502981039	WP_013216015.1	5-methyltetrahydropteroyltriglutamate--homocysteine methyltransferase	K00549	metE; 5-methyltetrahydropteroyltriglutamate--homocysteine methyltransferase [EC:2.1.1.14]
CP002083-2067	502981078	WP_013216054.1	hypothetical protein		
CP002083-2076	502981087	WP_013216063.1	hypothetical protein		
CP002083-2078	502981089	WP_013216065.1	peptidylprolyl isomerase	K01802	E5.2.1.8; peptidylprolyl isomerase [EC:5.2.1.8]
CP002083-2079	502981090	WP_013216066.1	hypothetical protein		
CP002083-2081	502981092	WP_013216068.1	coenzyme PQQ biosynthesis protein A		
CP002083-2100	502981111	WP_013216087.1	hypothetical protein		
CP002083-2109	502981120	WP_013216096.1	hypothetical protein		
CP002083-2110	502981121	WP_013216097.1	hypothetical protein		
CP002083-2111	502981122	WP_013216098.1	ABC transporter substrate-binding protein	K09945	K09945; hypothetical protein
CP002083-2113	502981124	WP_013216100.1	hypothetical protein		
CP002083-2144	502981155	WP_013216131.1	methylated-DNA-protein-cysteine methyltransferase	K07443	ybaZ; methylated-DNA-protein-cysteine methyltransferase related protein
CP002083-2146	502981157	WP_013216133.1	phosphoesterase		
CP002083-2147	502981158	WP_013216134.1	Crp/Fnr family transcription regulator		
CP002083-2149	502981160	WP_013216136.1	photosystem reaction center subunit H		
CP002083-2174	502981185	WP_013216161.1	hypothetical protein		
CP002083-2178	502981189	WP_013216165.1	peptidase M48		
CP002083-2179	502981190	WP_013216166.1	hypothetical protein		
CP002083-2182	502981193	WP_013216169.1	hypothetical protein		
CP002083-2193	502981204	WP_013216180.1	MFS transporter		
CP002083-2231	502981242	WP_013216218.1	membrane protein		
CP002083-2232	502981243	WP_013216219.1	hypothetical protein		
CP002083-2233	502981244	WP_013216220.1	hypothetical protein		
CP002083-2234	502981245	WP_013216221.1	hypothetical protein		
CP002083-2235	502981246	WP_013216222.1	hypothetical protein		
CP002083-2236	502981247	WP_013216223.1	hypothetical protein		
CP002083-2237	502981248	WP_013216224.1	hypothetical protein		
CP002083-2242	502981253	WP_013216229.1	membrane protein		
CP002083-2244	502981255	WP_013216231.1	NnrS family protein	K07234	K07234; uncharacterized protein involved in response to NO
CP002083-2245	502981256	WP_013216232.1	phenylalanyl-tRNA synthetase subunit alpha		

CP002083-2246	502981257	WP_013216233.1	prolyl-tRNA synthetase	K03976	ebsC; putative transcription regulator
CP002083-2249	502981260	WP_013216236.1	AMP-dependent synthetase		
CP002083-2250	502981261	WP_013216237.1	hypothetical protein		
CP002083-2313	502981324	WP_013216300.1	major facilitator transporter		
CP002083-2340	502981351	WP_013216327.1	membrane protein	K08995	K08995; putative membrane protein
CP002083-2341	502981352	WP_013216328.1	hypothetical protein		
CP002083-2370	502981381	WP_013216357.1	histone deacetylase		
CP002083-2372	502981383	WP_013216359.1	NUDIX hydrolase		
CP002083-2373	502981384	WP_013216360.1	hypothetical protein		
CP002083-2391	502981402	WP_013216378.1	hypothetical protein		
CP002083-2396	502981407	WP_013216383.1	hypothetical protein		
CP002083-2397	502981408	WP_013216384.1	membrane protein		
CP002083-2398	502981409	WP_013216385.1	hypothetical protein		
CP002083-2400	502981411	WP_013216387.1	hypothetical protein		
CP002083-2401	502981412	WP_013216388.1	membrane protein		
CP002083-2445	502981456	WP_013216432.1	glutamine amidotransferase	K05520	pfpI; protease I [EC:3.2.-.-]
CP002083-2455	502981466	WP_013216442.1	flagellar export protein FliJ		
CP002083-2463	502981474	WP_013216450.1	hypothetical protein		
CP002083-2485	502981496	WP_013216472.1	plasmid stabilization protein		
CP002083-2490	502981501	WP_013216477.1	hypothetical protein		
CP002083-2512	502981523	WP_013216499.1	hypothetical protein		
CP002083-2524	502981535	WP_013216511.1	hypothetical protein		
CP002083-2526	502981537	WP_013216513.1	hypothetical protein		
CP002083-2527	502981538	WP_013216514.1	membrane protein		
CP002083-2580	502981591	WP_013216567.1	sulfate transporter	K03321	TC.SULP; sulfate permease, SulP family
CP002083-2581	502981592	WP_013216568.1	XRE family transcriptional regulator		
CP002083-2614	502981625	WP_013216601.1	50S ribosomal protein L36	K02919	RP-L36, MRPL36, rpmJ; large subunit ribosomal protein L36
CP002083-2654	502981665	WP_013216641.1	membrane protein		
CP002083-2676	502981687	WP_013216663.1	DNA gyrase inhibitor	K09862	K09862; hypothetical protein
CP002083-2694	502981705	WP_013216681.1	multidrug transporter MatE		
CP002083-2713	502981724	WP_013216700.1	hypothetical protein		
CP002083-2727	502981738	WP_013216714.1	chaperone transmembrane protein	K11741	sugE; quaternary ammonium compound-resistance protein SugE
CP002083-2754	502981765	WP_013216741.1	hypothetical protein		
CP002083-2768	502981779	WP_013216755.1	peptidase M4		
CP002083-2774	502981785	WP_013216761.1	protein hupE	K03192	ureJ; urease accessory protein
CP002083-2776	502981787	WP_013216763.1	urease accessory protein UreG	K03189	ureG; urease accessory protein
CP002083-2792	502981803	WP_013216779.1	hypothetical protein		
CP002083-2793	502981804	WP_013216780.1	hypothetical protein		
CP002083-2794	502981805	WP_013216781.1	hypothetical protein		
CP002083-2795	502981806	WP_013216782.1	ferric reductase		
CP002083-2817	502981828	WP_013216804.1	hypothetical protein		
CP002083-2892	502981903	WP_013216879.1	hypothetical protein		
CP002083-2898	502981909	WP_013216885.1	ATPase	K01546	kdpA; K+-transporting ATPase ATPase A chain [EC:3.6.3.12]
CP002083-2900	502981911	WP_013216887.1	ATPase	K01548	kdpC; K+-transporting ATPase ATPase C chain [EC:3.6.3.12]
CP002083-2960	502981971	WP_013216947.1	hypothetical protein		
CP002083-3000	502982011	WP_013216987.1	hypothetical protein		
CP002083-3002	502982013	WP_013216989.1	hypothetical protein		
CP002083-3004	502982015	WP_013216991.1	hypothetical protein		
CP002083-3007	502982018	WP_013216994.1	hypothetical protein		
CP002083-3008	502982019	WP_013216995.1	MarR family transcriptional regulator		
CP002083-3010	502982021	WP_013216997.1	precorrin 6A synthase	K02228	cobF; precorrin-6A synthase [EC:2.1.1.152]
CP002083-3012	502982023	WP_013216999.1	NAD(P)H quinone oxidoreductase	K03809	wrbA; Trp repressor binding protein
CP002083-3013	502982024	WP_013217000.1	diguanylate cyclase	K15975	K15975; glyoxalase family protein
CP002083-3014	502982025	WP_013217001.1	phospholipase	K06999	K06999; phospholipase/carboxylesterase
CP002083-3015	502982026	WP_013217002.1	hypothetical protein		
CP002083-3017	502982028	WP_013217004.1	DNA-binding protein	K04047	dps; starvation-inducible DNA-binding protein
CP002083-3018	502982029	WP_013217005.1	ceramide glucosyltransferase	K00720	UGCG; ceramide glucosyltransferase [EC:2.4.1.80]
CP002083-3020	502982031	WP_013217007.1	hypothetical protein		
CP002083-3084	502982095	WP_013217071.1	sarcosine oxidase subunit delta	K00304	soxD; sarcosine oxidase, subunit delta [EC:1.5.3.1]

CP002083-3086	502982097	WP_013217073.1	sarcosine oxidase subunit gamma	K00305	soxG; sarcosine oxidase, subunit gamma [EC:1.5.3.1]
CP002083-3123	502982134	WP_013217110.1	hypothetical protein		
CP002083-3138	502982149	WP_013217125.1	hypothetical protein		
CP002083-3146	502982157	WP_013217133.1	hypothetical protein		
CP002083-3159	502982170	WP_013217146.1	hypothetical protein		
CP002083-3167	502982178	WP_013217154.1	hypothetical protein		
CP002083-3168	502982179	WP_013217155.1	hypothetical protein		
CP002083-3180	502982191	WP_013217167.1	transposase		
CP002083-3181	502982192	WP_013217168.1	hypothetical protein		
CP002083-3208	502982219	WP_013217195.1	pyridoxal kinase	K00868	pdxK, pdxY; pyridoxine kinase [EC:2.7.1.35]
CP002083-3257	502982268	WP_013217244.1	addiction module protein		
CP002083-3262	502982273	WP_013217249.1	aldo/keto reductase		
CP002083-3276	502982287	WP_013217263.1	hypothetical protein		
CP002083-3299	502982310	WP_013217286.1	peptidase M50		
CP002083-3391	502982402	WP_013217378.1	hypothetical protein		
CP002083-3394	502982405	WP_013217381.1	hypothetical protein		
CP002083-3396	502982407	WP_013217383.1	hypothetical protein		
CP002083-3399	502982410	WP_013217386.1	histidine kinase		
CP002083-3400	502982411	WP_013217387.1	hypothetical protein		
CP002083-3407	502982418	WP_013217394.1	hypothetical protein		
CP002083-3428	502982439	WP_013217415.1	hypothetical protein		
CP002083-3483	502982494	WP_013217470.1	hypothetical protein	K14205	mprF, fmtC; phosphatidylglycerol lysyltransferase [EC:2.3.2.3]
CP002083-3484	502982495	WP_013217471.1	virulence factor		
CP002083-3497	502982508	WP_013217484.1	LuxR family transcriptional regulator		
CP002083-3498	502982509	WP_013217485.1	hypothetical protein		
CP002083-3499	502982510	WP_013217486.1	hypothetical protein		

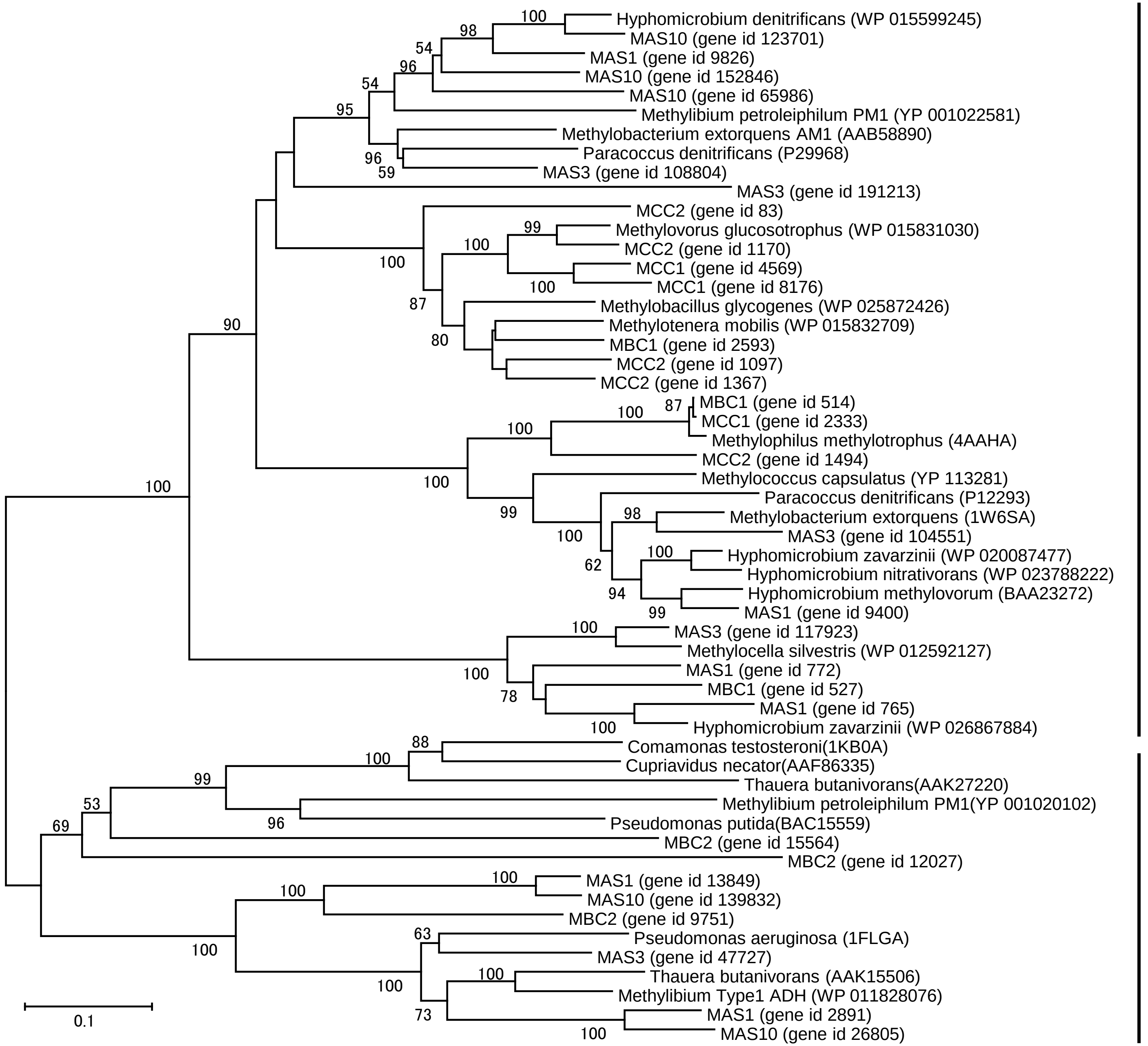

Fig. S1. Neighbor-joining tree based on amino-acid sequences showing phylogenetic relationships among alcohol dehydrogenases of the PQQ-dependent methanol/ethanol dehydrogenase family retrieved from metagenome contigs. All alcohol dehydrogenases retrieved from the contigs using the HMMER web server are included. Bootstrap values (100 trials, only > 50 are shown) are indicated at branching points. MDH, methanol dehydrogenase; ADH, non-methanol alcohol dehydrogenase.

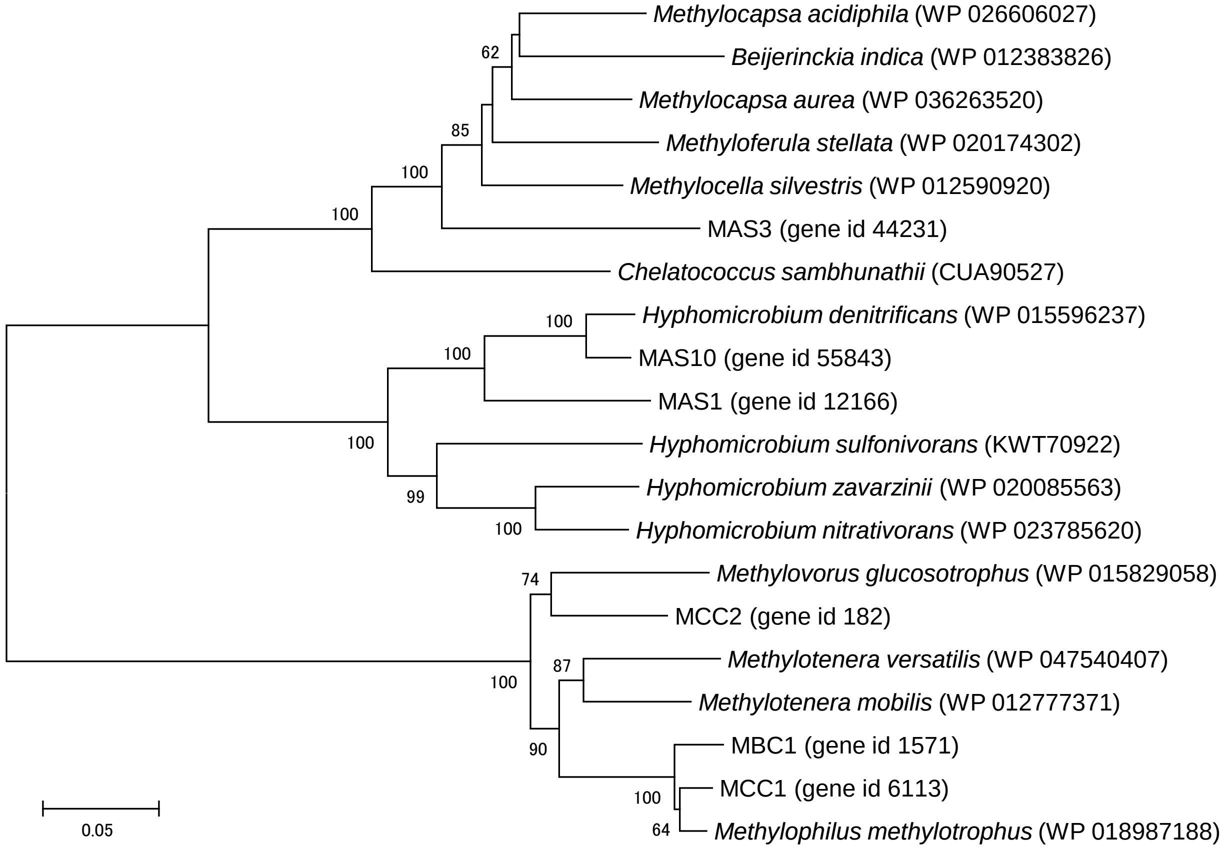

Fig. S2. Neighbor-joining tree based on amino-acid sequences of DNA gyrase subunit B showing phylogenetic relationships among bin genomes and their relatives. Bootstrap values (100 trials, only > 50 are shown) are indicated at branching points.

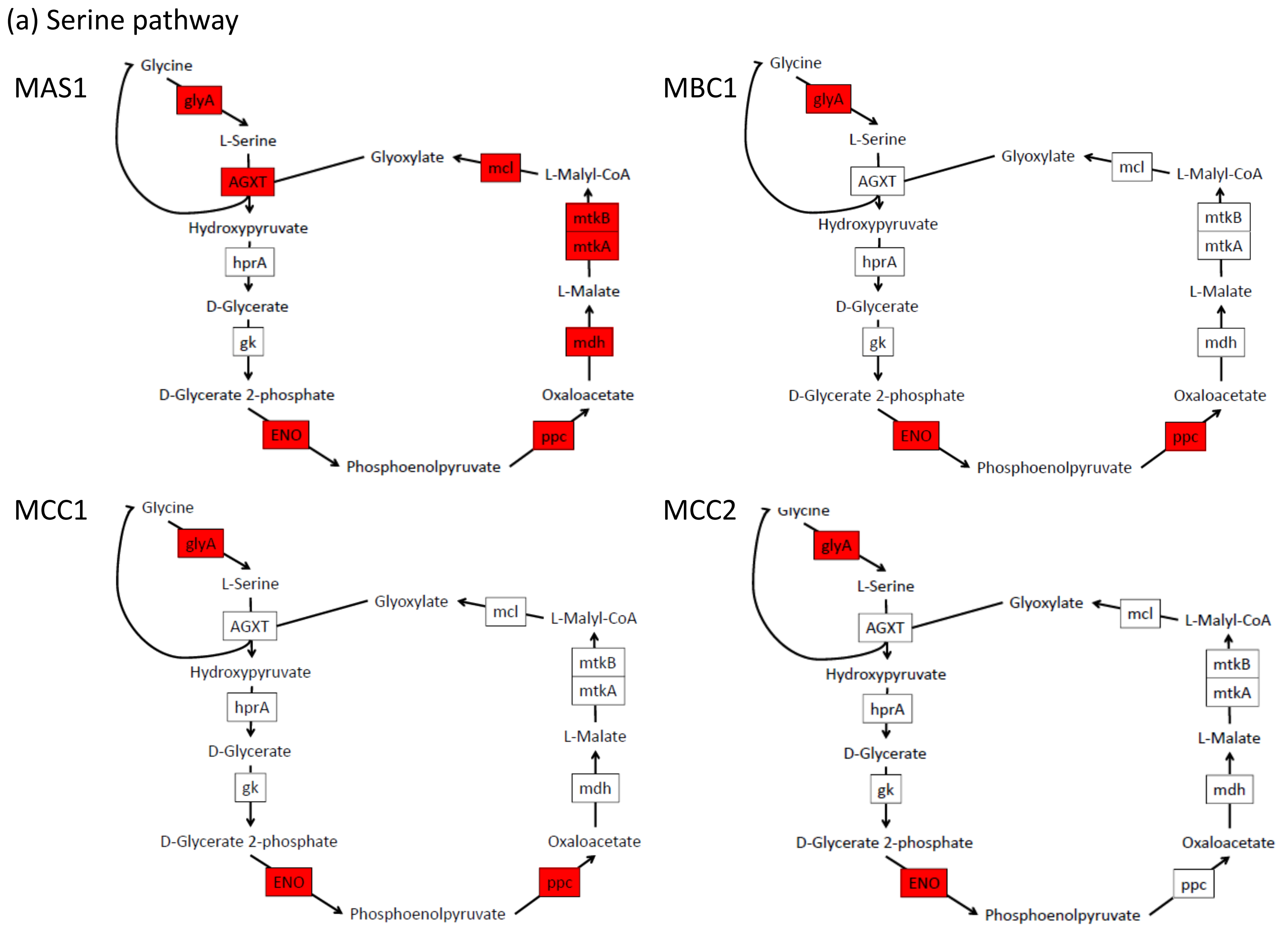

Fig. S3. Comparisons in genes in the C1-assimilation pathways among the major bin genomes. (a) the serine pathway, (b) the ethylmalonyl-CoA (EMC) pathway, (c) the ribulose monophosphate (RuBP) cycle. Genes present in a bin genome are marked red.

(a) EMC pathway

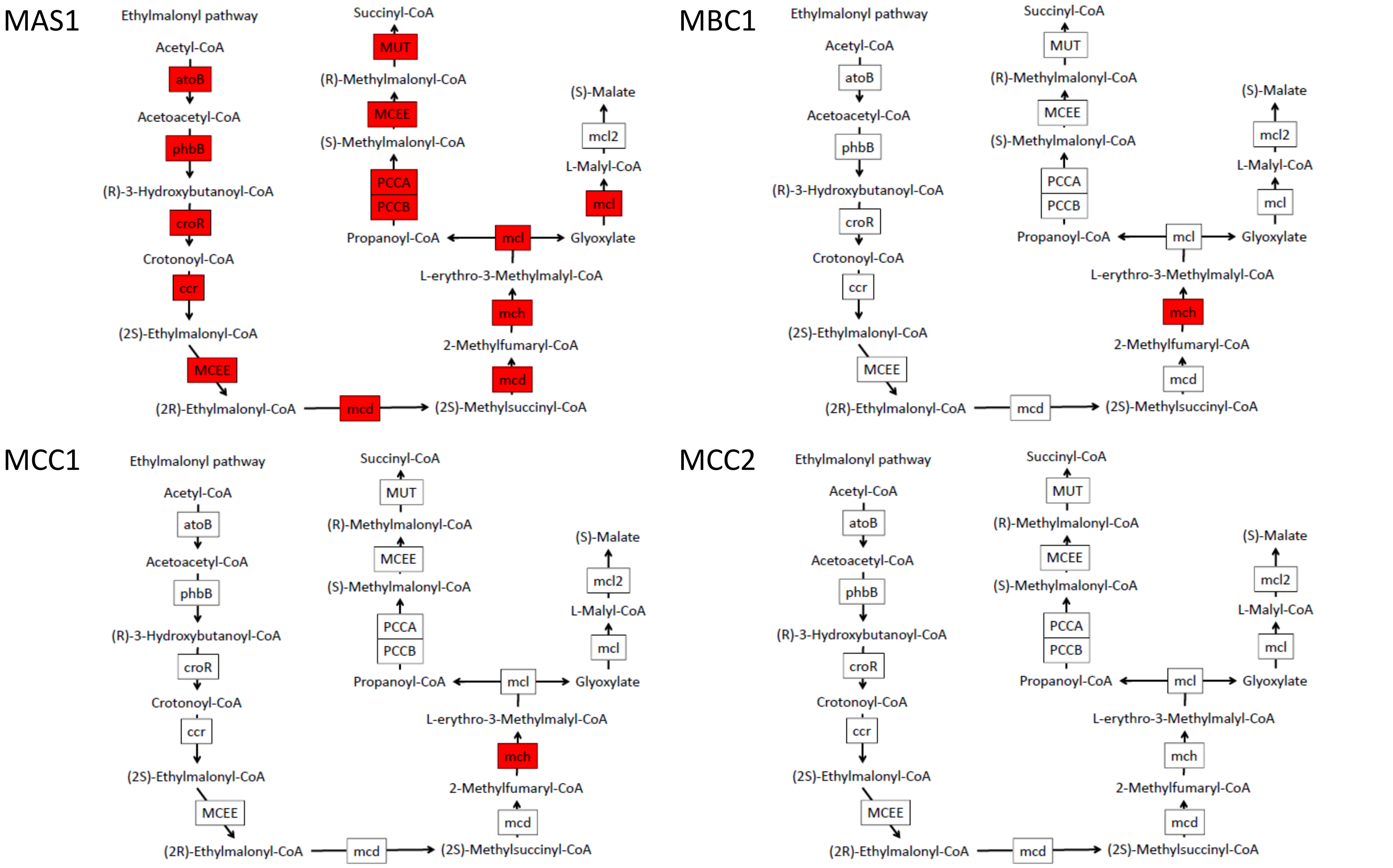

Fig. S3. Continued.

(a) RuBP cycle

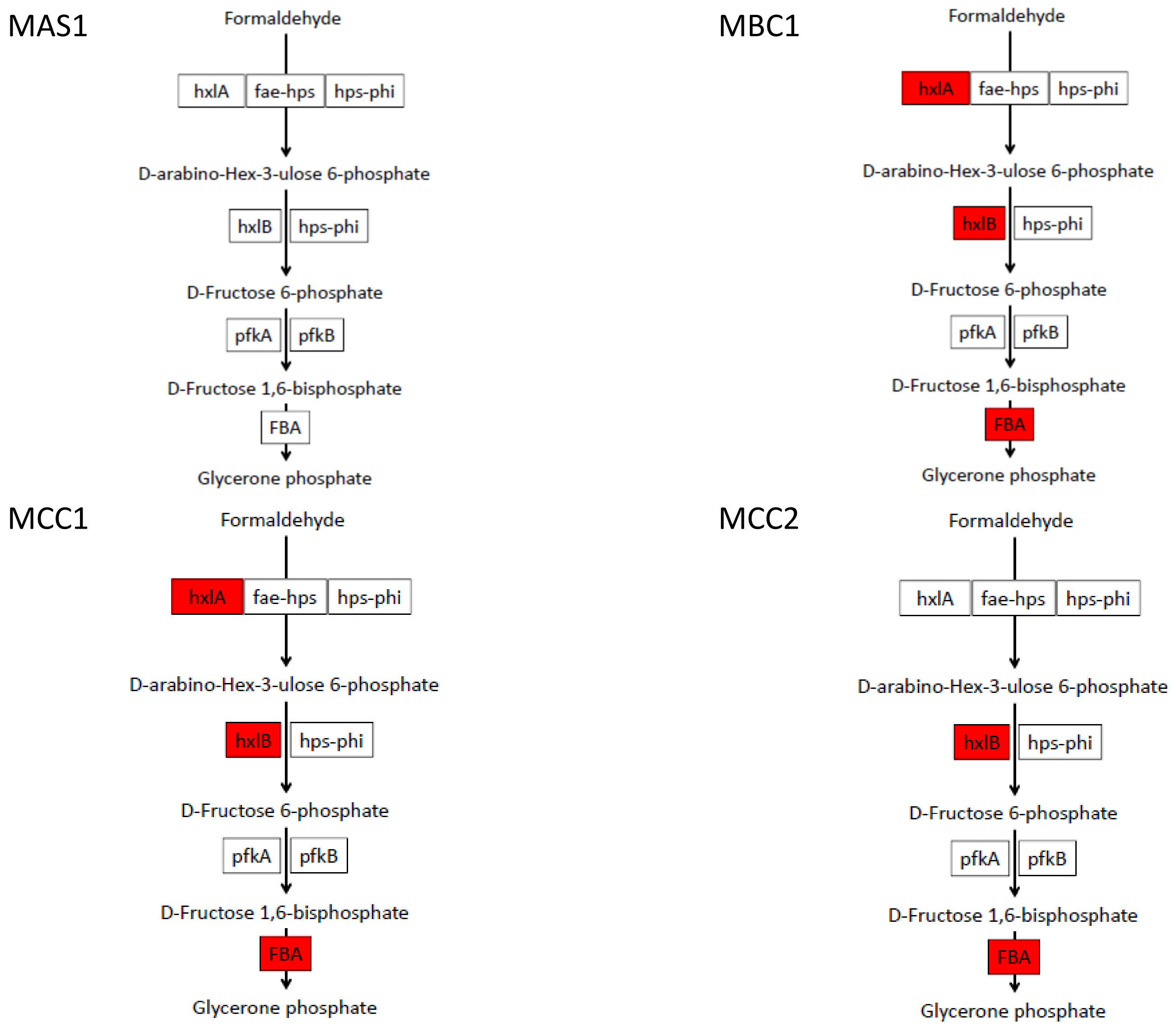

Fig. S3. Continued.

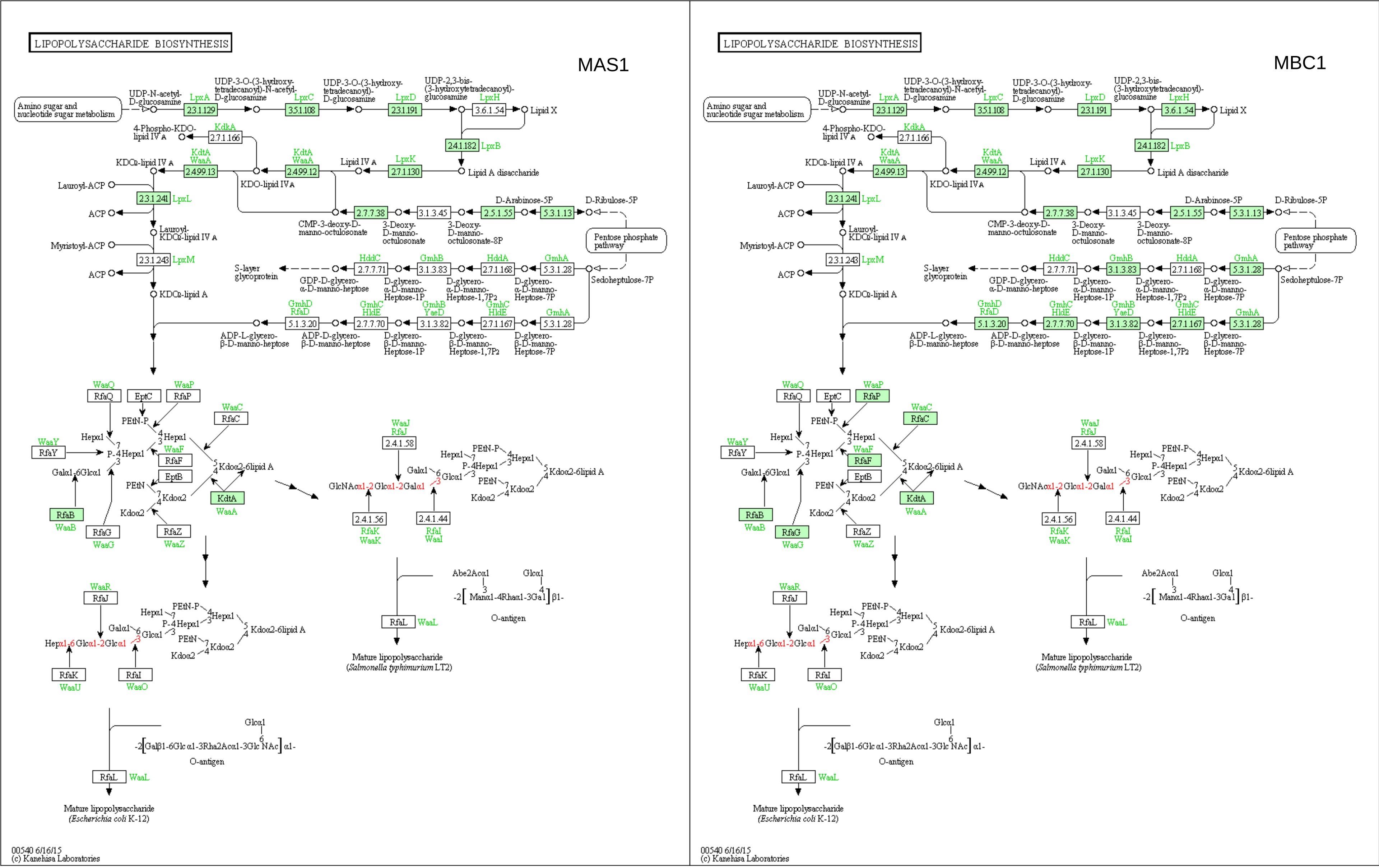

Fig. S4. Comparison in genes for the lipopolysaccharide biosynthesis pathways in the major bin genomes. Genes present in bin genomes are marked green .

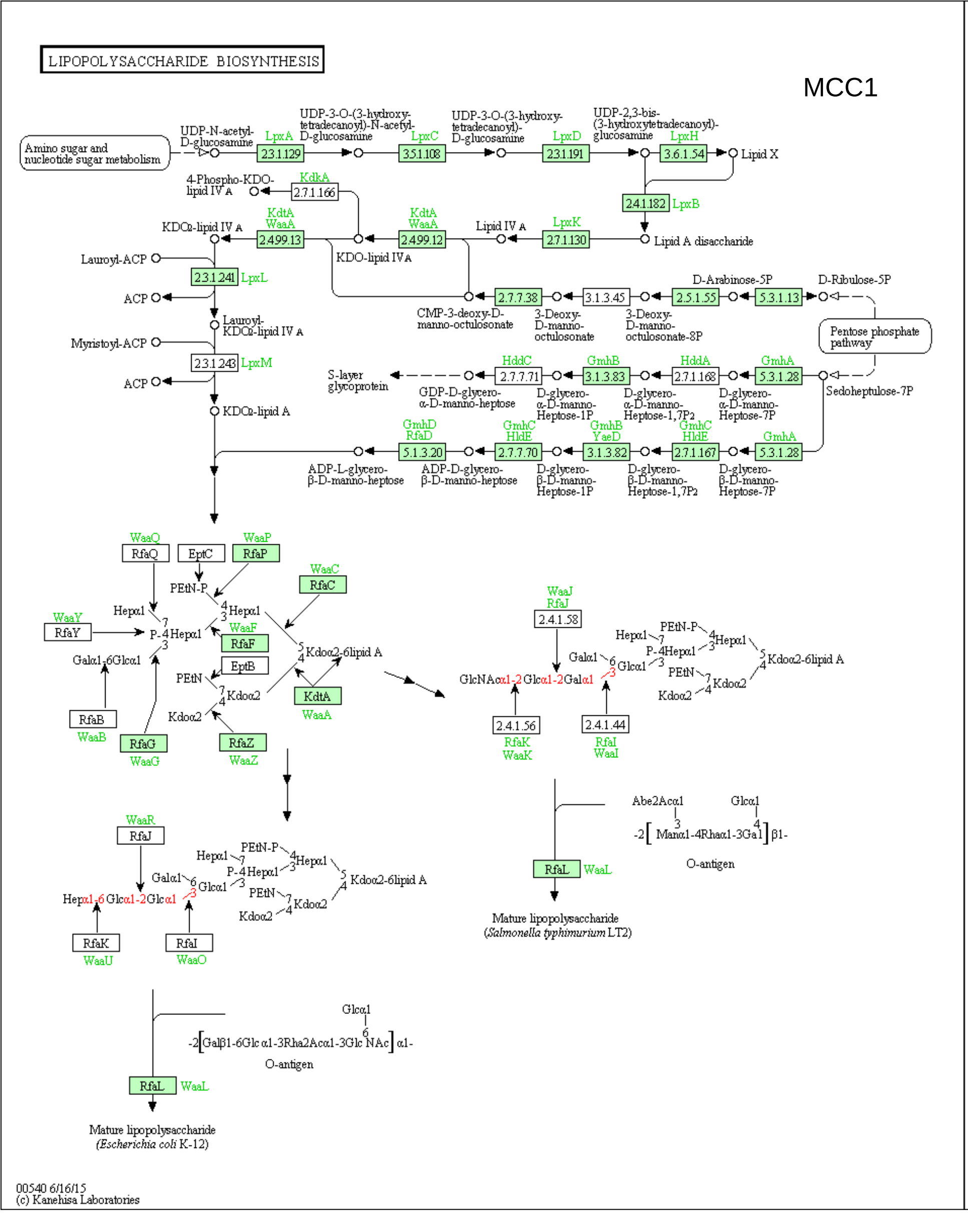

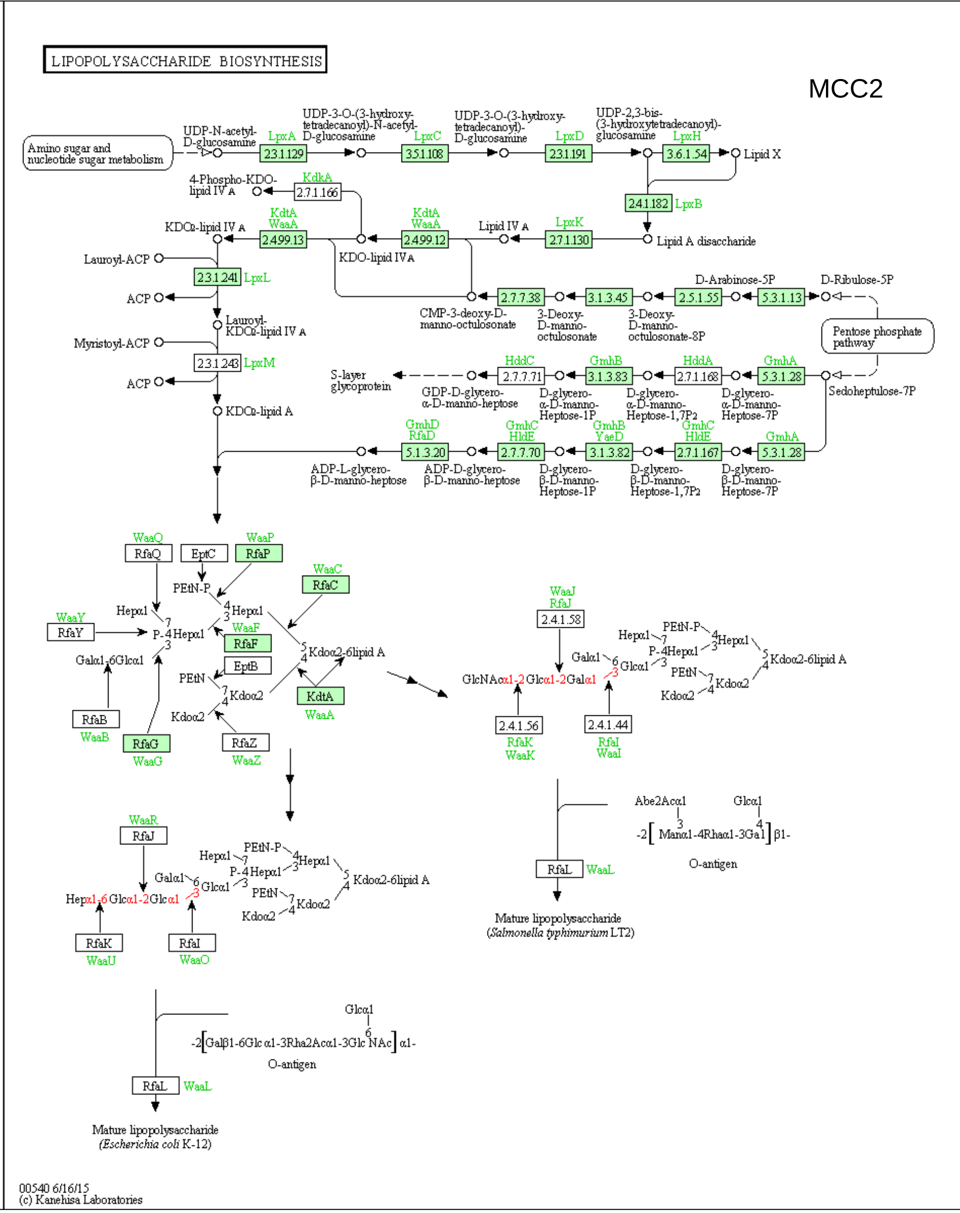

Fig. S4.Continued.
